# Synthesis, In Silico, and Biological Evaluation of Non‐Hydroxamate Benzoic Acid–Based Derivatives as Potential Histone Deacetylase Inhibitors (HDACi)

**DOI:** 10.1002/cbdv.202501492

**Published:** 2025-09-02

**Authors:** Nedaa A. Abd Al Rahim, Ammar A. Razzak Mahmood, Lubna H. Tahtamouni, Randa M. Bawadi, Ayah Y. Almasri, Marya A. Hamad, Nour A. Hussein, Salem R. Yasin, Abdulrahman M. Saleh

**Affiliations:** ^1^ Department of Pharmaceutical Chemistry College of Pharmacy, University of Baghdad Baghdad Iraq; ^2^ Department of Biology and Biotechnology Faculty of Science The Hashemite University Zarqa Jordan; ^3^ Department of Biochemistry and Molecular Biology College of Natural Sciences, Colorado State University Fort Collins Colorado USA; ^4^ Department of Medical Laboratory Faculty of Allied Sciences Zarqa University Zarqa Jordan; ^5^ Department of Basic Dental Sciences Faculty of Dentistry The Hashemite University Zarqa Jordan; ^6^ Department of Pharmaceutical Chemistry Faculty of Pharmacy Cairo University Cairo Egypt; ^7^ Infection Control and Epidemiology Surveillance Unit Aweash El‐Hagar Family Medicine Center, Ministry of Health and Population (MOHP) Mansoura Egypt

**Keywords:** apoptosis, inhibitors, oxadiazole, Schiff bases, thioamides

## Abstract

Unregulated epigenetic modifications, including histone acetylation/deacetylation mediated by histone acetyltransferases (HATs) and histone deacetylases (HDACs), contribute to cancer progression. HDACs, often overexpressed in cancer, downregulate tumor suppressor genes, making them crucial targets for treatment. This work aimed to develop non‐hydroxamate benzoic acid–based HDAC inhibitors (HDACi) with comparable effect to the currently four FDA‐approved HDACi, which are known for their poor solubility, poor distribution, and significant side effects. All compounds were structurally verified using FTIR, ^1^HNMR, ^13^CNMR, and mass spectrometry. In silico analysis showed that compound **A3bn** (3‐chloro‐4‐((2‐(2‐(4‐hydroxybenzylidene) hydrazinyl)‐2‐oxoethyl)amino)benzoic acid) has strong binding affinity towards HDAC2, HDAC6, and HDAC8 and exhibits molecular similarity to trichostatin and SAHA (HDACi). **A3bn** achieved IC_50_ values comparable to SAHA against MCF‐7 (20.3 vs. 39.2 µM) and K562 (42.0 vs. 36.1 µM) cancer cells. Western blot analysis confirmed that **A3bn** inhibited H3 and H4 deacetylation. Additionally, **A3bn** induced the extrinsic apoptotic pathway via caspase 8 activation, leading to cell death. Its enhanced activity across HDAC isoforms may result from its hydrophilic linker, facilitating zinc coordination. In conclusion, **A3bn** demonstrated efficacy similar to FDA‐approved HDACi and represents a promising candidate for further optimization. Future studies will focus on structural modifications to enhance potency and selectivity at lower concentrations.

## Graphical abstract

Compound **A3bn** exhibited strong binding affinities for HDAC2, HDAC6, and HDAC8. **A3bn**’s anticancer IC_50_ values were similar to those of SAHA. Cell death resulted from the extrinsic apoptotic pathway. Compound **A3bn,** a hydrophilic linker, which promotes zinc coordination, may be the cause of its increased activity across HDAC isoforms.

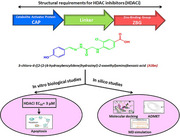

## 1 Introduction

Cancer is characterized by uncontrolled growth and spread of cells driven by multifactorial causes, including epigenetic modifications. Histone modifications, including acetylation, methylation, phosphorylation, and ubiquitination, are examples of epigenetics—changes in gene expression that do not involve changes to the DNA sequence [1]. Histone acetylation, mediated by histone acetyltransferases (HATs), and histone deacetylation, mediated by histone deacetylases (HDACs), are both posttranslational modifications that involve the addition or removal of an acetyl group to histones [2]. DNA compaction is reduced and gene expression is increased when HATs add a negatively charged acetyl group, which reduces the attraction between the negatively charged DNA and the modified histones. HDACs, on the other hand, remove an acetyl group from histones, reducing their negative charge and increasing their positive charge. This results in a stronger binding to DNA, which decreases gene expression [3].

**FIGURE 1:**
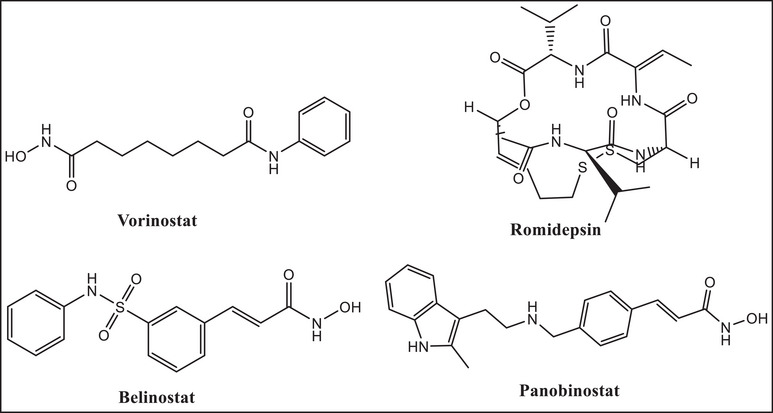
The structures of the four approved HDAC inhibitors (vorinostat, romidepsin, belinostat, and panobinostat).

HDACs are categorized as zinc‐dependent (Classes I, II, and IV) or zinc‐independent (Class III) enzymes. Tumor suppressor genes are downregulated by HDACs, which are frequently overexpressed in cancer cells. HDACs are therefore crucial targets for cancer therapy [4]. Only four drugs—vorinostat, romidepsin, belinostat, and panobinostat (Figure 1)—have received FDA approval as HDAC inhibitors (HDACi) and are utilized as anticancer drugs, despite 20 years of synthetic attempts. HDACis, such as vorinostat (also known as SAHA) and romidepsin, are praised for their efficacy in treating T‐cell lymphoma [5]; nevertheless, they are ineffective in treating solid tumors [6]. By chelating the zinc ions necessary for the activity of zinc‐dependent HDACs, these inhibitors attach to the active site of HDAC enzymes and target them specifically.

HDACis have a wide spectrum of adverse effects, from headaches to serious conditions like myelosuppression, much like any other medication [7]. Furthermore, HDACi solubility and delivery, which have a direct impact on treatment outcome, are still suboptimal. For example, the hydroxamic acid vorinostat is categorized as a Class IV medication in the biopharmaceutics classification system (BCS) due to its poor permeability (log *p* of 1.9) and poor water solubility (about 0.19 mg/mL) [8]. Therefore, finding novel HDACis is essential.

Oxadiazole is a five‐member heterocyclic ring containing one oxygen and two nitrogen heteroatoms [9]. Heterocyclic compounds represent a significant class of bioactive molecules, with more than 85% of physiologically active chemical compounds containing at least one heterocyclic component [10]. Among these, the 1,3,4‐oxadiazole and 1,2,4‐oxadiazole derivatives exhibit favorable physical, chemical, and pharmacokinetic properties compared to oxadiazole, significantly enhancing their pharmacological activity via hydrogen bonding with biomacromolecules [11]. These derivatives have demonstrated potent anticancer activity by inhibiting different HDAC enzymes, with substituent properties such as electronegativity and substitution patterns (ortho > meta > para) playing critical roles in their efficacy [12].

Thioamides, sulfur‐containing compounds with unique properties, are versatile building blocks in heterocyclic chemistry and serve as isosteres of canonical amide bonds. They are stronger hydrogen bond donors and exhibit greater metal affinity than their amide counterparts, making them valuable in developing metal complexes and chelators [13]. Thioamide derivatives are widely used to modify bioactive compounds, with several therapeutic agents containing this motif already approved for clinical use or under investigation in clinical trials [14].

Schiff bases are macrocyclic or macro‐acyclic ligands with significant coordinating abilities. They are synthesized through the condensation of primary amines with aldehydes or ketones [15, 16, 17]. Their donor properties can be adjusted by incorporating nitrogen, sulfur, oxygen, or phosphorus [18]. Schiff bases and their metal complexes display diverse biological properties, including antibacterial, antifungal, anti‐inflammatory, and antitumor activities [19, 20].

The present study aimed to develop novel non‐hydroxamate benzoic acid‐based HDACis incorporating benzohydrazone (**A3an** and **A3bn**), 1,3,4‐oxadiazole (**A4an–4dn**), or hydrazine‐1‐carbothioamide moieties (**A5an–5cn**). These inhibitors were designed to enhance selectivity, potency, and pharmacokinetic properties while minimizing side effects, paving the way for improved cancer therapies.

## 2 Results and Discussion

### 2.1 Chemistry

**SCHEME 1:**
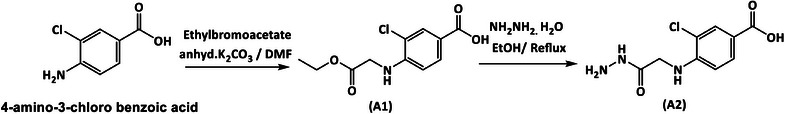
Synthesis of the intermediates **A1** and **A2**.

**SCHEME 2:**
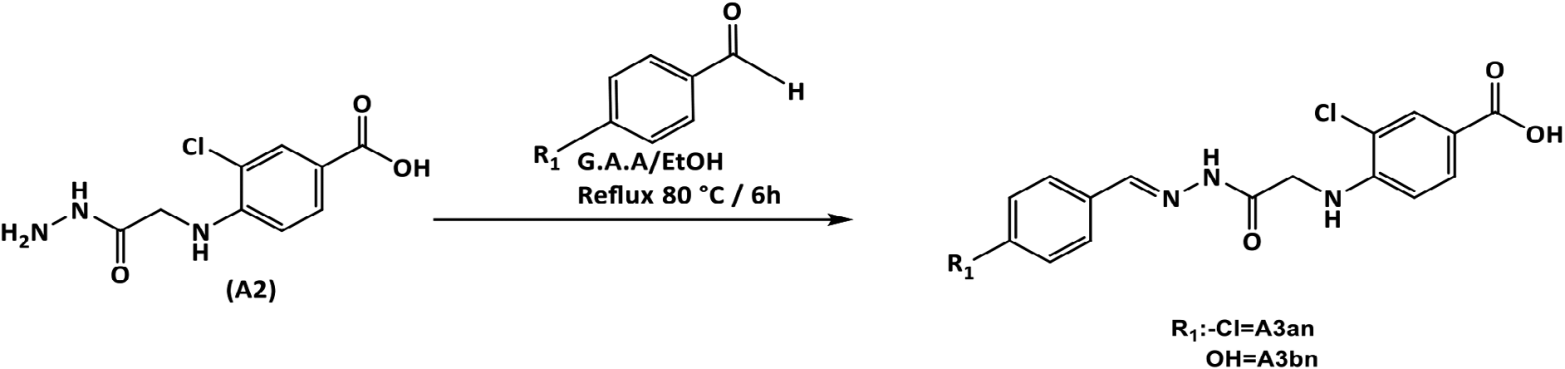
Synthesis of benzohydrazone derivatives **A3an** and **A3bn**.

**SCHEME 3:**
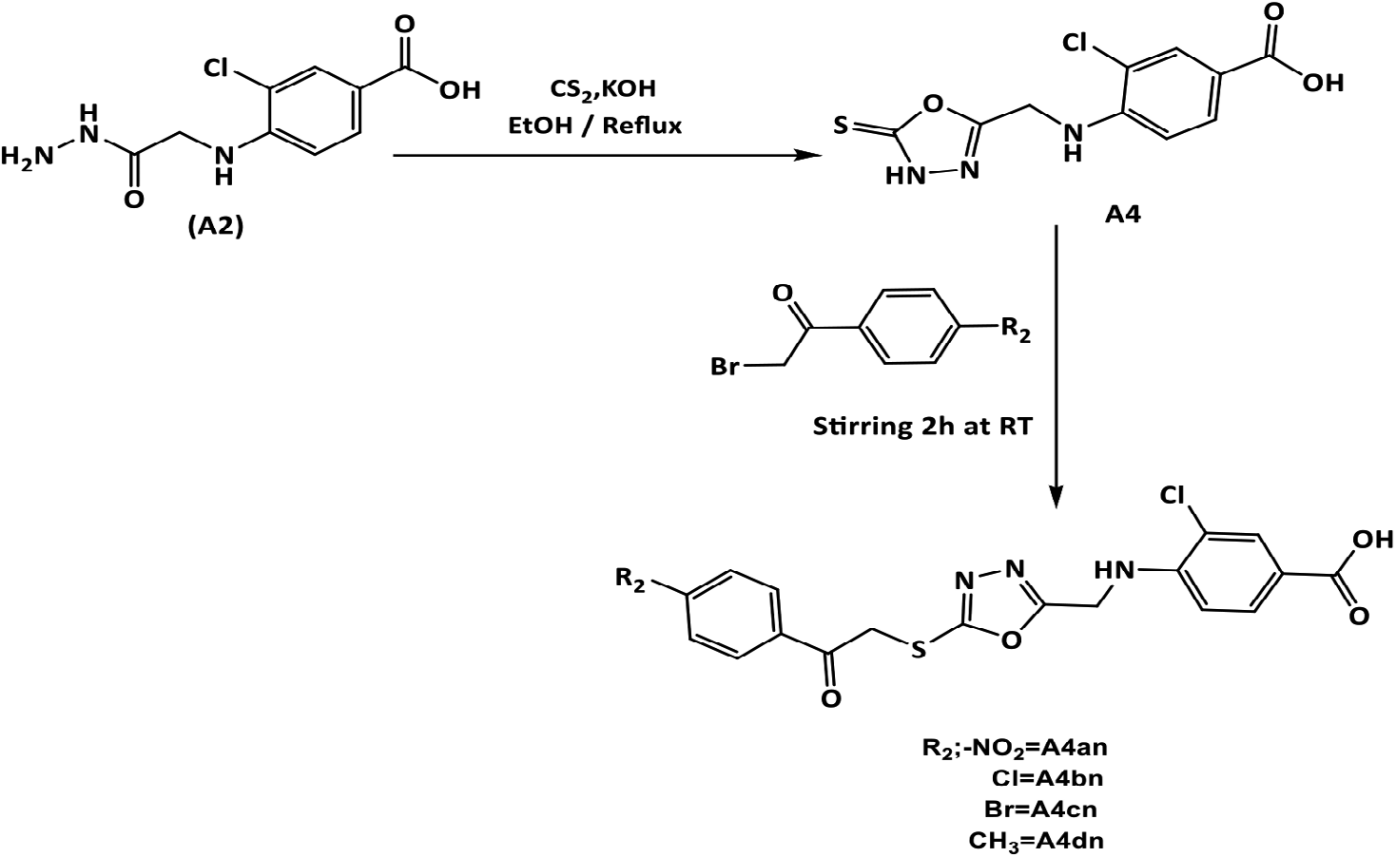
Synthesis of 1,3,4‐oxadiazole‐2 thione (**A4**) and phenacyl bromide derivatives (**A4an–4dn**).

**SCHEME 4:**
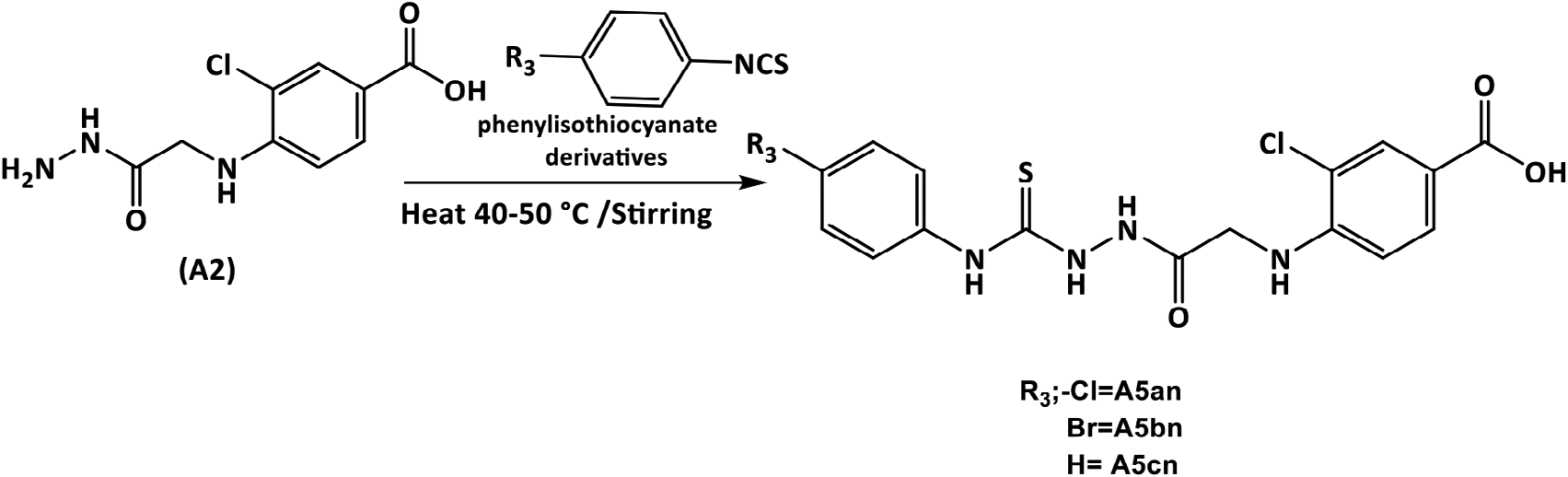
Synthesis of hydrazine‐1‐carbothioamide derivatives (**A5an–5cn**).

The desired compounds are prepared as outlined in Schemes 1, 2, 3, 4.

The attenuated total reflection‐Fourier transform IR (ATR‐FTIR) spectra revealed that compound **A1** (Figure S1A) showed a characteristic absorption band at ύ = 1624 cm^−1^ corresponding to (C═O) ester stretching. Compound **A2** (Figure S2A) exhibited distinctive absorption bands at 3317 and 3290 cm^−1^ representing *asym* and *sym* (NH) stretching of *primary* amine (NH_2_). Compound **A4** (Figure S5A) displayed an absorption peak at ύ = 3336 cm^−1^ indicating (NH‐thioamide) stretching. The infrared (IR) spectra of compounds **A3an** (Figure S3A) and **A3bn** (Figure S4A) showed distinctive bands at ύ = 1600 and 1593 cm^−1^, due to (C═N) stretching, respectively. The IR spectra of compounds **A4an–4dn** (Figures A6A–S9A) displayed bands at ύ = 1670–1678 cm^−1^ for (C═O) amide stretching. Compounds **A5an–5cn** IR spectra (Figures S10A–S12A) showed bands at ύ = 3309–3356 cm^−1^, due to (NH) stretching of thioamide.

The ^1^HNMR spectra of compounds **A1‐5cn** indicated that compound **A1** (Figure S1B) showed a distinct singlet at *δ* = 4.8 ppm for (NH─CH_2_), triplet at *δ* = 1.20 ppm for CH_3,_ and quartet at *δ* = 4.15 ppm, attributed to O─CH_2_ of ester. A peak at *δ* = 6.36 ppm, appearing as a broad singlet with an integration of two protons (NH_2_ **^+^** R) and lacking the proton of the carboxylic hydroxyl (OH), may be ascribed to the production of a zwitterion [21].

Compound **A2** (Figure S2B) showed a prominent singlet at *δ* = 9.44 ppm, attributed to (NH), and a broad singlet attributed to NH_2_ at *δ* = 5.86 ppm. Compounds **A3an** (Figure S3B) and **A3bn** (Figure S4B) revealed singlet band at *δ* = 11.46 and 11.37 ppm, respectively, owing to NH group, and a characteristic singlet appeared at *δ* = 8.38 and 8.30 ppm, attributed to imine moiety (CH═N), respectively. In addition, compound **A3bn** displayed a distinct singlet at *δ* = 9.89 ppm for phenolic OH. Compound **A4** (Figure S5B) showed a broad singlet at *δ* = 14.46 ppm for (**NH** C═S). The ^1^HNMR spectrum of **A4** did not display the proton of carboxylic acid and that could be attributed to deuterium exchange [22]. Compounds **A4an–4dn** (Figures S6B–S9B) showed a unique singlet at *δ* = 6.19–6.24 ppm, due to NH─CH_2_. Noteworthy, compound **A4dn** (Figure S9B) exhibited a singlet at *δ* = 2.40 ppm, indicating (*R* ^2^ = 4‐CH_3_) group. Additionally, compounds **A5an–5cn** (Figures S10B–S12B) revealed broad singlet integrating for two protons at *δ* = 6.01 ppm, due to aromatic‐**NH_2_^+^**‐zwitterion. The NH groups were seen at *δ* = 10.21, 9.78, and 9.68 ppm for **A5an** (Figure S10B), and compound **A5bn** (Figure S11B) displayed the NH group at *δ* = 10.21, 9.77, and 9.69 ppm, whereas **A5cn** (Figure S12B) displayed the NH groups at *δ* = 10.21, 9.74, and 9.57 ppm. Each aromatic proton for the target compounds showed up in its specific location at *δ* = 6.81–7.86 ppm. Finally, **A2**, **A4, A3an, A3bn**, and **A4bn‐dn** did not display all the protons of NH groups, due to –NH– deuterium exchange [23, 24].

The ^13^CNMR spectra exhibited that compound **A1** (Figure S1C) showed signals at *δ* = 167.94 ppm for (C═O), *δ* = 13.98 ppm corresponding to the CH_3_, and at 60.74 ppm corresponding to (CH_2_). Compound **A2** (Figure S2C) showed a signal at *δ* = 165.09 ppm attributed to COOH and CONHNH and corresponding to two unresolved peaks, and at *δ* = 40.01 ppm due to CH_2_ moiety. Compounds **A3an** (Figure S3C) and **A3bn** (Figure S4C) showed new signals at *δ* = 148.03 and 147.79 ppm, respectively, owing to (C═N) imine, and signals at *δ* = 168.36 and 161.78, corresponding to the (C═O) amide group. Compound **A4** (Figure S5C) exhibited signal at *δ* = 176.74 due to (C═S) group. The derivatives **A4an–4dn** (Figures S6C–S9C) exhibited signals at *δ* = 40.31–40.49 ppm for (CH_2_–S) and *δ* = 192.01–192.87 ppm for (C═O) ketone group. Compound **A4dn** (Figure S9C) showed characteristic CH_3_ signal at *δ* = 21.25 ppm. Compounds **A5an–5cn** (Figures S10C–S12C) exhibited a signal at *δ* = 181.07–181.84 ppm due to (C═S) group and a signal at *δ* = 164.96 ppm due to COOH and CONHNH, corresponding to two unresolved peaks for **A5an**.

The mass spectra showed an adduct of [M]^+^ for compounds **A3an** (Figure S3D), **A3bn** (Figure S4D), and **A5an** (Figure S10D), an adduct of [M + 1]**^+^** for compounds **A1** (Figure S1D) and **A4** (Figure S5D), an adduct of [M + 2]**^+^** for compounds **A2** (Figure S2D) and **A4an–4dn** (Figures S6D–S9D), and an adduct of [M + 3]**^+^** for compounds **A5bn** (Figure S11D) and **A5cn** (Figure S12D). These results proved successful preparation of the newly synthesized compounds.

## 3 In Silico Studies

### 3.1 Molecular Docking Study

All of the new compounds were tested against class I (HDAC1‐3, HDAC8) and class II (HDAC4‐7, HDAC9‐10) HDACs. Compound **A3bn** showed affinity towards three of the HDACs: HDAC2, HDAC6, and HDAC8 (PDB codes: 4ly1, 8cj7, and 3sff, respectively), and compound **A4** showed affinity towards HDAC2 and HDAC6, compound **A3an** showed affinity towards HDAC2, compound **A4cn** showed affinity towards HDAC6, and compound **A5cn** showed affinity towards HDAC8.

#### 3.1.1 Molecular Docking of the Target Compounds Against HDAC2

**FIGURE 2:**
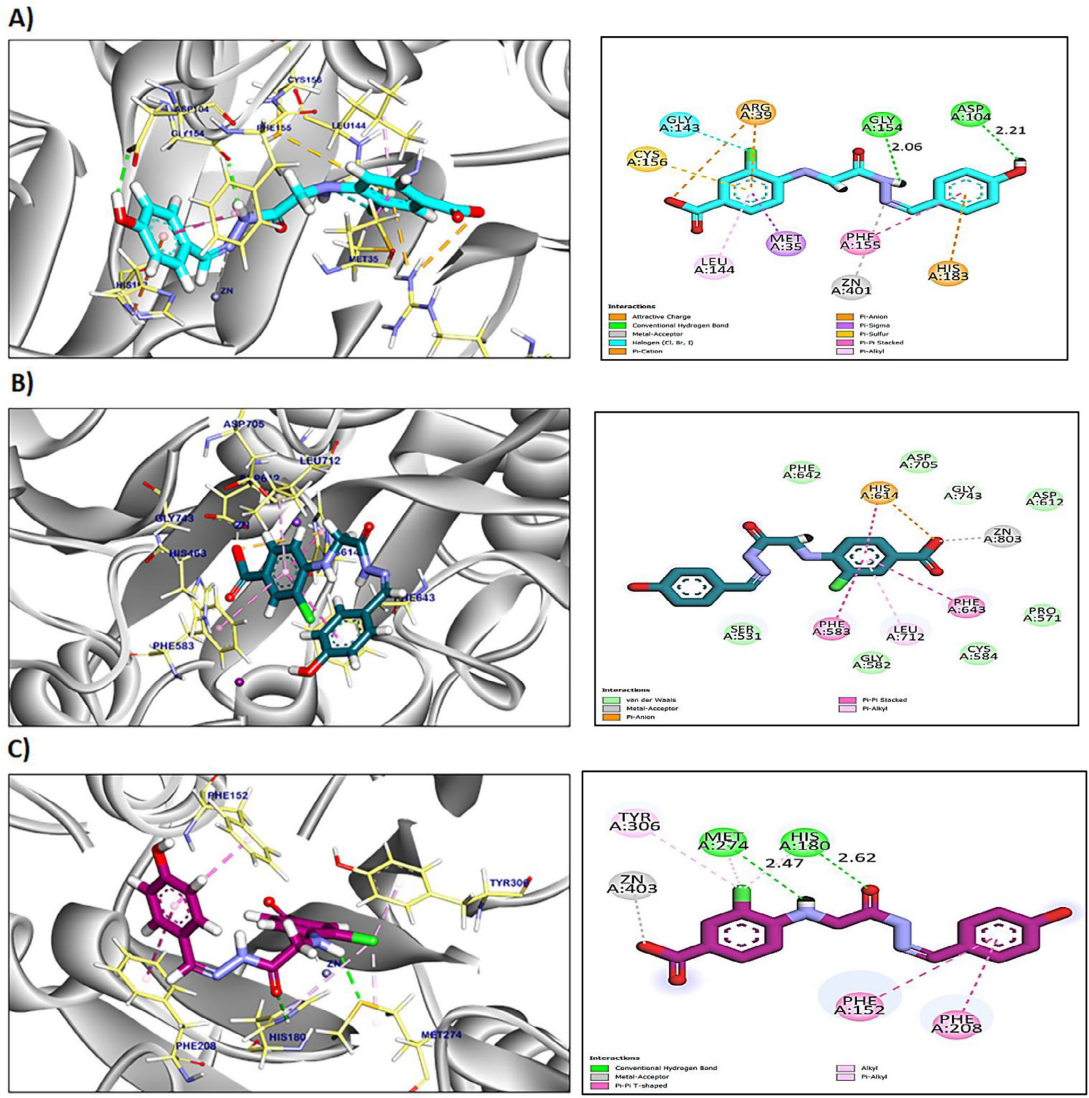
Two‐ and three‐dimensional (2D and 3D) mapping of the proposed binding mode of compound **A3bn** in the binding pocket of (A) HDAC2, (B) HDAC6, and (C) HDAC8.

Results presented in Table S2 show that compounds **A3an**, **A3bn**, and **A4** exhibited higher binding affinities towards HDAC2 when compared to the two reference HDACis, SAHA and trichostatin. Compound **A3an** interacted with Phe210, Leu276, Phe155, Leu144, and Cys156 by six hydrophobic π‐interactions and formed one hydrogen bond with Gly154 with a distance of 2.61 Å. Additionally, the pharmacophoric metal acceptor hydrazone group interacted by metal ion interaction with ZN401 (Figure S13A). Compound **A3bn** formed seven hydrophobic π‐interactions and ionic bond with Arg39, Cys156, Gly143, Met35, Leu144, Phe155, and His183, and two hydrogen bonds with Gly154 and Asp104 with distances of 2.06 and 2.21 Å, respectively. Moreover, the pharmacophoric group interacted with ZN401 by attractive metal ion bond (Figure 2A). Compound **A4** interacted by seven hydrophobic π‐interactions with His183, Cys156, Met35, Arg39, Leu144, and Phe114; additionally, the interactions were supported by three hydrogen bonds and metal ion interaction with Ala141, His183, His146, and ZN401, respectively, with distances of 3.00, 2.73, and 2.95 Å (Figure S13B).

The reference compounds (SAHA and trichostatin) exhibited an affinity score of −7.04 and −8.54 kcal/mol, respectively. SAHA formed six hydrophobic π‐interactions with Phe210, Leu276, His183, Phe155, Cys156, and Leu144 (Figure S13C). Furthermore, it interacted with His183, Met35, Arg39, and ZN401 by three hydrogen bonds and metal ion interactions with distances of 2.92, 2.38, and 2.93 Å. Although trichostatin formed thirteen hydrophobic π‐interactions with Leu144, Met35, Cys156, Leu276, His183, Phe210, Phe155, His146, and His145, additionally, two hydrogen bonds and metal ion bond were observed with His183, Gly305, and ZN401 with distances of 3.29 and 2.43 Å (Figure S13D).

#### 3.1.2 Molecular Docking of Target Compounds Against HDAC6

Three compounds (**A3bn**, **A4cn**, and **A4**) exhibited higher binding affinities towards HDAC6 when compared to the two reference HDACis, SAHA and trichostatin (Table S3). Compound **A3bn** exhibited an affinity score of −8.37 kcal/mol against HDAC6. It interacted with Leu712, Phe643, Phe583, His614, and ZN803 by five hydrophobic π‐interactions and metal ion interactions (Figure 2B). The affinity score of compound **A4cn** was −8.16 kcal/mol against HDAC6. Compound **A4cn** showed five hydrophobic π‐interactions with His463, Phe643, Phe583, and His614; furthermore, two hydrogen bonds and metal ion interaction were observed with His463, His614, and ZN803 with bond length of 2.97 and 3.07 Å, respectively (Figure S14A).

Compound **A4**’s affinity score was −8.15 kcal/mol against HDAC6. It formed four hydrophobic π‐linkages with His614, Phe583, Phe643, and Phe642. Moreover, it formed two hydrogen bonds supported with one metal interaction with His614, Leu712, and ZN803 with bond length of 3.05 and 2.92 Å (Figure S14B).

The reference compounds (SAHA and trichostatin) exhibited an affinity score of −7.86 and −7.87 kcal/mol, respectively. SAHA formed three hydrophobic π‐interactions with Phe643, His614, and Phe155. Furthermore, it interacted with His614, His573, Gly582, and ZN803 by three hydrogen bonds and metal ion interactions with distances of 2.90, 2.33, and 2.25 Å (Figure S14C). However, trichostatin formed four hydrophobic π‐interactions with Phe583, Phe643, and His614. Additionally, four hydrogen bonds and metal ion bond were observed with Gly582, Asp612, His573, His614, and ZN401 with distances of 2.34, 2.66, 2.73, and 3.02 Å (Figure S14D).

#### 3.1.3 Molecular Docking of Target Compounds Against HDAC8

Compound **A3bn**’s binding mode showed a docking score of −8.79 kcal/mol towards HDAC8 (Table S4). Compound **A3bn** formed one metal ionic interaction and three hydrophobic π‐interactions with ZN403, Tyr306, Phe152, and Phe208. Moreover, compound **A3bn** interacted with Met274 and His180 by two hydrogen bonds with distances of 2.47 and 2.62 Å (Figure 2C). Compound **A5cn** exhibited an affinity score of −9.32 kcal/mol against HDAC8. It formed three hydrophobic π‐interactions with Tyr306, His180, and Met274 and formed a hydrogen bond and metal ion interactions with Met274 and ZN403 with a distance of 2.57 Å (Figure S15A).

The reference compounds (SAHA and trichostatin) exhibited an affinity score of −8.63 and −8.23 kcal/mol, respectively. SAHA formed three hydrophobic π‐interactions with Phe208, His180, and Phe152. Furthermore, it interacted with His142 and ZN403 by one hydrogen bond and metal ion interaction with a distance of 1.86 Å (Figure S15B). Although trichostatin formed six hydrophobic π‐interactions with Phe208, Met274, Ile34, Trp141, and Tyr306, additionally, one hydrogen bond and metal ion bond were observed with Ala32 and ZN401 with a distance of 2.60 Å (Figure S15C).

On the basis of molecular docking simulation studies, compound **A3bn** demonstrated a significant binding affinity for HDAC2, 6, and 8, suggesting its potential as HDACi. Compound **A3bn** exhibited higher stability and affinity within the binding pockets of the different HDACs. The pharmacophoric zinc‐binding group in compound **A3bn** can bind the zinc metal in the binding pocket of HDAC2, 6, and 8, leading to zinc ion chelation. This interaction was stabilized by different hydrogen bonds and π‐interactions.

### 3.2 Absorption, Distribution, Metabolism, Excretion, and Toxicity (ADMET) Studies

The in silico ADMET results of the newly synthesized compounds (Table S6 and Figure S16) provide important insights into their possible pharmacokinetic features. With the exception of compounds **A3an** and **A4**, which both showed low blood–brain barrier (BBB) permeability level, the majority of the compounds exhibited very low level of BBB permeability (Level 4) indicating limited ability to penetrate the central nervous system (CNS) [25]. SAHA was similar in its BBB permeability level to compounds **A3an** and **A4** (Level 3; low), whereas trichostatin showed medium BBB permeability level (Level 2).

The low BBB permeability of these new non‐hydroxamate benzoic acid derivatives is a beneficial safety feature, preventing potential neurotoxicity by limiting dangerous chemical entry into the brain. Although this low permeability makes the compounds safe for use, it also restricts their applicability, limiting them to treating non‐CNS cancers [25].

Drug solubility or the ability of a pharmaceutical to dissolve in a biological fluid such as water or gastrointestinal (GI) fluid is essential for that drug to pass through the GI tract membranes and into the bloodstream for efficient absorption and bioavailability [26]. The reference compound SAHA demonstrated optimal solubility, whereas compounds **A3bn**, **A4**, and **A5cn** had good levels of water solubility. However, the remaining compounds were expected to have low solubility levels. Low water solubility is a major challenge that might render a drug unworthy of advanced drug screening.

The majority of the new derivatives displayed good absorption, similar to the reference HDACi SAHA and trichostatin, indicating a high likelihood of systemic availability during treatment, which is a positive indicator for oral bioavailability. However, compounds **A4an** and **A4cn** showed poor and medium absorption, respectively. Poor absorption can result in decreased systemic bioavailability, requiring higher doses to achieve therapeutic levels [26].

Regarding CYP2D6 interaction, all compounds are expected to be non‐inhibitors, implying a lower probability of metabolic interactions with drugs metabolized by this enzyme.

All of the synthesized compounds were expected to cause hepatotoxicity. Additional research is necessary to determine which liver enzyme(s) bind(s) with the new compounds and cause hepatotoxicity.

Lastly, all of the examined compounds, including trichostatin but not SAHA, are shown to be bound to plasma proteins (such as albumin) more than 90% of the time. It should be noted that high plasma protein binding (PPB) limits the availability of drugs to interact with their tissue targets. Higher PPB lowers the concentration of free drug in plasma, the active form that can have pharmacologic effects, which in turn affects the distribution and elimination of the developed compound [27]. More work is needed in terms of drug design to eliminate their hepatotoxic effects and to optimize the compounds structures to increase their in vivo unbound drug concentration.

In conclusion, most of the newly synthesized compounds had favorable pharmacokinetic characteristics, including low levels of BBB permeability, good absorption and solubility, and non‐inhibition of CYP2D6. However, these compounds demonstrated minimal system bioavailability and hepatotoxicity. This requires structural modifications and further optimization to make the newly synthesized compounds promising candidates.

### 3.3 Molecular Similarity

The molecular similarity analysis aims to find the most similar compounds under examination to the reference compounds (such as SAHA and trichostatin) using Euclidean distance. The results were visualized in an output chart of structural similarity (Figure S17). On the basis of these descriptors, five compounds (**A3an**, **A3bn**, **A5cn**, **A5an**, and **A4**) were found to be similar to SAHA and trichostatin (Table S6).

### 3.4 Toxicity Studies

The safety evaluation of the selected compounds (Table S7) revealed an overall favorable toxicity profile. All experimental compounds, including **A3bn**, were categorized as nontoxic and noncarcinogenic, whereas SAHA was identified as the sole carcinogen and trichostatin as toxic. Specifically, **A3bn** demonstrated a carcinogenic potency (TD50) of 552.69 mg/kg body weight/day, which falls within the mid‐range of the tested compounds and is slightly lower than that of trichostatin (660.35 mg/kg body weight/day). Supporting parameters such as rat oral LD_50_ and chronic LOAEL further emphasized the safety of **A3bn**, with a low oral LD_50_ of 0.329 and a higher chronic LOAEL of 0.183, suggesting its suitability for long‐term application. In addition, all compounds were found to be nonirritants in both skin and ocular tests, further strengthening their safety profile. Taken together, these findings highlight **A3bn** as a promising candidate with a reassuring safety margin, though comprehensive validation through in vivo experiments and clinical trials remains necessary to confirm its therapeutic potential.

### 3.5 Molecular Orbital Analysis

We performed an extensive density functional theory (DFT) analysis for the new compounds comparing them to two standards HDACi (SAHA and trichostatin) (Table S8). The computed total energies indicate the inherent stability of the molecules. Among the studied compounds, compounds **A5cn**, **A3bn**, and **A5bn** exhibited the most negative total energy, reflecting their high stability. By contrast, the reference compounds trichostatin and SAHA showed significantly less negative total energy. The binding energy reflects the strength of molecular interaction between two compounds, that is, a new drug candidate and its target. The reference compound trichostatin exhibited the most favorable binding energy (−8.373 kcal/mol), indicating its strong interaction potential to histones. Among the new compounds, compound **A5cn** showed the most favorable binding energy (−7.866 kcal/mol), followed closely by **A5an**, **A5bn**, and **A3bn**. These results suggest that these molecules could serve as promising candidates for further optimization.

Frontier molecular orbital (FMO) energies are critical indicators of molecular reactivity. The HOMO energy represents the ability to donate electrons, whereas the LUMO energy reflects the ability to accept electrons (Figure S18). Among the tested compounds, compound **A4an** had the lowest HOMO energy, indicating strong electron‐donating potential. Additionally, this compound (**A4an**) also exhibited the most negative LUMO energy, signifying enhanced electron‐accepting capability. The band gap energy (HOMO‐LUMO gap) is an important parameter for assessing electronic properties and reactivity. Compound **A4an** displayed the smallest band gap energy, implying high electronic excitation and reactivity potential. In contrast, SAHA exhibited the largest band gap, indicating lower reactivity and electronic transition capability (Table S8). The dipole moment is a measure of molecular polarity, which affects solubility and interaction with polar environments. Trichostatin (4.42 D) and compound **A3bn** (4.81 D) exhibited the highest dipole magnitudes, suggesting better solubility and interaction potential in polar systems. In contrast, SAHA had the lowest dipole moment (0.79 D), indicating lower polarity (Table S8).

Although trichostatin exhibited superior binding energy, dipole moment, and electronic properties, several test compounds, such as **A5an–5cn** and **A3bn**, demonstrated comparable or favorable binding energies. Moreover, compounds **A4an**, **A3bn**, and **A4dn** displayed enhanced reactivity due to their smaller band gaps, making them potential candidates for further investigation. Further experimental studies should validate these theoretical findings and explore their interactions in biological systems.

### 3.6 Molecular Dynamic (MD) Simulation Study

MD simulations were conducted for 100 ns to study the molecular stability of compound **A3bn** within the active site of HDAC2. The obtained root mean square deviation (RMSD) for the complex and the ligand concerning their original position within the active site was reported and analyzed. Frontier compounds interactions were also analyzed and evaluated in detail. Finally, the molecular mechanics with a generalized Born and surface area solvation (MM–GBSA) free binding energy was estimated for the tested complex during the simulation trajectories.

#### 3.6.1 Protein and Ligand RMSD and RMSF Analysis

The conformational stability of HDAC2 protein structure was monitored through the C𝛼 curve of the protein concerning their initial position. As shown in Figure S19, compound **A3bn** showed high stability within the binding pocket of HDAC2 with an RMSD value of 2.2 Å, which is an acceptable value below 3.00 Å. Compound **A3bn** showed some minor fluctuations between 0 and 30 ns and then showed stability until the end of the simulation period. Additionally, the protein structure of HDAC2 showed notable stability over the simulation time and fluctuated within 2.00 Å. Moreover, it showed some minor fluctuations at the 20–30 amino acid area, which indicates that few conformational changes occurred and that the changes did not affect the ligand binding inside the active site.

#### 3.6.2 Protein Ligand Interactions Analysis

Compound **A3bn** showed notable binding mode within the active pocket of HDAC2, and their interactions are discussed in detail. Compound **A3bn** formed H‐bond interactions with the following residues: Tyr29, Arg3, His145, His146, Arg27, Gly305, and Tyr308 (Figure S20). Compound **A3bn** formed water‐bridged H‐bonds with residues Tyr29 and Arg275. Furthermore, compound **A3bn** was able to form hydrophobic interactions with residues Leu144, Phe155, Phe210, Leu276, and Tyr308 and was able to form ionic interactions with the zinc ion in the active site, which acts as a linker between the tested ligand and the following target amino acids: Asp181, His1883, Asp269, and Tyr308 (Figure S20).

The number of interactions at each frame of compound **A3bn**/HDAC2 complex, where dark color indicates more interactions, is shown as a heat map (Figure S21). From the heat map figure, it was observed that the highest number of conformations of HDAC2 protein formed up to fifteen bonds, and the most interacted amino acids of HDAC2 with compound **A3bn** were Tyr29, Arg39, His145, His146, Asp181, His183, Asp269, Gly305, and Tyr308.

#### 3.6.3 Molecular Mechanic Poisson–Boltzmann Surface Area (MM–GBSA) for Compound A3bn

MM–GBSA was carried out to calculate both the ligand binding strain and free energies for the docked ligand over the 100 ns simulation period. The results obtained are described in Table S9. Compound **A3bn**/HDAC2 showed change in ∆*G* from the starting period until the end of the simulation period with the energy at 0 ns slightly higher than that at the 100 ns. However, the interaction is still exothermic indicating that the complex is stable during the simulation process.

## 4 Biological Evaluations

### 4.1 Antiproliferative Activities of the New 3‐Chloro‐4‐((2‐Ethoxy‐2‐Oxoethyl)Amino)Benzoic Acid Derivatives

The cytotoxic effects of the new 3‐chloro‐4‐((2‐ethoxy‐2‐oxoethyl)amino)benzoic acid derivatives were determined using the MTT test, and their IC_50_ concentrations were compared to those of the reference HDACi SAHA (Table 1). The findings show that the newly developed compounds were more cytotoxic against MCF‐7 breast cancer cells (average IC_50_ = 65.5 µM) and HepG2 liver carcinoma cells (average IC_50_ = 67.1 µM), but less effective against K562 leukemia cells (average IC_50_ = 86.6 µM) (Table 1). The most cytotoxic compound was the benzohydrazone derivative **A3bn**, whose IC_50_ concentrations against MCF‐7 and K562 cells were comparable to those of SAHA. Additionally, the hydrazine‐1‐carbothiomide derivatives (**A5an–5cn**) had comparable IC_50_ values across all three cell lines studied. The least cytotoxic compounds were the benzohydrazone derivative **A3an** and the 1,3,4‐oxadiazole derivatives **A4an–4dn**.

**TABLE 1:** Antiproliferation activities of the new compounds assayed by the MTT in vitro cytotoxicity assay.

Compounds	In vitro cytotoxicity IC_50_ (µM)			
	HepG2	MCF‐7	K562	MCF‐10A
**A3an**	36.7 ± 3.1	72.4 ± 6.7	90.1 ± 7.6	
**A3bn**	74.3 ± 8.3	39.2 ± 2.8	42.0 ± 5.3	314.7 ± 35.3
**A4**	97.3 ± 7.4	83.3 ± 5.9	117.6 ± 10.4	
**A4an**	76.1 ± 6.6	140.2 ± 13.8	123.7 ± 11.1	
**A4bn**	58.3 ± 4.5	50.9 ± 4.9	80.4 ± 8.1	
**A4cn**	90.5 ± 8.1	62.2 ± 7.4	132.5 ± 12.9	
**A4dn**	48.3 ± 3.1	40.1 ± 4.0	110.1 ± 9.6	
**A5an**	34.1 ± 2.1	60.5 ± 6.9	69.5 ± 6.1	
**A5bn**	97.6 ± 9.9	55.5 ± 3.7	36.2 ± 4.1	
**A5cn**	58.0 ± 4.1	50.3 ± 4.8	64.1 ± 5.9	
**SAHA**	13.8 ± 5.1	20.3 ± 2.2	36.1 ± 9.7	269.5 ± 29.6

*Note*: Data are presented as mean IC_50_ (µM) ± SD of three experiments performed in triplicates.

To evaluate cancer selectivity of compound **A3bn**, its cytotoxic effects were also investigated against normal human breast (MCF‐10A) cells (Table 1). The selectivity index (SI) was determined by dividing the IC_50_ against the normal cell line MCF‐10A by the IC_50_ against the corresponding cancer cell line (MCF‐7). Higher anticancer selectivity is indicated by higher SI values, and compounds with SI values larger than 3 are regarded to be very selective [28]. The SI value of compound **A3bn** was 8 in MCF7 cells indicating remarkable anticancer selectivity.

### 4.2 Inhibition of HDAC Activity

To validate the results of the molecular docking studies presented in the current work (Figures S13–S15 and Tables S2–S4), the inhibition of the zinc‐dependent class I and II HDAC activity by the new derivatives was evaluated using the HDAC‐Glo I/II assay. Table 2 indicates that compound **A3bn** (a benzohydrazone derivative) was the most potent in inhibiting HDAC enzymes. However, the other two groups of derivatives, the 1,3,4‐oxadiazole (**A4an–4dn**) and the hydrazine‐1‐carbothioamide (**A5an–5cn**) derivatives, were not effective. On the basis of docking scores, MTT assay results, and HDAC inhibition results, compound **A3bn** was chosen for further biological investigation along with MCF‐7 breast cancer cells.

**TABLE 2:** Inhibition of HDAC enzyme activity by the new derivatives shown as EC_50_.

Compounds	EC_50_ (nM)
**A3an**	88.5 ± 8.1
**A3bn**	3.1 ± 0.3
**A4**	25.8 ± 2.7
**A4an**	77.3 ± 5.5
**A4bn**	89.2 ± 6.9
**A4cn**	91.2 ± 10.0
**A4dn**	67.9 ± 4.8
**A5an**	65.8 ± 5.1
**A5bn**	76.2 ± 7.9
**A5cn**	66.1 ± 8.0
**SAHA**	0.3 ± 0.07
**Trichostatin**	0.02 ± 0.002

*Note*: Data are shown as mean ± SD of two experiments performed in triplicates.

**FIGURE 3:**
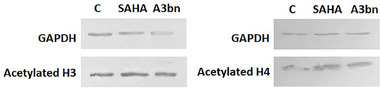
Representative western blot analysis of acetylated histone H3 and H4 in SAHA‐ and **A3bn**‐treated MCF‐7 breast cancer cells as compared to control (C) untreated cells.

To confirm the inhibition of HDAC activity by compound **A3bn**, MCF‐7 cells were treated with the IC_50_ concentrations of **A3bn** and SAHA, and the acetylation of histones H3 and H4 was compared to that of untreated control cells by immunoblotting (Figure 3). The results show that compound **A3bn** inhibited the deacetylation of H3 and H4 as evidenced by distinct bands of acetylated proteins in both the control and treated lanes.

### 4.3 Compound A3bn Induces Apoptosis by Activating Caspase 8

The readily available HDACi SAHA has been shown to induce apoptosis in a range of cancer cell lines [29]. When MCF‐7 breast cancer cells were treated with **A3bn** at the IC_50_ concentration for 72 h, the percentage of apoptotic cells increased by about 150% (12.7%) in comparison to the control untreated cells (5.2%). Similarly, when MCF‐7 cells were treated with SAHA (IC_50_; 72 h), the percentage increased by 250% (18.4%).

**FIGURE 4:**
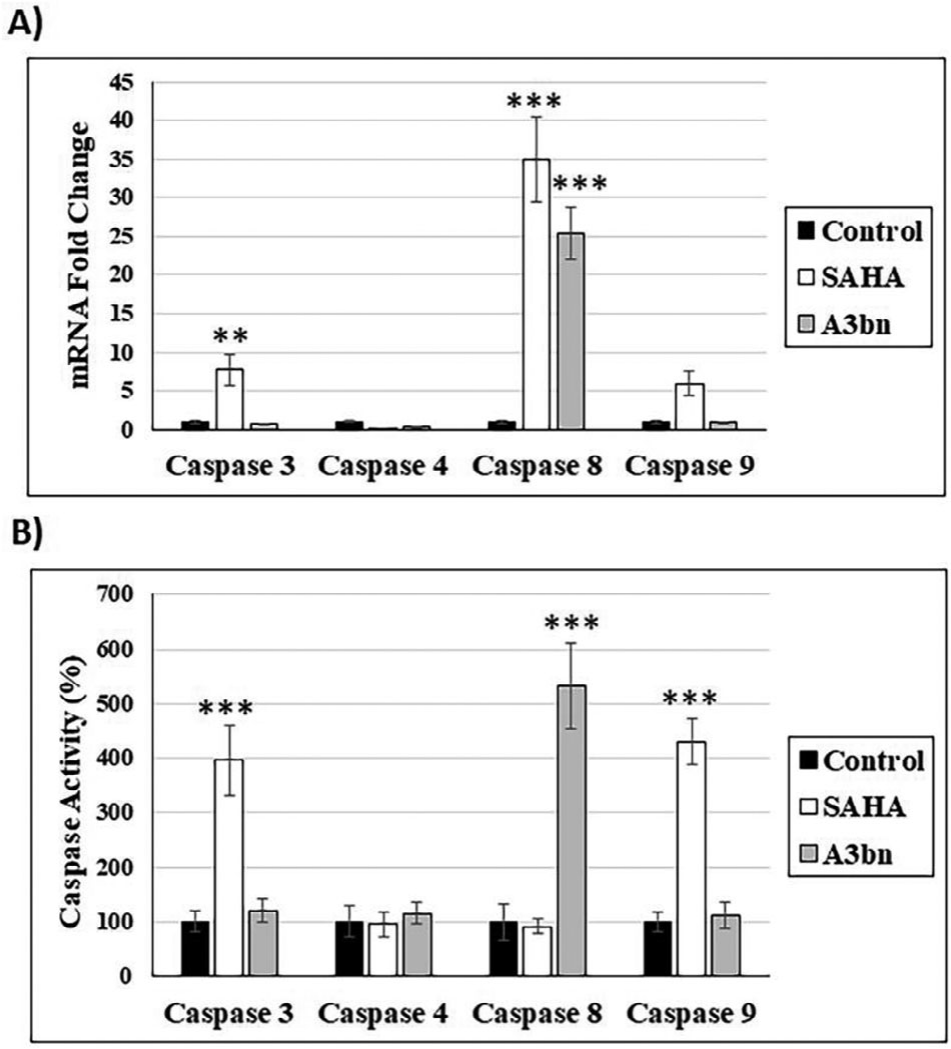
Analysis of the expression and activity of the different caspases in control and compound **A3bn**‐treated (IC_50_; 72 h) MCF‐7 breast cancer cells. (A) mRNA fold change of the different caspases in compound **A3bn**‐treated MCF‐7 cells normalized to β‐actin and compared to control MCF‐7 cells (set as 1.0 a.u.). (B) Quantification of the activity of the different caspases (caspase 3, 4, 8, and 9) in compound **A3b**n‐treated cells as compared to control cells (set as 100% a.u.). *p < 0.01, ***p < 0.001 versus control cells. Data are presented as the mean of 2 experiments performed in triplicates ± SD.

The apoptotic pathway induced by **A3bn** and SAHA was also investigated (Figure 4). Compound **A3bn** increased the expression of *caspase 8* gene, whereas SAHA increased the expression of *caspase 3*, *8*, and *9* genes (Figure 4A). At the protein level, SAHA treatment activated caspase 3 and 9 and, thus, the intrinsic apoptotic pathway, whereas compound **A3bn** activated the extrinsic pathway by activating caspase 8 (Figure 4B).

### 4.4 Structure–Activity Relationships (SAR)

**FIGURE 5:**
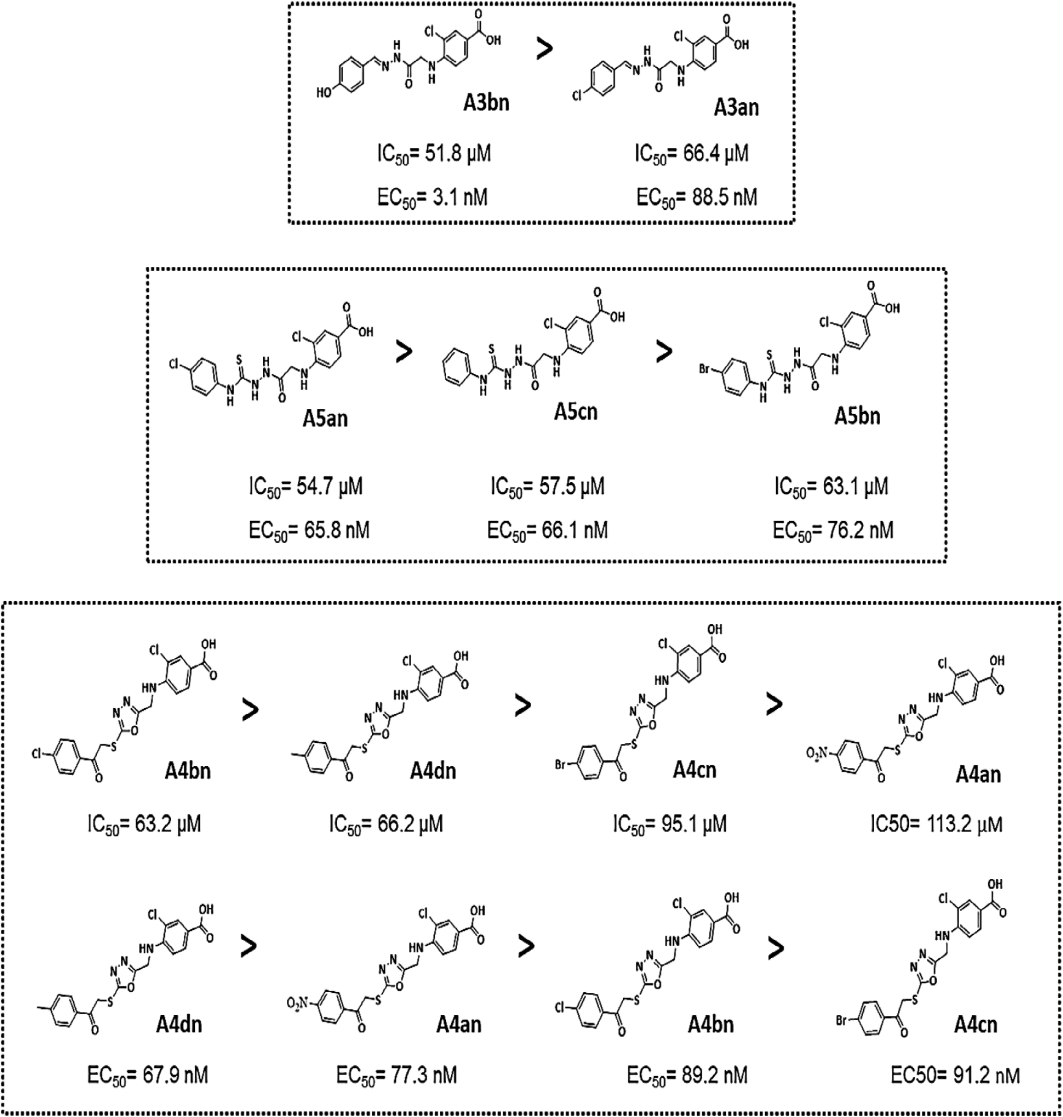
Structure–activity relationships of the new benzoic acid derivatives.

The SARs of the new compounds as potential HDACis were examined by comparing the in vitro biological data with the in silico findings (Figure 5). Regarding the hydrazone derivatives **A3an** and **A3bn**, compound **A3bn**, which contains a terminal hydrophobic tail substituted with an electron‐donating group (OH), was more active and more cytotoxic than compound **A3an** with an electron‐withdrawing group (Cl). The terminal hydroxyl group in compound **A3bn** was able to form an additional hydrogen bond with residue Asp104 within HDAC target pocket enhancing the binding affinity, whereas the second hydrogen bond was formed between hydrazone and residue Gly154. Additionally, the hydrazone hydrophilic linker between the cap and the classical zinc‐binding group (carboxyl moiety) contains multiple hydrophilic centers that are capable of coordinating zinc in narrow pockets inside the active site of HDAC enzymes, where the zinc atom is positioned at the center of the target pocket. This structural feature likely contributes to the superior activity of compound **A3bn** against a wide range of HDACs isoforms (HDAC2, 6, and 8), as it allows for effective interaction with the zinc‐dependent active sites of various HDAC isoforms (Table S2–S4).

In terms of the activities of the derivatives with oxadiazole ring (**A4–A4dn**), creating the phenacyl bromide derivatives (**A4an**–**4dn**) from compound **A4** diminished their HDAC inhibition effect (Table 2) which in turn caused them to be noncytotoxic. Introduction of a halogen atom at the para position (**A4an–4cn**) decreased HDAC inhibitory activity, as compared to compound **A4dn** (4‐methyl position). The introduction of an electron‐donating group (CH_3_) at the 4‐position of phenyl group (**A4dn**) exhibited a higher activity than electron‐withdrawing groups at position 4 of the phenyl ring (**A4an**, **A4bn**, and **A4cn**) (NO_2_, Cl, and Br groups), respectively.

Finally, the enzyme inhibitory activities of the derivatives with para‐substituted phenyl isothiocyanates (**A5an–5cn**) were as follows: compound **A5an** containing chloro substitution at the 4‐position showed the highest inhibitory activity against HDACs, whereas replacing the chloro group with a bromo group (compound **A5bn**) caused a decrease in HDAC inhibitory activity. The cytotoxicity of these compounds mirrored that of their HDAC inhibitory activities: **A5an** > **A5cn** > **A5bn**.

## 5 Conclusion

ATR‐FTIR, ^1^HNMR, ^13^CNMR, and mass spectra data (Figures S1–S12) demonstrated that the new 3‐chloro‐4‐((2‐ethoxy‐2‐oxoethyl) amino)benzoic acid derivatives were successfully prepared. According to the results of molecular docking, compound **A3bn** exhibited high affinity for HDAC2, HDAC6, and HDAC8, three zinc‐dependent class I/II HDACs. Molecular similarity study revealed that compound **A3bn** was similar to trichostatin and SAHA (reference HDACi). Compound **A3bn** was shown to be safe in comparison to SAHA (single carcinogen) and trichostatin (toxic). Compound **A3bn** exhibited favorable binding energy and high stability, and it had the greatest dipole magnitude, indicating superior solubility in polar systems and interaction potential.

Among the new derivatives, compound **A3bn** was found to be the most cytotoxic, and its IC_50_ concentrations against MCF‐7 and K562 cancer cells were comparable to those of SAHA. Compound **A3bn** was the most effective compound at inhibiting HDAC enzymes. Compound **A3bn** increased the percentage of apoptotic cells by 150%, in comparison to SAHA, which increased it by 250%. Compound **A3bn** activated the extrinsic apoptotic pathway by activating caspase 8, whereas SAHA activated caspases 3 and 9 and, thus, the intrinsic apoptotic pathway.

Compound **A3bn** hydrazone hydrophilic linker between the cap and the classical zinc‐binding group (carboxyl moiety) contains multiple hydrophilic centers that are capable of coordinating zinc in narrow pockets, such as in HDAC2, where the zinc atom is positioned at the center of the target pocket. This structural feature likely contributes to the superior activity of compound **A3bn** against a wide range of HDACs isoforms (HDAC2, 6, and 8), as it allows for effective interaction with the zinc‐dependent active sites of various HDAC isoforms.

The success rates of currently approved HDACis vary across different cancer types. Vorinostat has a response rate of 30% in cutaneous T‐cell lymphoma (CTCL) [30]. Romidepsin exhibits an overall response rate of 34% in CTCL and 25% in peripheral T‐cell lymphoma (PTCL) [31]. Belinostat, a hydroxamic acid compound approved for PTCL, shows an overall response rate of 26% [32]. Panobinostat, used for the treatment of multiple myeloma, achieves a response rate of 27% [33]. Considering these response rates and building on our results, compound **A3bn** demonstrated comparable effectiveness and could serve as a promising candidate for further optimization. Further refinements will focus on enhancing the compound's efficacy at lower concentrations.

## 6 Experimental Procedures

### 6.1 Materials and Methods

All chemicals and reagents were of analytical grade and were utilized without further purification. 4‐Amino‐3‐chlorobenzoic acid was purchased from HyperChem (China). The uncorrected melting points were ascertained utilizing the MP‐200 Series Stuart Digital Melting Point Apparatus (Cole‐Parmer). Spectroscopic analysis of the IR spectra was performed using Shimadzu ATR‐FTIR Spectrophotometer (ύ, cm^−1^). The proton (^1^H) nuclear magnetic resonance (^1^HNMR) and carbon (^13^CNMR) spectra of the synthesized compounds were obtained using BRUKER instrument operating at 300 and 75 MHz, respectively. The internal standard employed was tetramethyl silane (TMS), with the chemical shift denoted as *δ* = ppm. The solvent utilized was DMSO*_d_*, and the coupling constants (J) are reported in hertz (Hz). Splitting patterns are denoted as follows: singlet (s), doublet (d), multiplet (m), triplet (t), quartet (q), and doublet of doublets (dd). The *m/z* mass spectra for all produced compounds were obtained using liquid chromatography mass spectrometry with electrospray ionization (ESI) using a triple‐quadrupole instrument, achieving a resolution of 0.1 *m/z*. The investigation revealed a maximum variation of ±0.4 from the calculated theoretical values.

The progress of the reaction was monitored by thin layer chromatography (TLC) using (*n*‐hexane/ethyl acetate 8:2), (ethanol/toluene 8:2), (*n*‐hexane/ethyl acetate 6:4), and (*n*‐hexane/ethyl acetate 3:7) as a mobile phase and UV light (254 nm) to visualize the resulting spots. Schemes 1, 2, 3, 4 show the route of synthesis, whereas the description, molecular formula, Mwt, m.p, and *R_f_* value of the synthesized compounds are summarized in Table S1.

### 6.2 Chemical Synthesis

#### 6.2.1 Synthesis of 3‐Chloro‐4‐((2‐Ethoxy‐2‐Oxoethyl)Amino)Benzoic Acid) **A1**) [34, 35]

4‐Amino‐3‐chlorobenzoic acid (0.01 mol, 1.71 g) with K_2_CO_3_ (0.02 mol, 2.76 g) in dry DMF (20 mL) was stirred for 30 min. Ethyl bromo acetate (0.015 mol, 2.5 mL) was then added drop by drop, refluxed for 3 h, and at the end of the reaction, the mixture was poured into ice water. The precipitate obtained was washed several times with water and recrystallized with EtOH 70%.

Off‐white powder, yield (75%), mp = (100–103)°C, *R_f_* = 0.50 (*n*‐hexane/ethyl acetate 8:2).

ATR‐FTIR (ύ, cm^−1^): 3367 (NH) str, 3209 (OH) str of (COOH), 2997 str of (CH_2_), 1701 (C═O) acid str, 1624 (C═O) ester str, 1593, 1558, 1512 Ar (C═C) str, 1384 (CH_3_) bend, 763 (C─Cl) str.

^1^HNMR (300 MHz, DMSO*_d6_*, *δ*, ppm): 7.77 (s, 1H, ArH), 7.65 (d, *J* = 9.0 Hz, 1H, ArH), 6.85 (d, *J* = 9.0 Hz, 1H, ArH), 6.36 (*br*s, 2H, NH_2_ **^+^**‐zwitter ion), 4.8 (s, 2H, NHCH_2_), 4.15 (q, *J* = 6.0, 2H, OCH_2_), 1.20 (t, *J* = 6.0 Hz, 3H, CH_3_).

^13^CNMR (75 MHz, DMSO*_d6_*, *δ*, ppm): 167.94 (C═O), 164.35 (COOH), 149.64, 131.08, 130.72, 129.68, 116.00, 114.25 (Ar─C), 60.96 (CH_2_), 60.74 (CH_2_─NH), 13.98 (CH_3_).

MS (ESI) *m/z*: Calcd. For C_11_H_12_ClNO_4_ [M + 1]**^+^**: 258.05, found 258.20.

#### 6.2.2 Synthesis of 3‐Chloro‐4‐((2‐Hydrazinyl‐2‐Oxoethyl)Amino)Benzoic Acid (**A2**) [36, 37]

3‐Chloro‐4‐((2‐ethoxy‐2‐oxoethyl)amino)benzoic acid (**A1**) (0.000388 mol, 0.1 g) and hydrazine hydrate 80% (an excess amount of 0.0027 mol, 0.087 g, 2 mL) were added to 50 mL of abs EtOH in a 100 mL round bottom flask, and the mixture was refluxed at 80°C overnight. At the end of the reflux time, the solvent was evaporated to dryness, and the precipitate was washed with D.W., filtered off, allowed to dry, and recrystallized with methanol.

White to orange powder, yield (65%), mp = (105–106)°C, *R_f_* = 0.50 (ethanol/toluene 8:2).

ATR‐FTIR (ύ, cm^−1^): 3317, 3290 *asym* and *sym* (NH) str of *prim*. amine, 3051 (OH) str of (COOH), 2958, 2920 *asym* and *sym* str of (CH_2_), 1701 (C═O) acid str, 1666 (C═O) amide str, 1593, 1539, 1508 Ar (C═C) str, 759 (C─Cl) str.

^1^HNMR (300 MHz, DMSO*_d_*_6_, *δ*, ppm): 9.44 (s, 1H, NH), 7.53 (d, *J* = 8.4 Hz, 1H, ArH), 7.71 (d, *J* = 1.27 Hz, 1H, ArH), 6.75 (d, *J* = 8.4 Hz, 1H, ArH), 5.86 (*br*s, 2H, NH_2_), 4.34 (s, 2H, CH_2_).

^13^CNMR (75 MHz, DMSO*_d_*_6_, *δ*, ppm): 165.09 (COOH and CONHNH), 147.23, 128.58, 128.17, 126.91, 121.19, 116.09, 114.17 (Ar─C), 40.01 (CH_2_).

MS (ESI) *m/z*: Calcd. for C_9_H_10_ClN_3_O_3_ [M + 2]**^+^**: 245.04, found 245.20.

#### 6.2.3 Synthesis of 3‐Chloro‐4‐(((5‐Thioxo‐4,5‐Dihydro‐1,3,4‐Oxadiazol‐2yl)Methyl)Amino)Benzoic Acid (**A4**) [38, 39, 40]

3‐Chloro‐4‐((2‐hydrazinyl‐2‐oxoethyl) amino)benzoic acid (**A2**) (0.00077 mol, 0.2 g) was dissolved in abs EtOH (20 mL) and cooled to 20°C on ice bath. Afterward, KOH (0.00077 mol, 0.0435 g) was dissolved in EtOH (10 mL), added to the mixture, and the mixture was stirred for 15 min. Carbon disulfide (CS_2_) (0.00077 mol, 0.0586 g, 0.046 mL) was added slowly, and a yellow precipitate appeared. The reaction mixture was refluxed for 12 h, and the solvent was reduced. The produced solid was dissolved in 25 mL of cold D.W. and acidified with 1% HCl until white crystals of the product were obtained and recrystallized with EtOH 70%.

White to orange powder, yield (60%), mp = (251–253)°C, *R_f_* = 0.50 (*n*‐hexane/ethyl acetate 8:2).

ATR‐FTIR (ύ, cm^−1^): 3336 (NH) str, 3070 (OH) str of COOH, 2943, 2765 *asym* and *sym* str of (CH_2_), 1620, 1600, 1554 Ar (C═C) str, 1354 (CH_2_) bend, 1188 (C═S) str, 721 (C–Cl) str.

^1^HNMR (300 MHz, DMSO*_d6_*, *δ*, ppm): 14.46 (*brs*, 1H, NHCS), 7.64 (d, *J* = 1.47 Hz, 1H, ArH), 7.53 (dd, *J* = 8.42, 1.83 Hz, 1H, ArH), 6.90 (d, *J* = 8.42 Hz, 1H, ArH), 6.26 (s, 2H, CH_2_).

^13^CNMR (75 MHz, DMSO*_d_*_6_, *δ*, ppm): 176.74 (C═S), 160.29 (C═O), 148.34 (ArC–NH), 127.38, 125.93, 125.66, 116.74, 115.03, 109.83 (Ar–C).

MS (ESI) *m/z*: Calcd. for C_10_H_8_ClN_3_O_3_S [M + 1]**^+^**: 285.997, found 286.30.

#### 6.2.4 General Method for Synthesis of Benzohydrazone Derivatives (Compounds **A3an–3bn**) [41, 42, 43]

Compound (**A2**) (0.000385 mol, 0.1 g) in 20 mL abs EtOH and (0.00041 mol) of each of the following substituted aromatic aldehydes: 4‐hydroxybenzaldehyde (0.05 g) and 4‐chlorobenzaldehyde (0.057 g) were dissolved separately in 5 mL of the same solvent containing few drops of glacial acetic acid (GAA) and stirred for 15 min to give a clear mixture. The mixture was refluxed for 6 h, and at the end of the reflux time, the solvent was evaporated to dryness, and the precipitate was then washed with D.W., filtered off, and recrystallized with EtOH 70%.

#### 6.2.5 3‐Chloro‐4‐((2‐(2‐(4‐Chlorobenzylidene) Hydrazinyl)‐2‐Oxoethyl)Amino)Benzoic Acid (**A3an**)

White powder, yield (60%), mp = (166–168)°C, *R_f_* = 0.55 (*n*‐hexane/ethyl acetate 3:7).

ATR‐FTIR (ύ, cm^−1^): 3398 (OH) str of (COOH), 3336 (NH) str of amide, 3224 (NH) str of amine, 1674 (C═O) amide str, 1600 (C═N) str, 1585, 1546, 1508 Ar (C═C) str, 725 (C–Cl) str.

^1^HNMR (300 MHz, DMSO*_d6_*, *δ*, ppm): 11.46 (*brs*, 1H, NH), 8.38 (s, 1H, CH═N), 7.85 (s, 1H, ArH), 7.74–7.65 (m, 3H, ArH), 7.52–7.49 (m, 2H, ArH), 6.85 (d, *J* = 9.0 Hz, 1H, ArH), 6.05 (s, 2H, CH_2_).

^13^CNMR (75 MHz, DMSO*_d_*_6_, *δ*, ppm): 168.36 (C═O), 164.66 (COOH), 162.18, 148.03 (C═N), 145.76, 145.45, 134.34, 133.24, 128.60, 120.43, 116.17, 116.04, 114.28, 114.17 (Ar–C).

MS (ESI) *m/z*: Calcd. for C_16_H_13_Cl_2_N_3_O_3_ [M]**^+^**: 365.03, found 365.30.

#### 6.2.6 3‐Chloro‐4‐((2‐(2‐(4‐Hydroxybenzylidene) Hydrazinyl)‐2‐Oxoethyl) Amino)Benzoic Acid (**A3bn**)

White powder, yield (60%), mp = (210–212)°C, *R_f_* = 0.50 (*n*‐hexane/ethyl acetate 3:7).

ATR‐FTIR (ύ, cm^−1^): 3464 (NH) str of amide, 3305 (NH) str of amine, 3143 (OH) str of (COOH), 1681 (C═O) amide str, 1593 (C═N) str, 1550, 1492, 1435 Ar (C═C) str, 752 (C–Cl) str.

^1^HNMR (300 MHz, DMSO*_d6_*, *δ*, ppm): 11.37 (*br*s, 1H, NH), 9.89 (*br*s, 1H, OH), 8.30 (s, 1H, CH═N) 7.83–7.52 (m, 4H, ArH), 6.84–6.82 (m, 3H, ArH), 6.01 (s, 2H, CH_2_).

^13^CNMR (75 MHz, DMSO*_d_*_6_, *δ*, ppm): 161.78 (C═O), 159.16 (COOH), 147.79 (C═N), 147.36, 129.19, 128.80, 128.70, 127.72, 125.43, 120.95, 116.13, 115.69, 114.24 (Ar─C).

MS (ESI) *m/z*: Calcd. for C_16_H_14_ClN_3_O_4_ [M]**^+^**: 348.06, found 348.30.

#### 6.2.7 General Method for Synthesis of 1,3,4‐Oxadiazole Derivatives (Compounds **A4an–4dn**) [44, 45, 46]

To a suspension mixture of compound (**A4**) (0.00035 mol, 0.1 g) in 10 mL of abs EtOH, triethylamine (0.00035 mol, 0.035 mL) was added and stirred for 15 min in a 100 mL round bottom flask. The color of the suspended mixture changed to yellow, then separately 2‐bromo‐4′‐methylacetophenone (0.00035 mol, 0.094 g), 2,4′‐dibromoacetophenone (0.00035 mol, 0.097 g), 2‐bromo‐4′‐chloroacetophenone (0.00035 mol, 0.081 g), and 2‐bromo‐4‐nitroacetophenone (0.00035 mol, 0.085 g) were added carefully and stirred for 2 h at RT. Later, the formed precipitate was filtered off and recrystallized with EtOH 70%.

#### 6.2.8 3‐Chloro‐4‐(((5‐((2‐(4‐Nitrophenyl)‐2‐Oxoethyl)Thio)‐1,3,4‐Oxadiazol‐2yl)Methyl)Amino)Benzoic Acid (**A4an**)

Yellow powder, mp = (199–201)°C, yield (80%), *R_f_* = 0.61 (*n*‐hexane/ethyl acetate 6:4).

ATR‐FTIR (ύ, cm^−1^): 3332 (NH) str of amine, 3209 (OH) str of (COOH), 2954, 2916 *asym* and *sym* str of (CH_2_), 1678 (C═O) ketone str, 1604, 1523, 1473 Ar (C═C) str, 1408 (CH_2_) bend, 763 (C─Cl) str.

^1^HNMR (300 MHz, DMSO*_d_*_6_, *δ*, ppm): 8.41 (d, *J* = 9.0 Hz, 2H, ArH), 8.32 (d, *J* = 9.0 Hz, 2H, ArH), 7.97 (s, 1H, NH), 7.68–7.60 (m, 2H, ArH), 6.90 (d, *J* = 9.0 Hz, 1H, ArH), 6.24 (s, 2H, NHCH_2_), 5.21 (s, 2H, S–CH_2_).

^13^CNMR (75 MHz, DMSO*_d6_*, *δ*, ppm): 192.30 (C═O), 164.83 (COOH), 161.35 (C═N‐oxadiaz.), 150.26, 148.09, 139.70, 130.09, 126.83, 126.43, 124.08, 123.72, 116.72, 115.20, 110.49 (Ar─C), 40.49 (CH_2_─S).

MS (ESI) *m/z*: Calcd. for C_18_H_13_ClN_4_O_6_S [M + 2]**^+^**: 450.02, found 450.20.

#### 6.2.9 3‐Chloro‐4‐(((5‐((2‐(4‐Chlorophenyl)‐2‐Oxoethyl)Thio)‐1,3,4‐Oxadiazol‐2‐yl)Methyl)Amino)Benzoic Acid (**A4bn**)

White powder, mp = (166–168)°C, yield (70%), *R_f_* = 0.57 (*n*‐hexane/ethyl acetate 6:4).

ATR‐FTIR (ύ, cm^−1^): 3336 (NH) str of amine, 3224 (OH) str of (COOH), 2908 str of (CH_2_), 1674 (C═O) ketone str, 1604, 1585, 1508 Ar (C═C) str, 1477 (CH_2_) bend, 771 (C–Cl) str.

^1^HNMR (300 MHz, DMSO*_d_*_6_, *δ*, ppm): 8.08 (d, *J* = 9.0 Hz, 2H, ArH), 7.68–7.58 (m, 4H, ArH), 6.89 (d, *J* = 6.0 Hz, 1H, ArH), 6.19 (s, 2H, NHCH_2_), 5.11 (s, 2H, S–CH_2_).

^13^CNMR (75 MHz, DMSO*_d_*_6_, *δ*, ppm): 192.01 (C═O), 164.76 (COOH), 161.52 (C═N‐oxadiaz), 148.09 (ArC–NH), 138.91, 133.75, 130.60, 129.50, 128.59, 127.67, 126.76, 116.73, 115.22, 110.52 (Ar–C), 40.31 (CH_2_–S).

MS (ESI) *m/z*: Calcd. for C_18_H_13_Cl_2_N_3_O_4_S [M + 2]**^+^**: 439.00, found 439.30.

#### 6.2.10 4‐(((5‐((2‐(4‐Bromophenyl)‐2‐Oxoethyl)Thio)‐1,3,4‐Oxadiazol‐2‐yl)Methyl)Amino)‐3‐Chlorobenzoic Acid (**A4cn**)

White powder, mp = (145–148)°C, yield (80%), *R_f_* = 0.55 (*n*‐hexane/ethyl acetate 6:4).

FTIR (ύ, cm^−1^): 3371 (NH) str of amine, 3228 (OH) str of (COOH), 2912 str of (CH_2_), 1670 (C═O) ketone str, 1604, 1581, 1504Ar (C═C) str, 1477 (CH_2_) bend, 763 (C─Cl) str, 671 (C─Br) str.

^1^HNMR (300 MHz, DMSO*_d_*_6_, *δ*, ppm): 7.99 (d, *J* = 8.90 Hz, 2H, ArH), 7.81 (d, *J* = 9.0 Hz, 2H, ArH), 7.67 (d, *J* = 2.2 Hz, 1H, ArH), 7.58 (d, *J* = 8.2 Hz, 1H, ArH), 6.89 (d, *J* = 8.0 Hz, 1H, ArH), 6.19 (s, 2H, NHCH_2_), 5.11 (s, 2H, SCH_2_).

^13^CNMR (75 MHz, DMSO*_d_*_6_, *δ*, ppm): 192.87 (C═O), 165.44 (COOH), 162.19 (C═N), 148.74, 134.72, 132.62, 131.10, 128.83, 127.91, 127.00, 117.42, 115.81, 111.21 (Ar–C), 40.86 (CH_2_–NH), 29.67 (CH_2_–S).

MS (ESI) *m/z*: Calcd. for C_18_H_13_BrClN_3_O_4_S [M + 2]**^+^**: 482.94, found 483.30.

#### 6.2.11 3‐Chloro‐4‐(((5‐((2‐*oxo*‐2‐(*p*‐Tolyl)Ethyl)Thio)‐1,3,4‐Oxadiazol‐2‐yl)Methyl)Amino)Benzoic Acid (**A4dn**)

White powder, mp = (176–178)°C, yield (75%), *R_f_* = 0.50 (*n*‐hexane/ethyl acetate 6:4).

FTIR (ύ, cm^−1^): 3336 (NH) str of amine, 3228 (OH) str of (COOH), 1670 (C═O) ketone str, 1631, 1604, 1546 Ar (C═C) str, 1477 (CH_2_) bend, 721 (C–Cl) str.

^1^HNMR (300 MHz, DMSO*_d_*_6_, *δ*, ppm): 7.97 (d, *J* = 9.1 Hz, 2H, ArH), 7.68 (s, 1H, ArH), 7.58 (d, *J* = 9.0 Hz, 1H, ArH), (7.38 (d, *J* = 9.1 Hz, 2H, ArH), 6.89 (d, *J* = 9.0 Hz, 1H, ArH), 6.19 (s, 2H, NHCH_2_), 5.09 (s, 2H, S–CH_2_), 2.40 (s, 3H, CH_3_).

^13^CNMR (75 MHz, DMSO*_d_*_6_, *δ*, ppm): 192.31 (C═O), 164.71 (COOH), 161.69 (C═N‐oxadiaz.), 148.04, 144.55, 132.52, 129.54, 129.24, 128.44, 127.64, 126.61, 116.75, 115.19, 110.57 (Ar–C), 40.76 (CH_2_–NH) 40.32 (CH_2_–S), 21.25 (CH_3_).

MS (ESI) *m/z*: Calcd. for C_19_H_16_ClN_3_O_4_S [M + 2]**^+^**: 419.06, found 419.00.

#### 6.2.12 General Method for Synthesis of Hydrazine‐1‐Carbothioamide Derivatives (Compounds **5An–5cn**) [47, 48]

A volume of 25 mL of ethanol was added to compound (**A2**) (0.000388 mol, 0.1 g) in round bottom flask to form a suspension, and then separately phenyl isothiocyanate (0.000388 mol, 0.052 g, 0.046 mL), 4‐bromo phenyl isothiocyanate (0.000388 mol, 0.099 g), and 4‐chloro phenyl isothiocyanate (0.000388 mol, 0.099 g) were added to the reaction mixture and stirred overnight. A volume of 30 mL of cold D.W. was added to the solution; the precipitate was filtered off and washed with cold EtOH to produce a product, which was recrystallized with EtOH 70%.

#### 6.2.13 3‐Chloro‐4‐((2‐(2‐((4‐Chlorophenyl)Carbamothioyl)Hydrazinyl)‐2‐Oxoethyl)Amino)Benzoic Acid (**A5an**)

White powder, mp = (218–220)°C, yield (70%), *R_f_* = 0.60 (*n*‐hexane/ethyl acetate 8:2).

FTIR (ύ, cm^−1^): 3471 (NH) str of amine, 3356 (NH) str of thioamide, 3313 (NH) amide, 3151 (OH) str of (COOH), 1654 (C═O) amide str, 1616 (NH) bend (amide), 1593, 1546, 1492 Ar (C═C) str, 1211 (C═S) str, 732 (C─Cl) str.

^1^HNMR (300 MHz, DMSO*_d_*_6_, *δ*, ppm): 10.21 (*br*s, 1H, NH), 9.78 (*br*s, 1H, NH), 9.68 (*br*s, 1H, NH), 7.86 (s, 1H, ArH), 7.85–7.36 (m, 5H, ArH), 6.81 (d, *J* = 8.0 Hz, 1H, ArH), 6.01 (*br*s, 2H, NH_2_ **^+^** Ar‐zwitterion).

^13^CNMR (75 MHz, DMSO*_d_*_6_, *δ*, ppm): 181.07 (C═S), 164.96 (COOH and CONHNH), 147.98, 138.26, 129.22, 128.91, 128.01, 127.78, 127.45, 120.12, 115.84, 113.94 (Ar─C), 28.97 (CH_2_–NH).

MS (ESI) *m/z*: Calcd. for C_16_H_14_Cl_2_N_4_O_3_S [M]**^+^**: 412.02, found 412.40.

#### 6.2.14 4‐((2‐(2‐((4‐Bromophenyl)Carbamothioyl)Hydrazinyl)‐2‐Oxoethyl)Amino)‐3‐Chlorobenzoic acid (**A5bn**)

White powder, yield (80%), mp = (215–217)°C, *R_f_* = 0.62 (*n*‐hexane/ethyl acetate 6:4).

FTIR (ύ, cm^−1^): 3468 (NH) str of amine, 3352 (NH) str of thioamide, 3259 (NH) str of amide, 3147 (OH) str of (COOH), 1654 (C═O) amide str, 1616 (NH) bend (amide), 1593, 1546, 1485 Ar (C═C) str, 1211 (C═S) str, 729 (C–Cl) str, 663 (C–Br) str.

^1^HNMR (300 MHz, DMSO*_d_*_6_, *δ*, ppm): 10.21 (*br*s, 1H, NH), 9.77 (*br*s, 1H, NH), 9.69 (*br*s, 1H, NH), 7.86 (s, 1H, ArH), 7.65–7.46 (m, 5H, ArH), 6.81 (d, *J* = 8.0 Hz, 1H, ArH), 6.01 (*br*s, 2H, NH_2_ **^+^** Ar‐zwitterion).

^13^CNMR (75 MHz, DMSO*_d6_*, *δ*, ppm): 181.10 (C═S), 164.95 (C═O), 147.99, 138.71, 130.70, 129.22, 128.01, 120.11, 115.84, 113.94 (Ar–C).

MS (ESI) *m/z*: Calcd. for C_16_H_14_BrClN_4_O_3_S [M + 3]**^+^**: 458.96, found 459.40.

#### 6.2.15 3‐Chloro‐4‐((2‐*oxo*‐2‐(2‐(Phenylcarbamothioyl) Hydrazinyl)Ethyl)Amino)Benzoic Acid (**A5cn**)

White powder, yield (75%), mp = (207–209)°C, *R_f_* = 0.58 (*n*‐hexane/ethyl acetate 6:4).

FTIR (ύ, cm^−1^): 3475 (NH) str of amine, 3309 (NH) str of thioamide, 3267 (NH) str of amide, 3159 (OH) str of (COOH), 1654 (C═O) amide str, 1616 (NH) bend (amide), 1593, 1531, 1496 Ar (C═C) str, 1215 (C═S) str, 752 (C–Cl) str.

^1^HNMR (300 MHz, DMSO*_d_*_6_, *δ*, ppm): 10.21 (*br*s, 1H, NH), 9.74 (*br*s, 1H, NH), 9.57 (*br*s, 1H, NH), 7.87 (s, 1H, ArH), 7.66–7.15 (m, 5H, ArH), 6.82 (d, *J* = 6.1 Hz, 1H, ArH), 6.01 (*br*s, 2H, NH_2_ **^+^** Ar‐zwitterion).

^13^CNMR (75 MHz, DMSO*_d_*_6_, *δ*, ppm): 181.84 (C═S), 165.68 (COOH), 148.64 (C─NH), 139.94 (C─NH), 129.93, 128.71, 128.58, 126.63, 125.62, 120.88, 116.51, 114.61 (Ar─C), 40.79 (CH_2_).

MS (ESI) *m/z*: Calcd. for C_16_H_15_ClN_4_O_3_S [M + 3]**^+^**: 381.06, found 381.50.

## 7 Molecular Docking Studies

### 7.1 Method of Docking Process

Molecular docking simulations were performed to assess the potential affinity of the tested compounds against the zinc‐dependent HDACs class I (HDAC1‐3, HDAC8) and II (HDAC4‐7, HDAC9‐10) according to previously published work (Supporting Information) [49].

### 7.2 Pharmacokinetic ADMET Study

The new compounds’ ADMET descriptors were assessed using the Discovery Studio 2016 Visualizer (Supporting Information).

### 7.3 Molecular Similarity

The molecular similarity (Table S6) of the tested compounds was assessed using the Discovery Studio 2016 Visualizer, with vorinostat (SAHA) and trichostatin serving as the reference HDACis (Supporting Information).

### 7.4 Toxicity Study

The Discovery Studio 2016 Visualizer was utilized to compute the toxicity parameters of the selected compounds, followed by a comprehensive evaluation using the Toxicity Prediction (Extensible) Protocol (TOPKAT) (Supporting Information).

### 7.5 Molecular Orbital Analysis

A comprehensive DFT analysis was conducted to investigate the spatial distribution of the molecular orbitals for the newly synthesized compounds. Total energy, binding energy, HOMO energy, LUMO energy, dipole magnitude, and band gap energy were calculated using the BIOVIA Discovery Studio. The reference compounds SAHA and trichostatin were also included in the calculations.

### 7.6 MD Simulations

The Desmond simulation package from Schrödinger LLC was utilized for conducting MD simulations according to previously published work (Supporting Information) [50, 51, 52].

## 8 Biological Studies

### 8.1 Cell Culture

The following human cell lines obtained from American Type Culture Collection (ATCC) were used to elucidate the antiproliferative effect of the newly synthesized compounds: HepG2 (HB‐8065; hepatocellular carcinoma), MCF‐7 (HTB‐22; breast cancer), K562 (CCL‐243; myeloid leukemia), and MCF‐10A (CRL‐10317; normal mammary cells). MCF‐7 and K562 cells were maintained in RPMI‐1640 media (Capricorn, Germany), whereas HepG2 cells were grown in high‐glucose DMEM (Capricorn, Germany). All media used were supplemented with 10% fetal bovine serum (Gibco, USA), 1% l‐glutamine (Gibco, USA), and 1% penicillin/streptomycin (Gibco, USA). MCF‐10A cells were cultured in DMEM/F12 media (Capricorn, Germany) supplemented with 5% horse serum (Gibco, USA), epidermal growth factor (EGF; Sigma, USA), insulin (Sigma, USA), hydrocortisone (Sigma, USA), and cholera toxin (Sigma, USA). Cells were maintained in humidified incubator with 5% CO_2_ at 37°C.

### 8.2 In Vitro Cytotoxicity Assay

To evaluate the effects of the compounds on cell proliferation and determine their inhibitory concentration (IC_50_), eight different concentrations (0.03, 0.1, 0.3, 1, 3, 10, 30, and 100 µM) of each compound were tested. Cells were seeded at a density of 5 × 10^3^ cells per well in a 96‐well plate and incubated for 24 h to allow cell attachment. After 72 h of treatment, the media was removed, and 0.5 µg/µL MTT (3‐[4,5‐dimethylthiazol‐2‐yl]‐2,5‐diphenyltetrazolium bromide) was added. After a 4‐h incubation period, DMSO was added for 30–45 min to dissolve the formazan crystals formed. The absorbance was measured at 570 nm using a plate reader (Biotek Synergy HT Multi‐Mode Microplate Reader, USA), and the IC_50_ values were calculated accordingly. The experiment was performed three times with triplicates for each concentration.

### 8.3 HDAC Enzyme Activity

Among the cancer cell lines tested, MCF‐7 breast cancer cells showed the highest sensitivity to the novel compounds and were selected for further analysis. HDAC activity in response to the new compounds was measured using the HDAC‐Glo I/II Assay and Screening System (Promega, USA), following the manufacturer's instructions. Briefly, MCF‐7 cells were seeded in a 96‐well plate at a density of 10 × 10^3^ cells per well. After 24 h, the medium was replaced with serum‐free medium containing the different compounds. Additionally, SAHA and trichostatin were included as reference drugs. All compounds were tested at concentrations of 0.3, 3, 30, and 100 µM in triplicates. Cells were incubated with the drugs for 2 h before adding the HDAC‐Glo I/II reagent. The plate was gently mixed and incubated for 30 min. Luminescence signals were measured at 405 nm using a plate reader (Biotek Synergy HT Multi‐Mode Microplate Reader, USA). The experiment was run twice.

### 8.4 Immunoblotting

On the basis of results of the MTT and HDAC activity assays, compound **A3bn** demonstrated the best results and was therefore selected for further analysis. Immunoblotting was performed to evaluate changes in the levels of acetylated histone H3 and histone H4, the main targets of HDAC enzymes. After 72 h of treatment with **A3bn** (IC_50_) and SAHA (IC_50_), control untreated and treated MCF‐7 cells were washed twice with PBS and lysed using cold SDS lysis buffer. Protein concentrations were measured using the Bradford protein assay (BioRad, USA).

Proteins were separated on a 12%–15% SDS–PAGE gel and transferred onto a 0.45 µm nitrocellulose membrane (Santa Cruz Biotechnology, USA). The membranes were incubated overnight at 4°C with the following primary antibodies: rabbit anti‐Histone H3 Acetyl Lys9 (1:500; Abbkine, USA), rabbit anti‐Histone H4 Acetyl Lys12 (1:500; Abbkine, USA), and mouse anti‐GAPDH (1:2000; Invitrogen, USA). Following overnight incubation, the membranes were washed three times with TBST and incubated with the appropriate HRP‐conjugated secondary antibodies (R&D Systems, USA). After a 1‐h incubation, the membranes were washed again with TBST, and protein signals were developed using Pierce ECL western blotting substrate (Thermo Scientific) and visualized using the FluorChem R system (Oxford, UK).

### 8.5 Cell Apoptosis

Apoptosis in control MCF‐7 breast cancer cells and MCF‐7 cells treated with **A3bn** (IC_50_) and SAHA (IC_50_) for 72 h were detected using the TdT In Situ Apoptosis (TUNEL) Detection Kit‐Fluorescein (R&D Systems, USA) according to the manufacturer's instructions.

### 8.6 Quantitative Reverse Transcription‐Polymerase Chain Reaction (qRT‐PCR)

Caspase deregulation in response to **A3bn** and SAHA treatment compared to control untreated cells was assessed at the RNA level using qRT‐PCR. RNA was extracted using Trizol reagent, followed by purification with the Direct‐zol RNA Miniprep Extraction Kit (Zymo Research, USA). cDNA synthesis was performed using Takara PrimeScript RT Master Mix (Takara Bio Inc, USA). The qPCR mix was prepared as follows: 12.5 µL master mix (KAPA Biosystems, USA), 1 µL each of forward and reverse primers, 1 µL of cDNA, and nuclease‐free water to a final reaction volume of 20 µL. qPCR amplification was carried out using the Line Gene 9680 BioER instrument with cycling parameters detailed as follows: activation stage at 50°C for 2 min; presoak stage at 95°C for 10 min; 40 cycles of denaturation at 95°C for 15 s, followed by annealing and extension at 60°C for 1 min; and finally, melting curve stage: 95°C for 15 s, 60°C for 15 s, 95°C for 15 s. Relative expression levels were analyzed using the ΔΔ*Ct* method, with fold changes normalized to housekeeping gene (GAPDH).

### 8.7 Caspases Enzymatic Assay

Control untreated MCF‐7 cells and cells treated with **A3bn** and SAHA at the IC_50_ concentration were incubated for 72 h, with duplicate samples prepared for each caspase (3, 4, 8, or 9). Caspase activity assay was performed using the colorimetric caspase assay kit from Abbkine following the manufacturer's protocol. Briefly, cells were collected, washed, and lysed in the working cell lysis buffer provided. The lysates were incubated on ice for 20 min, centrifuged at 16 000 *g* at 4°C for 15 min, and the supernatants were transferred to fresh tubes. Aliquots were added to a 96‐well plate, mixed with the working reaction buffer and the appropriate caspase substrate, and incubated at 37°C for 2 h. Absorbance was then measured at 405 nm using the Biotek Synergy HT Multi‐Mode Microplate Reader (USA). The experiment was run twice.

### 8.8 Statistical Analysis

The Student *t*‐test (GraphPad Prism 9) was used to determine the statistical significance. Statistical significance was defined as a *p* value less than 0.05 (*p* < 0.05).

## Author Contributions

**Nedaa A. Abd Al Rahim**: data curation, formal analysis, methodology, writing – original draft. **Ammar A. Razzak Mahmood**: writing – original draft, project administration, funding acquisition, supervision, writing – review and editing. **Lubna H. Tahtamouni**: writing – original draft, project administration, funding acquisition, supervision, writing – review and editing. **Randa M. Bawadi**: data curation, formal analysis, methodology. **Ayah Y. Almasri**: data curation, formal analysis, methodology. **Marya A. Hamad**: data curation, formal analysis, methodology. **Nour A. Hussein**: data curation, formal analysis, methodology. **Salem R. Yasin**: project administration, funding acquisition. **Abdulrahman M. Saleh**: methodology, software.

## Conflicts of Interest

The authors declare no conflicts of interest.

## Supporting information




**Supporting File 1**: cbdv70450‐sup‐0001‐SuppMat.pdf

## Data Availability

The data that support the findings of this study are available in the Supporting Information of this article.
