# Supplementary material for: Synthesis, In Silico, and Biological Evaluation of Non‐Hydroxamate Benzoic Acid–Based Derivatives as Potential Histone Deacetylase Inhibitors (HDACi)
Source: Chem Biodivers. 2025 Sep 2;22(12):e01492. doi: 10.1002/cbdv.202501492 (PMC12716022; doi:10.1002/cbdv.202501492)

Experimental

Molecular docking studies

Method of docking process

Molecular docking simulations were performed to assess the potential affinity of the tested compounds against the zinc-dependent HDACs class I (HDAC1-3, HDAC8) and II (HDAC4-7, HDAC9-10) according to previously published work.[46] Initially, water molecules and unnessesary molecules were removed from the protein complexes. Afterwards, crystallographic disorders and unfilled valence atoms were corrected, and the protein structures energies were minimized. The 2D structure of each compound was drawn using Chem-Bio Draw Ultra16.0, saved as an SDF file, and then converted to a 3D structure. Protonation and energy minimization were carried out using the MMFF94 force field. The prepared ligands were docked into the target (HDACs) using Autodock Vina 1.5.7 software.

The protein target was held rigid while the ligands were allowed to be flexible. During the docking refinement, each molecule was allowed to generate twenty different poses. The docking scores (affinity energy) of the best-fitted poses with the active sites were recorded, and 3D and 2D figures were generated using the Discovery Studio 2016 Visualizer.

Pharmacokinetic ADMET study

The new compounds' absorption, distribution, metabolism, excretion, and toxicity (ADMET) descriptors were assessed using the Discovery Studio 2016 Visualizer. The CHARMM force field was initially used for compound setup and minimization. The evaluation covered critical parameters such as human intestinal absorption, aqueous solubility, blood-brain barrier penetration, plasma protein binding, CYP2D6 inhibition, and hepatotoxicity. The prepared compounds were selected as input ligands, and all relevant ADMET parameters were included for analysis. The protocol output was visualized, producing an interpretable ADMET chart.

Molecular similarity

The molecular similarity of the tested compounds was assessed using the Discovery Studio 2016 Visualizer, with vorinostat (SAHA) and trichostatin serving as the reference HDAC inhibitors (HDACi). We aimed to identify which candidate is most similar to SAHA and trichostatin. The analysis included quantitative parameters such as the number of rotatable bonds, rings, aromatic rings, hydrogen bond donors, hydrogen bond acceptors, logp, molecular weight, and MFPSA (Supplementary Table 6). After preparing the compounds, the small molecules panel with library

analysis was employed. Similar molecules were identified based on numeric properties, using the prepared compounds as input ligands.

Toxicity study

The Discovery Studio 2016 Visualizer was utilized to compute the toxicity parameters of the selected compounds, followed by a comprehensive evaluation using the Toxicity Prediction (Extensible) Protocol (TOPKAT). TOPKAT employs validated quantitative structure-toxicity relationship models to assess the compounds' toxicity based solely on their molecular structure. After compound preparation, the small molecules panel activated the toxicity prediction (extensible) option. The prepared compounds served as input ligands, and the toxicity model was selected. The detailed report task was set to generate a PDF file, and the protocol output was visualized, presenting a concise toxicity report.

Molecular dynamic (MD) simulations

The Desmond simulation package from Schrödinger LLC was utilized for conducting molecular dynamics (MD) simulations) according to previously published work. [47-49] The NPT ensemble, with a temperature set at 300 K and a pressure of 1 bar, was consistently employed across all runs. The simulations ran for 100 ns, with a relaxation time of 1 ps for the tested ligands. OPLS_2005 force field parameters were employed for all simulations. Long-range electrostatic interactions were computed using the particle mesh Ewald method, with a cutoff radius of 9.0 Å for Coulomb interactions. [48]

Water molecules were represented using the simple point charge model. Pressure control utilized the Martyna–Tuckerman–Klein chain-coupling scheme with a coupling constant of 2.0 ps, while temperature control employed the Nosé–Hoover chain-coupling scheme. Nonbonded forces were calculated using r-RESPA integrator, with short-range forces updated every step and long-range forces updated every three steps. Trajectories were saved at 4.8 ps intervals for subsequent analysis. The behavior and interactions between ligands and HDACs were examined using the Simulation Interaction Diagram tool within the Desmond MD package. The stability of MD simulations was assessed by monitoring the root mean square deviation (RMSD) of ligand and protein atom positions over time.

Additionally, the AMBER 14 package,[49] with the AMBER force field ff99, was employed for various tasks, including minimization, the addition of counterions, solvation, equilibration, and running periodic box, explicit water (TIP4P) MD simulations for the tested ligands. The structures

of the tested ligands were optimized using the density functional theory B3LYP method with a 6-31G basis set, and parameters were set to the GAFF force field. The protein-ligand–water system was allowed to be flexible during simulations, which comprised 10 independent runs with different random initial velocities. Each run spanned 10 ns, utilizing a timestep of 0.001 ps (1 fs). These multiple MD simulations, recognized for their wide acceptance, could adequately sample conformational space compared to longer, single-trajectory simulations. Data analysis was conducted using the CPPTRAJ program from the AMBER Tools distribution.

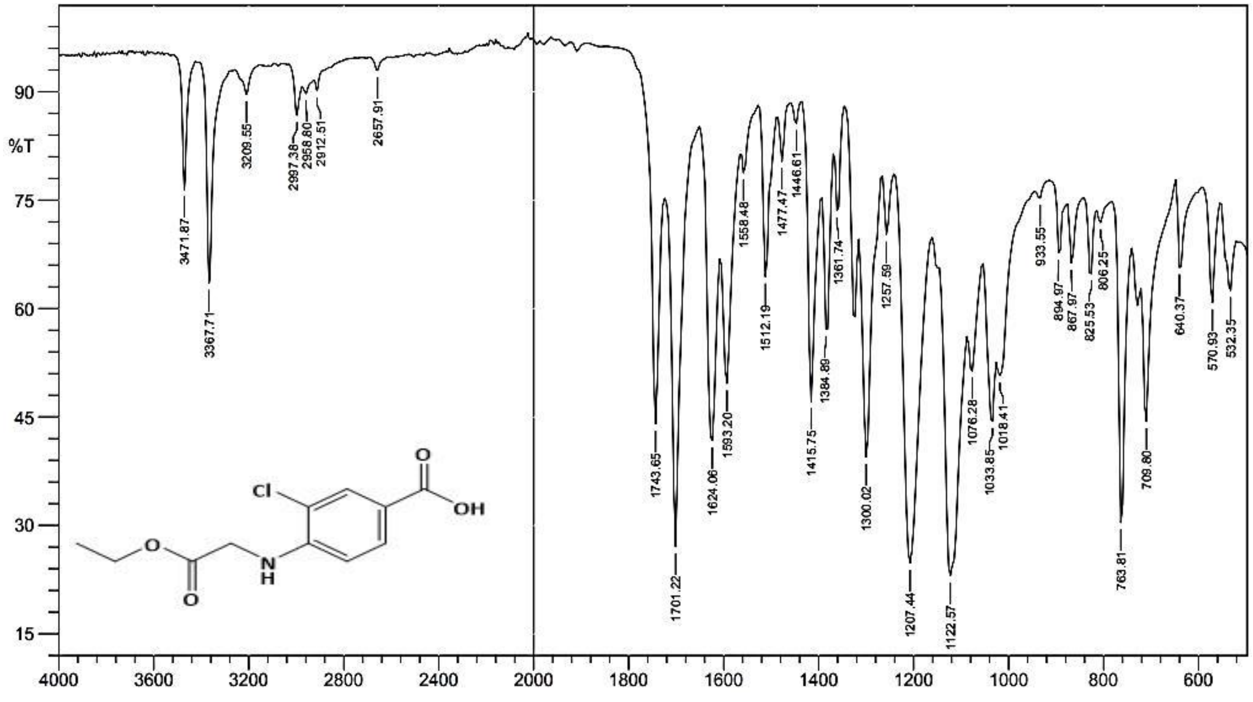

Supplementary Figure 1A. ATR-FTIR spectrum of the Compound A1.

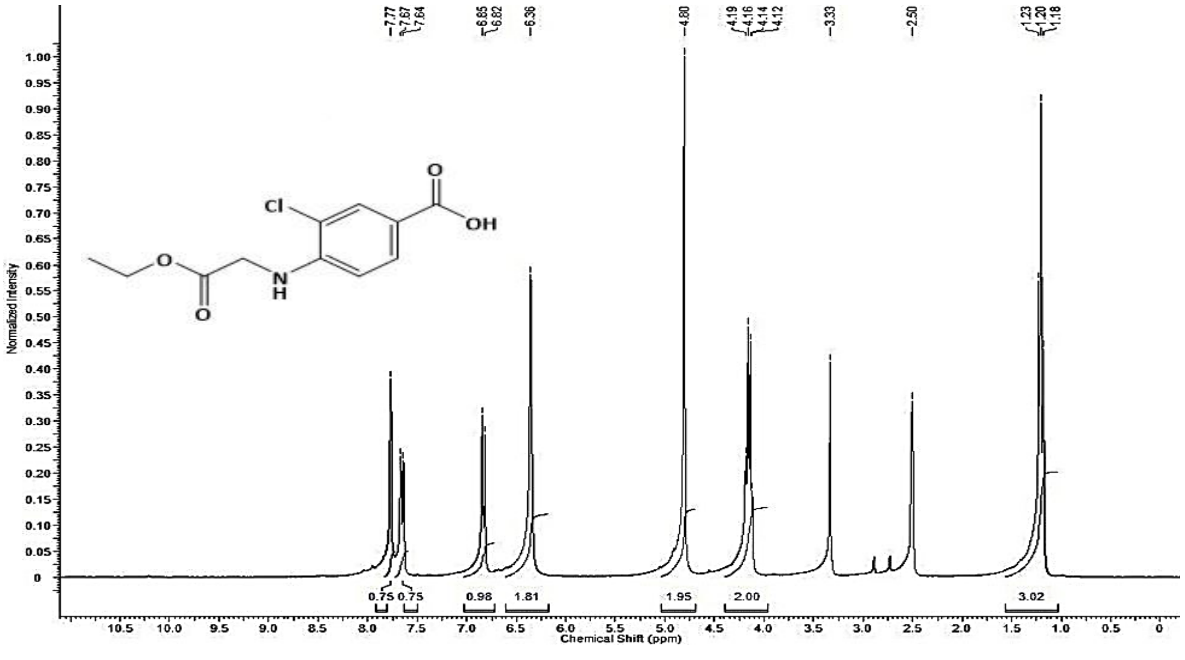

Supplementary Figure 1B. ^{1}HNMR spectrum of the Compound A1.

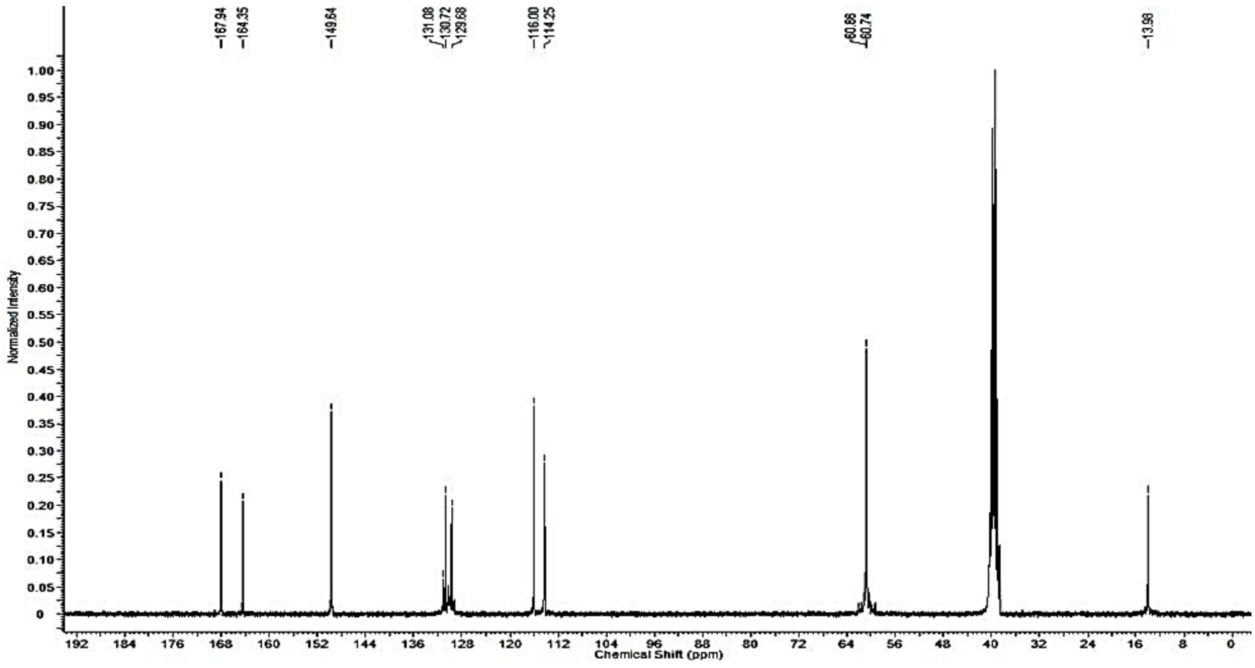

Supplementary Figure 1C. ^{13}CNMR spectrum of the Compound A1.

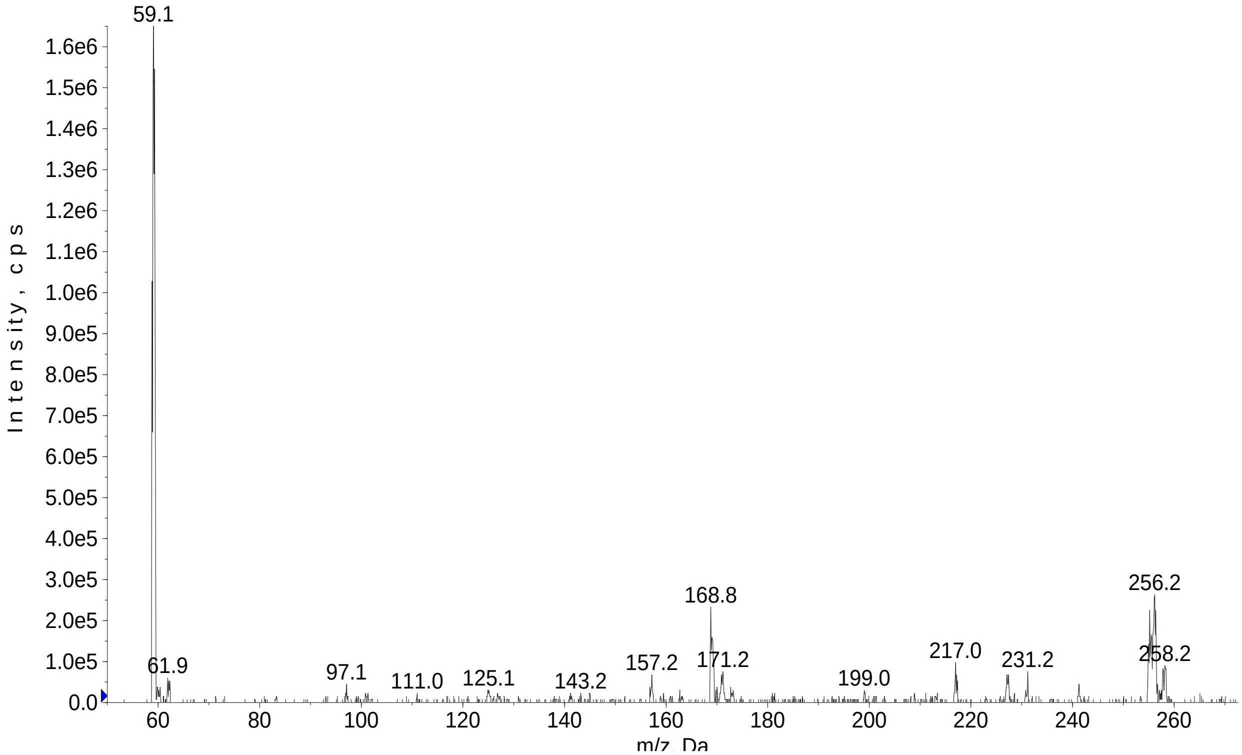

Supplementary Figure 1D. MS spectrum of the Compound A1.

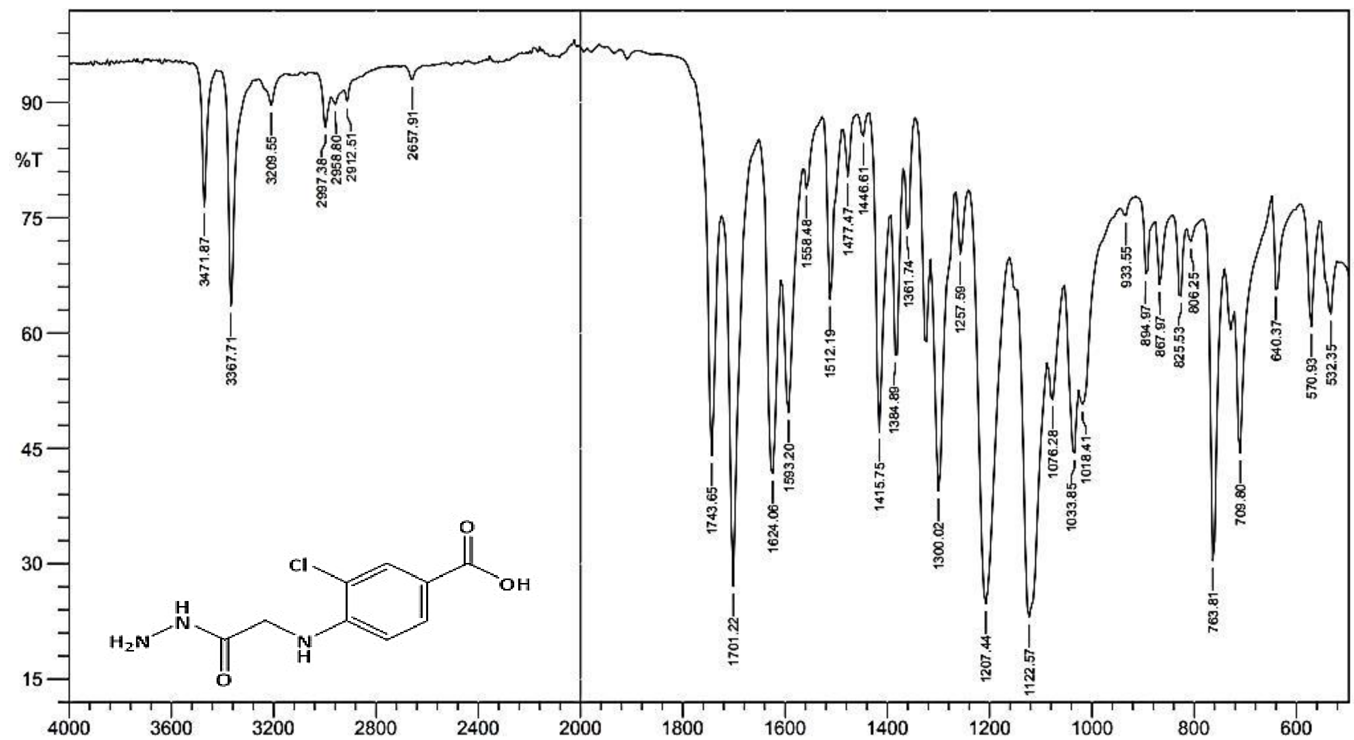

Supplementary Figure 2A. ATR-FTIR spectrum of the Compound A2.

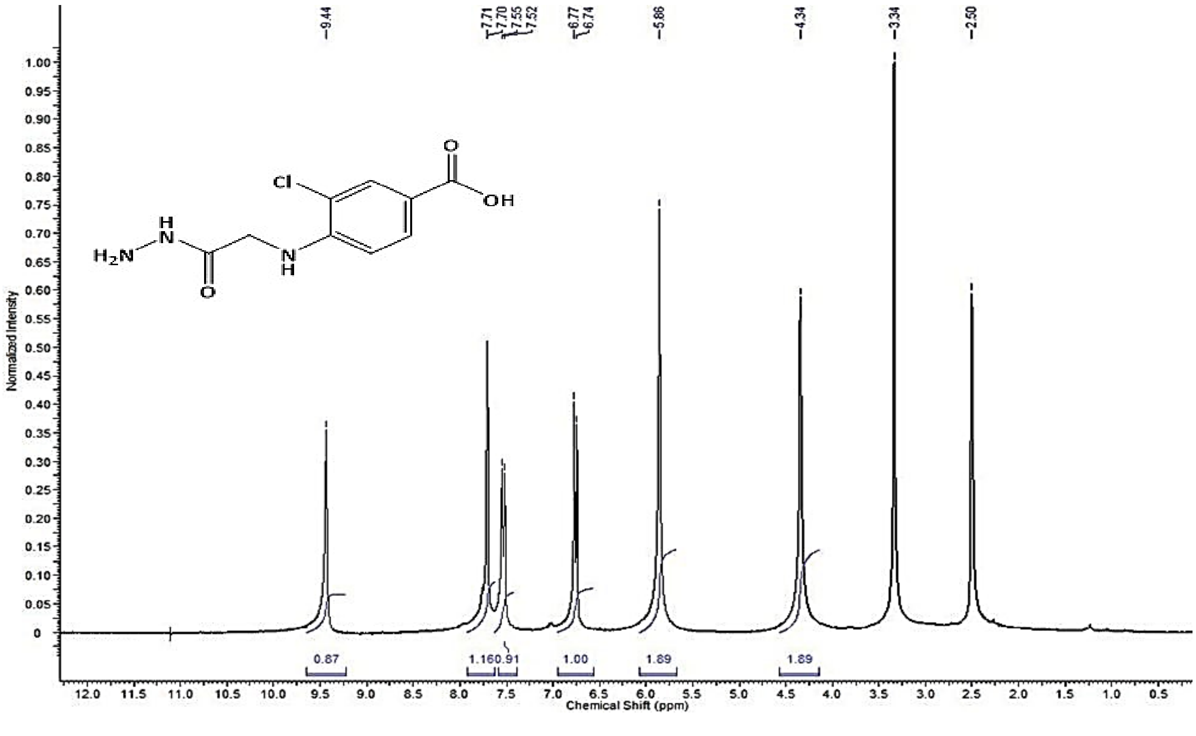

Supplementary Figure 2B. ^{1}HNMR spectrum of the Compound A2.

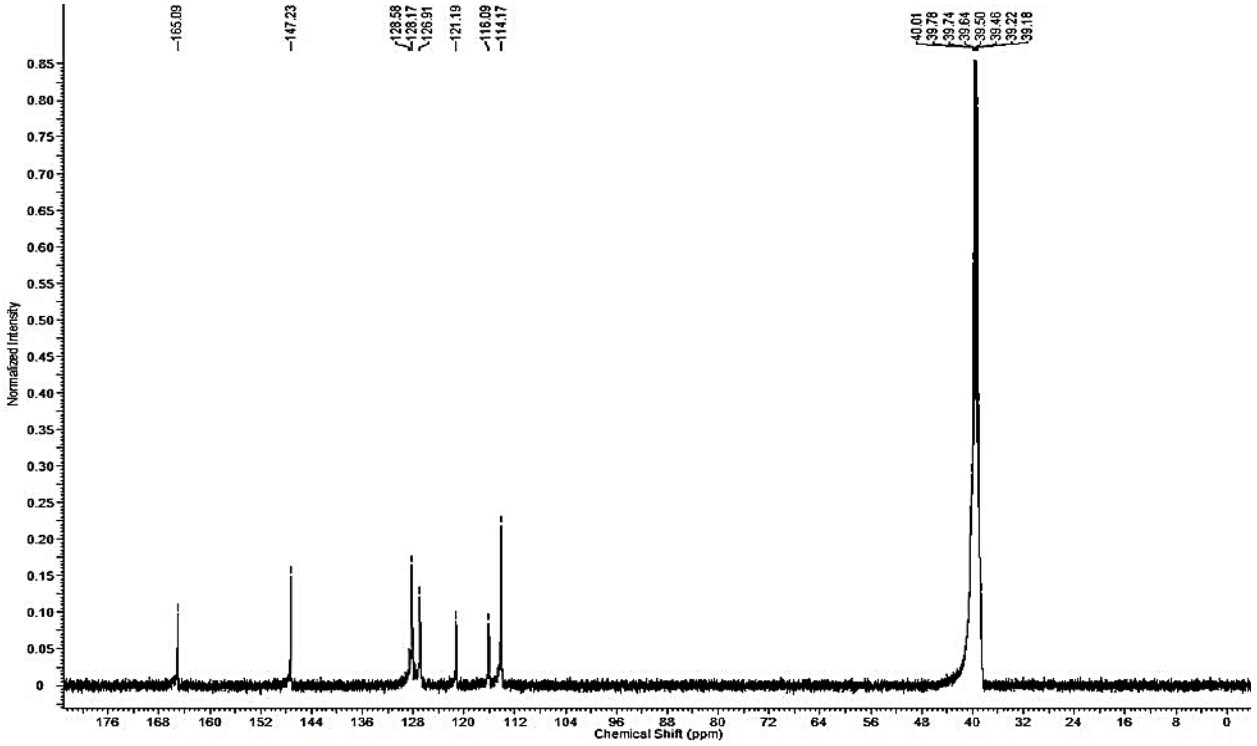

Supplementary Figure 2C. ^{13}CNMR spectrum of the Compound A2.

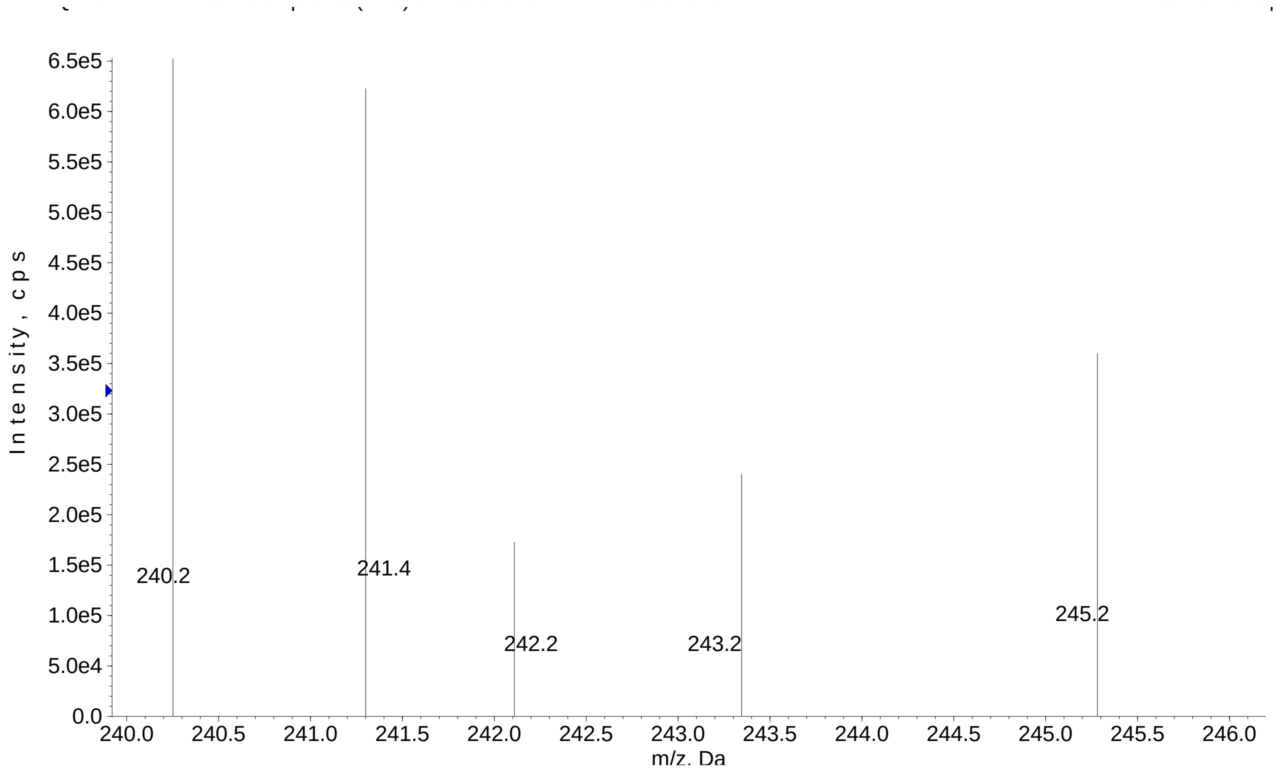

Supplementary Figure 2D. MS spectrum of the Compound A2.

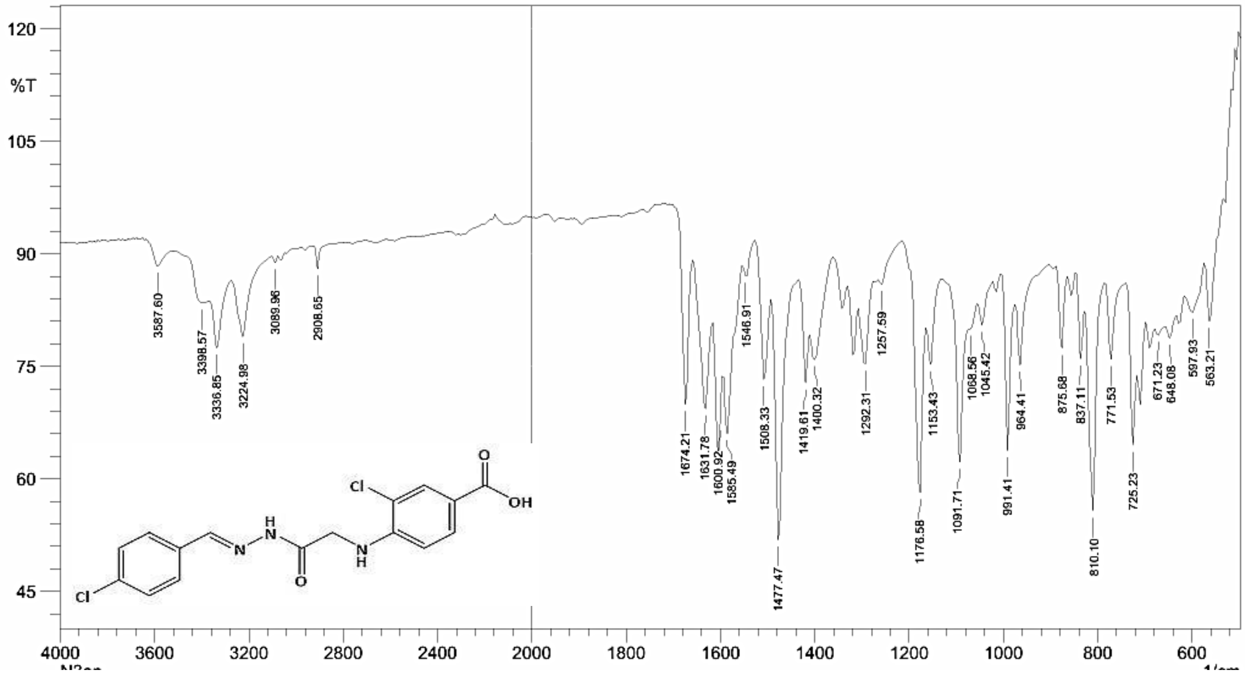

Supplementary Figure 3A. ATR-FTIR spectrum of the Compound A3an.

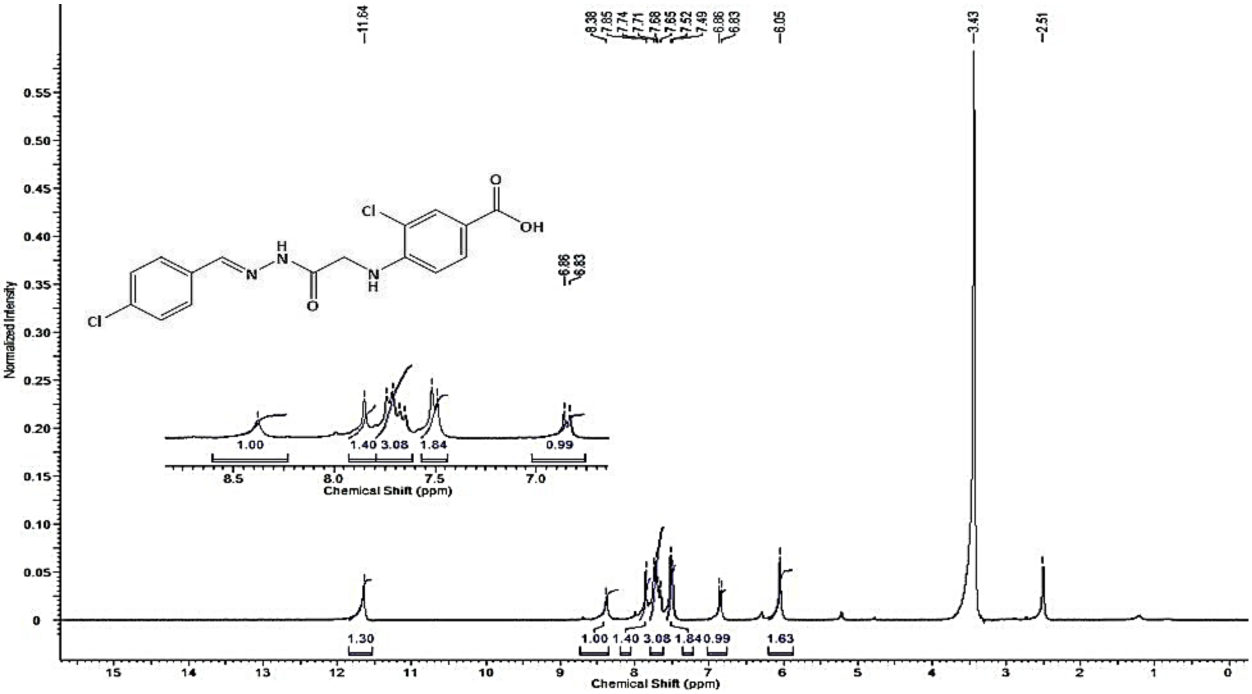

Supplementary Figure 3B. ^{1}HNMR spectrum of the Compound A3an.

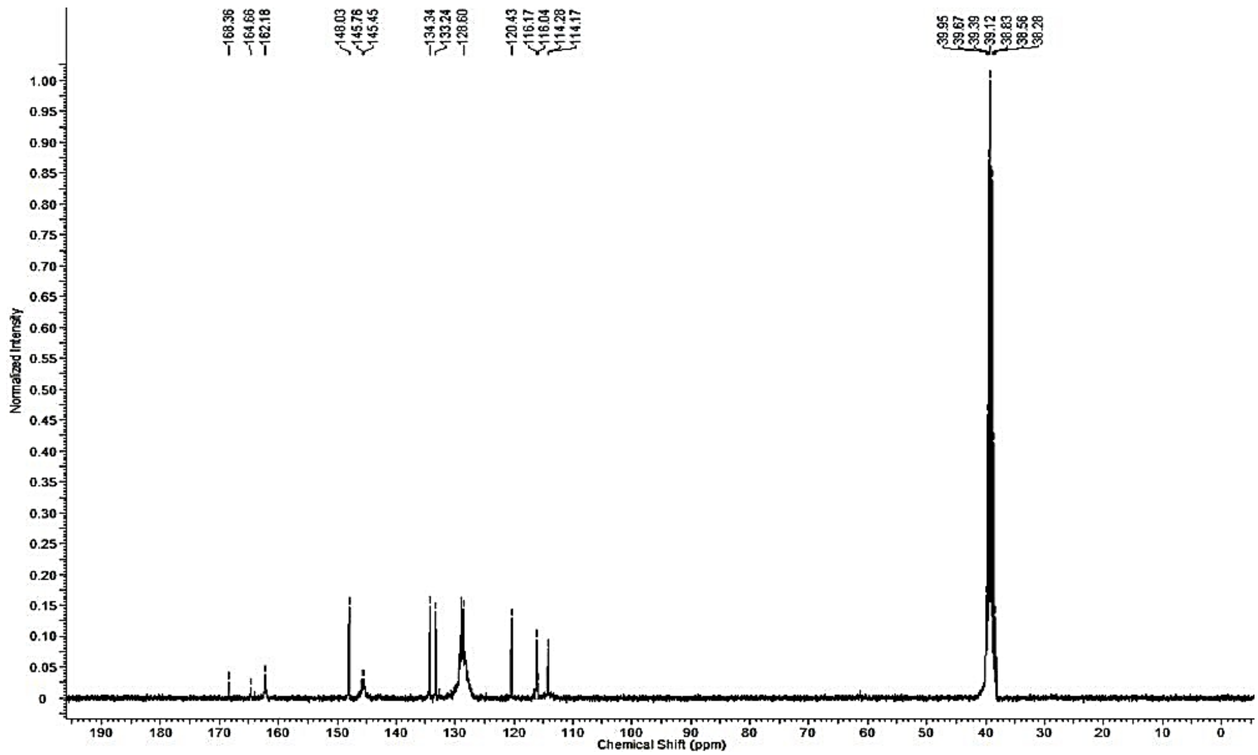

Supplementary Figure 3C. ^{13}CNMR spectrum of the Compound A3an.

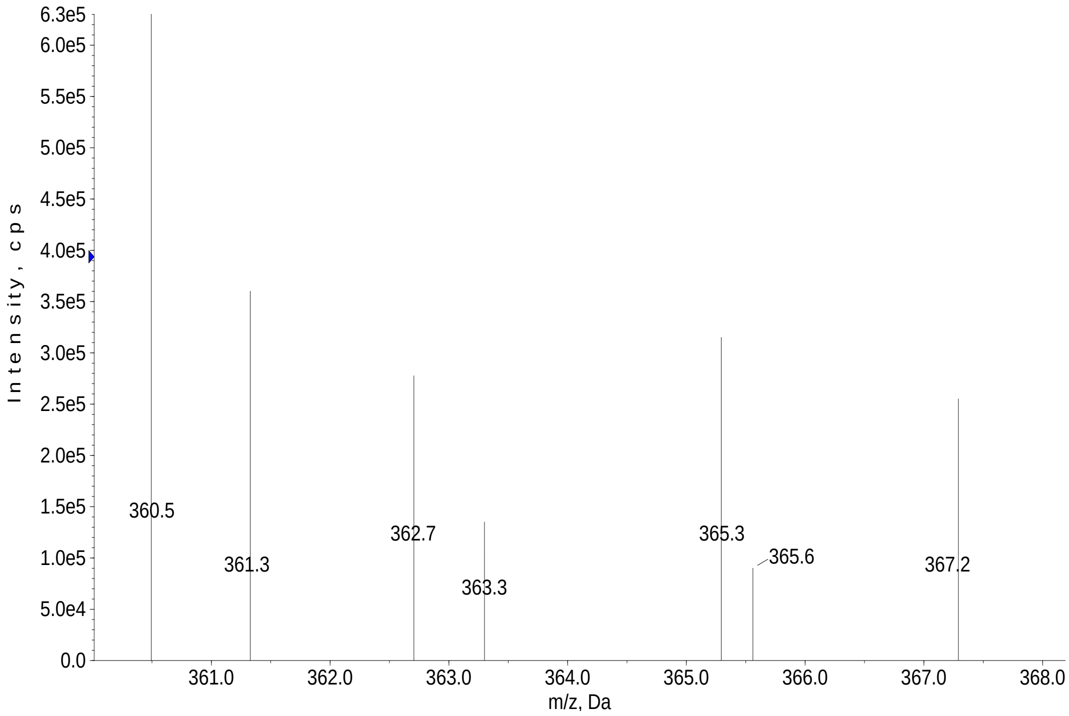

Supplementary Figure 3D. MS spectrum of the Compound A3an.

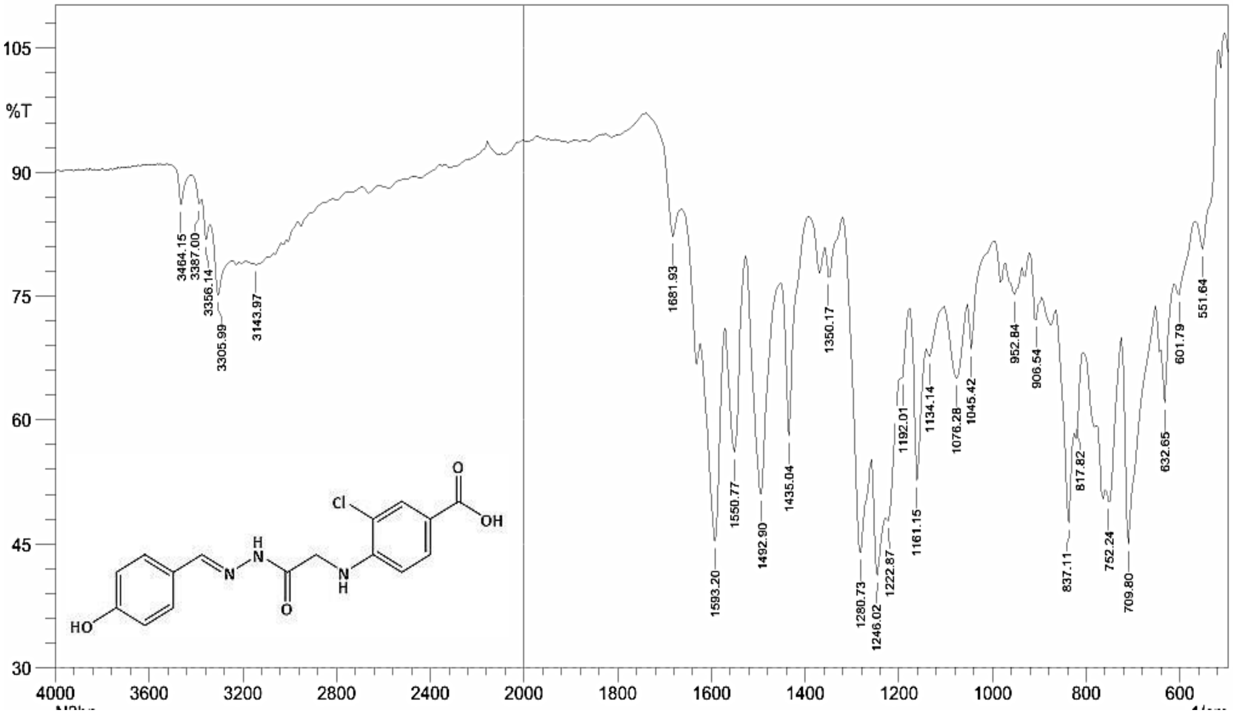

Supplementary Figure 4A. ATR-FTIR spectrum of the Compound A3bn.

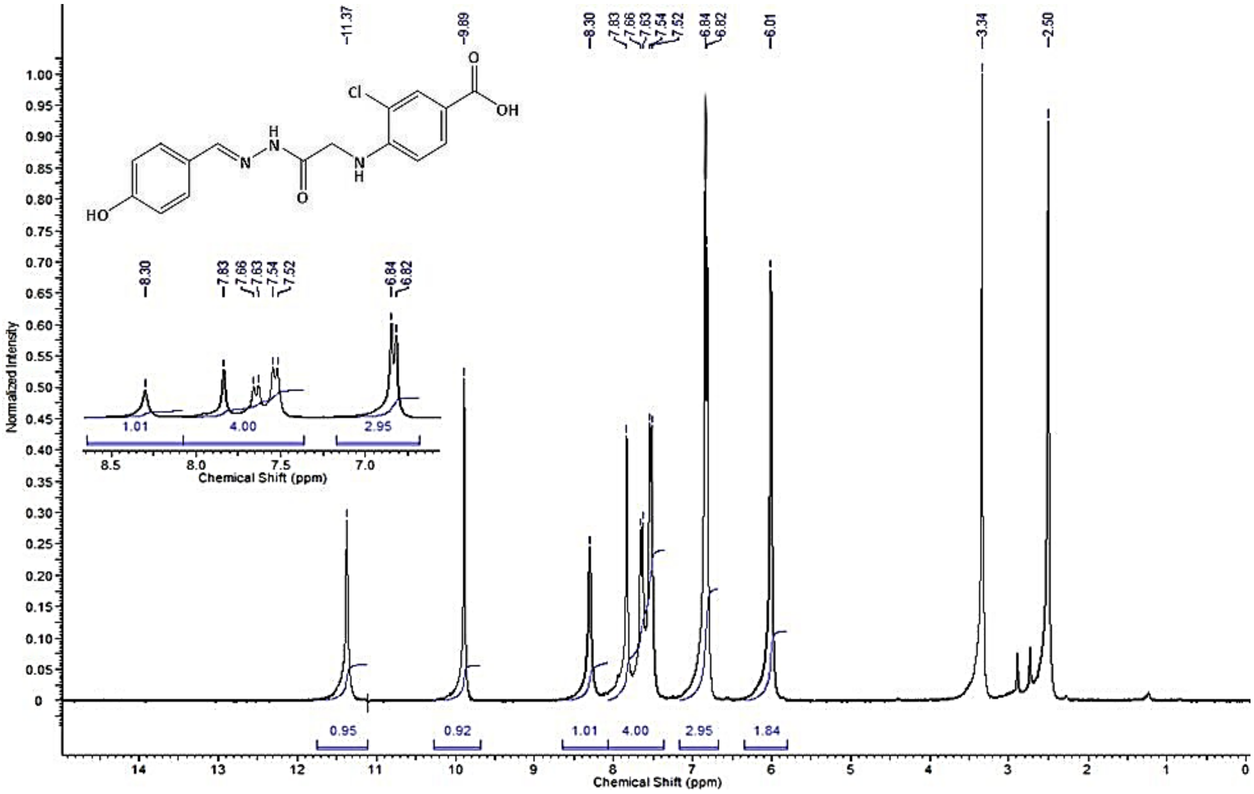

Supplementary Figure 4B. ^{1}HNMR spectrum of the Compound A3bn.

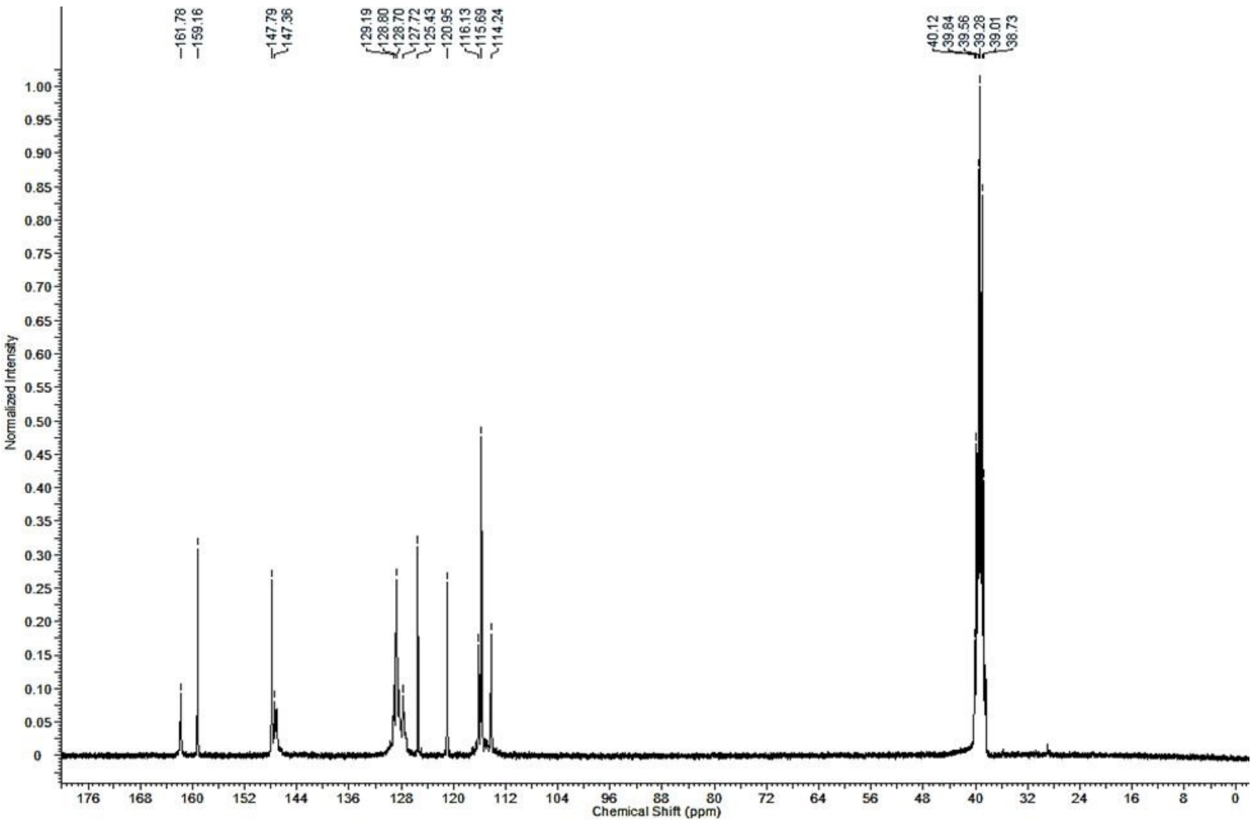

Supplementary Figure 4C. ^{13}CNMR spectrum of the Compound A3bn.

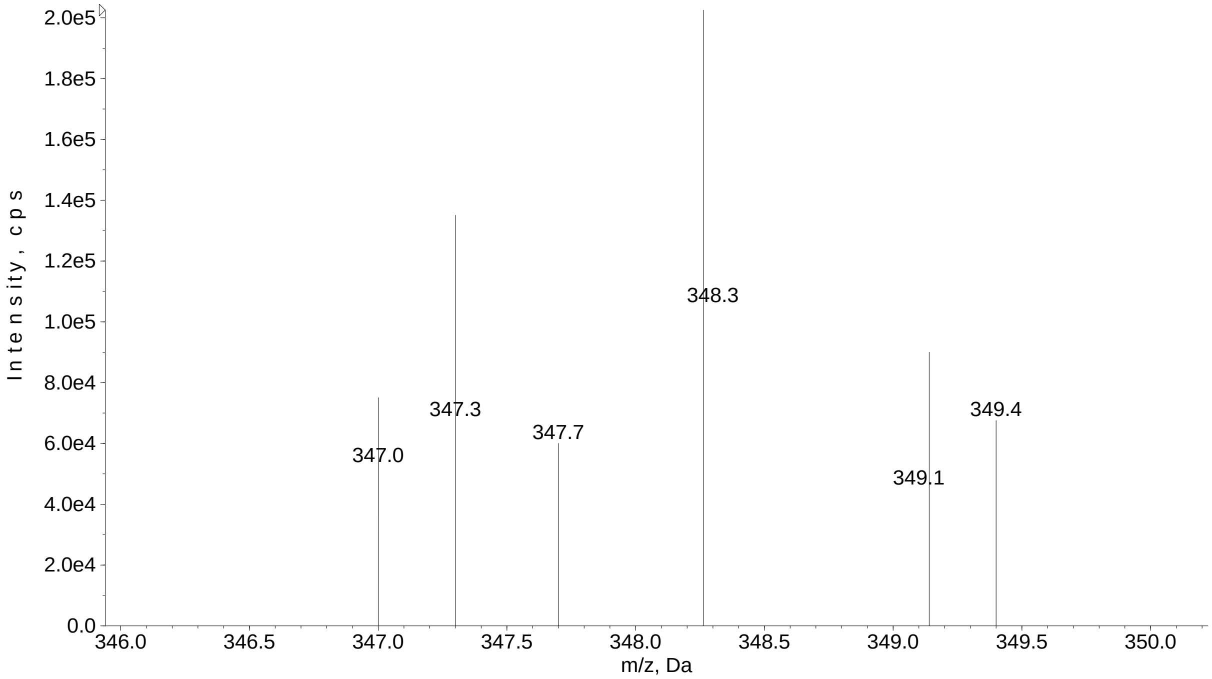

Supplementary Figure 4D. MS spectrum of the Compound A3bn.

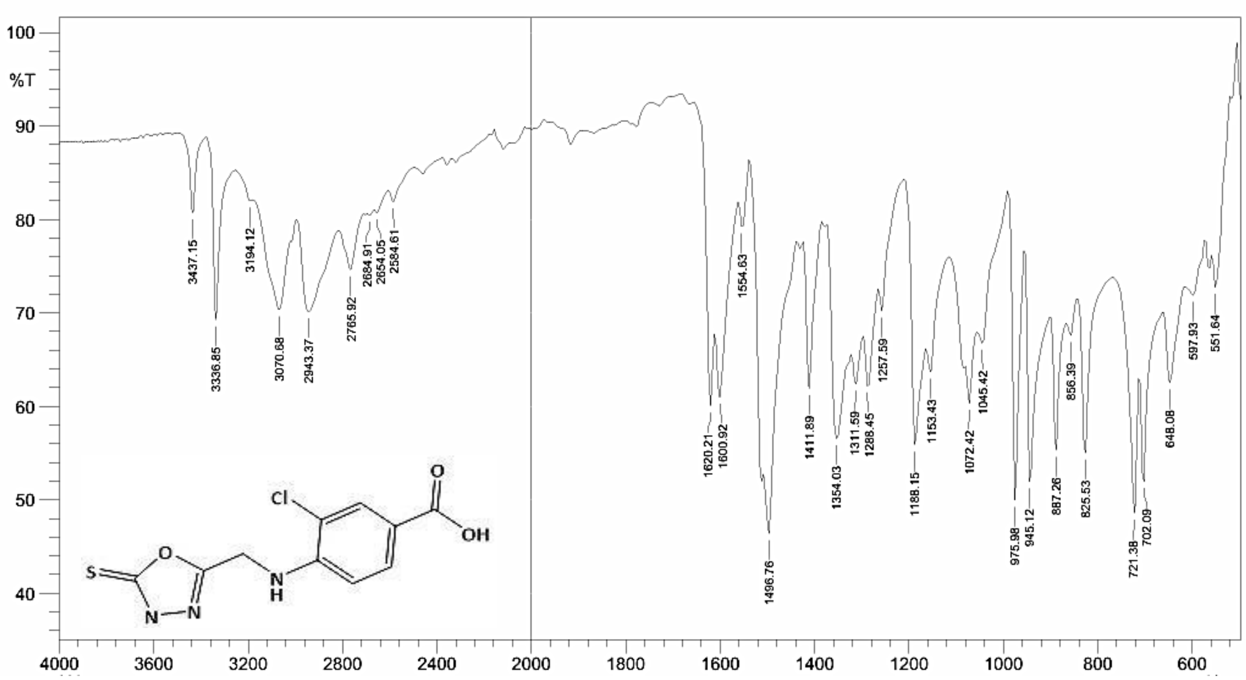

Supplementary Figure 5A. ATR-FTIR spectrum of the Compound A4.

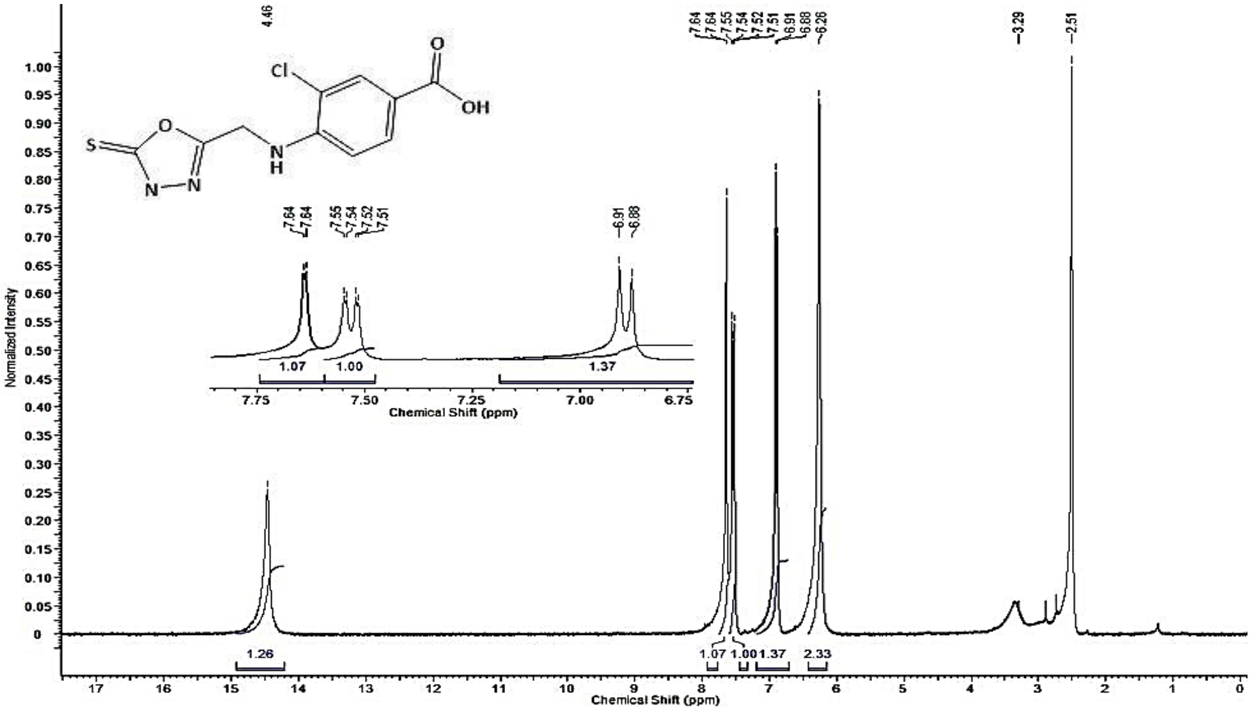

Supplementary Figure 5B. ^{1}HNMR spectrum of the Compound A4.

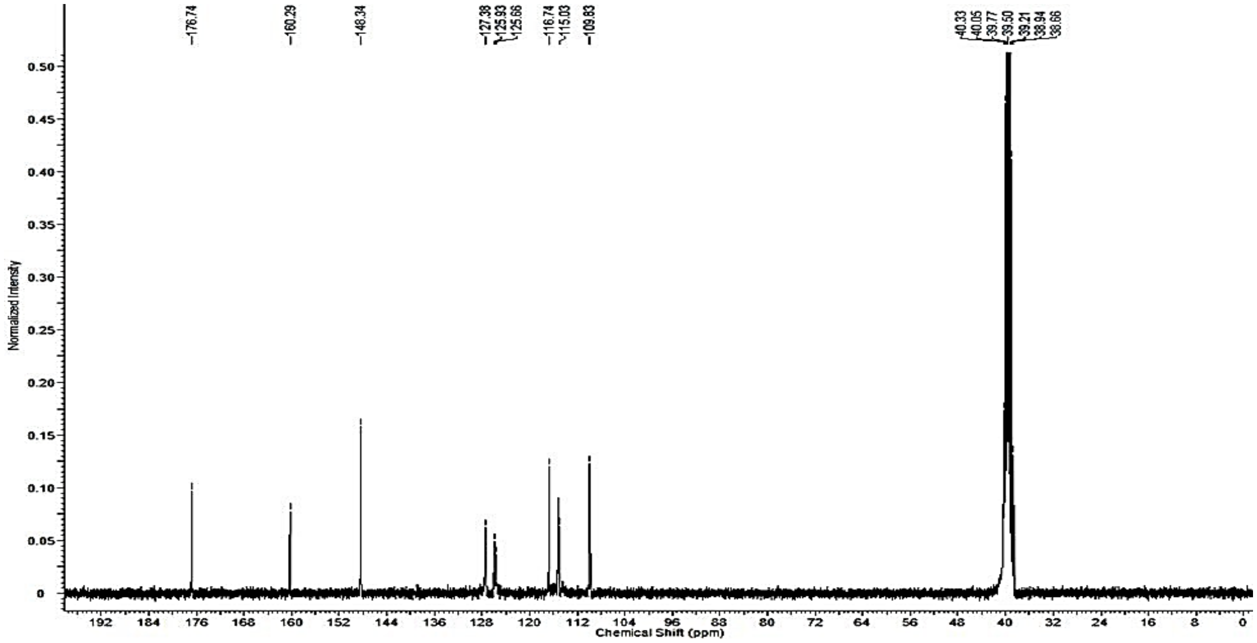

Supplementary Figure 5C. ^{13}CNMR spectrum of the Compound A4.

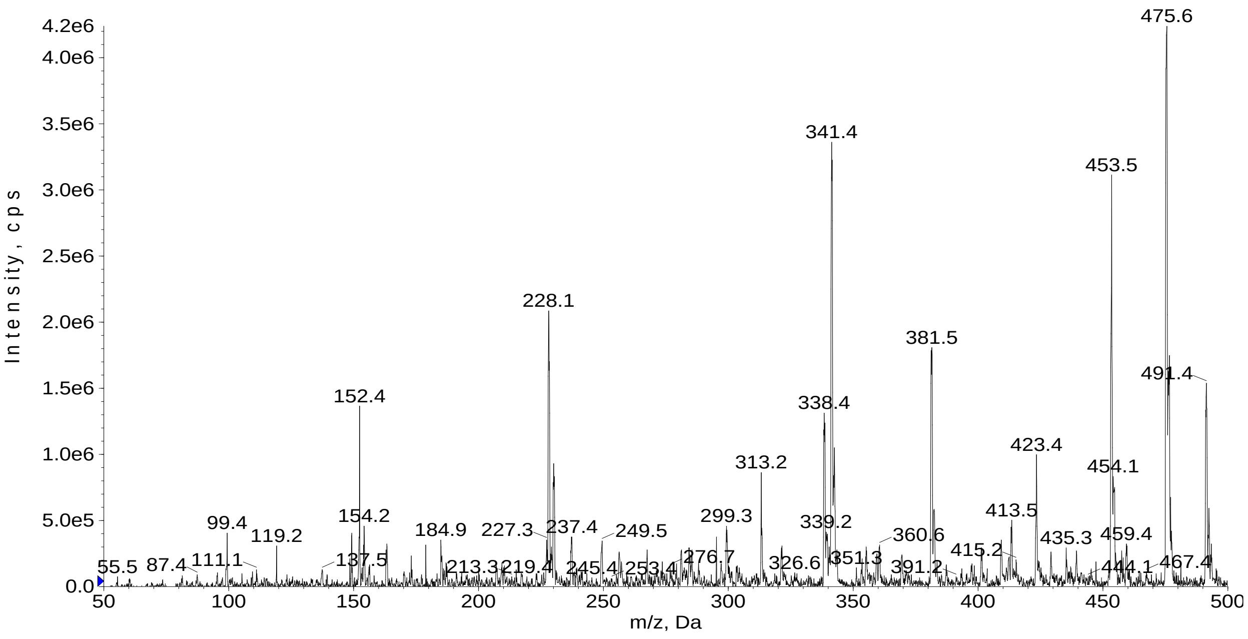

Supplementary Figure 5D. MS spectrum of the Compound A4.

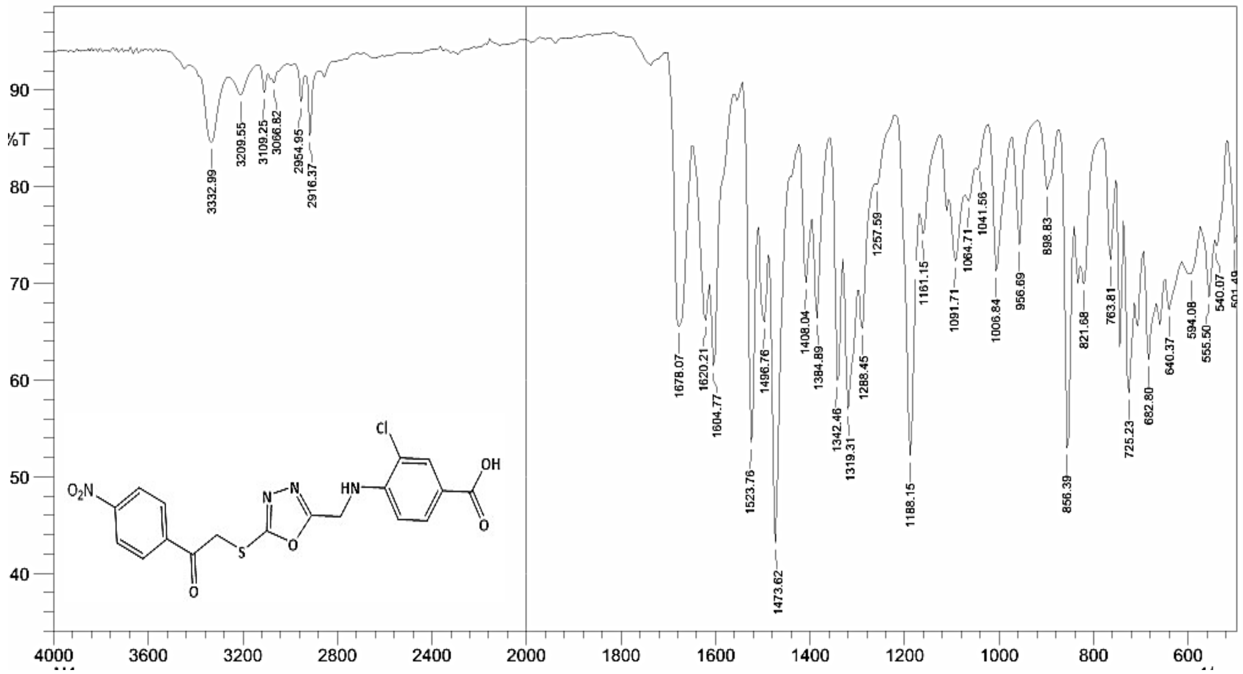

Supplementary Figure 6A. ATR-FTIR spectrum of the Compound A4an.

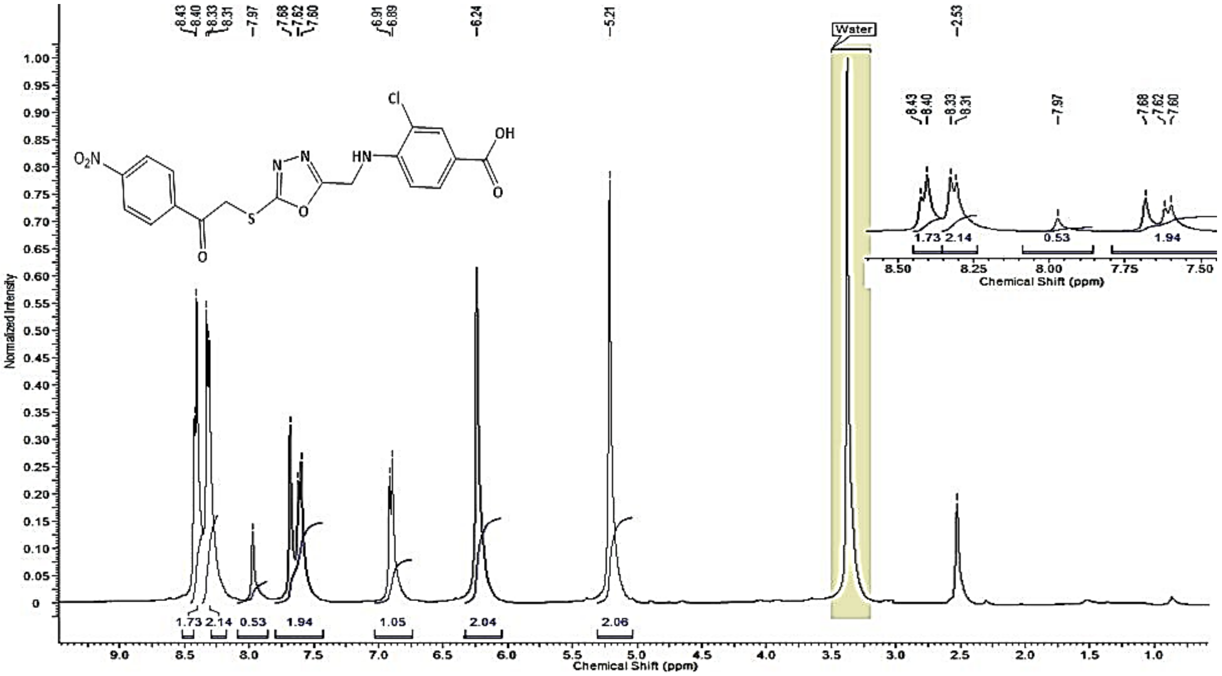

Supplementary Figure 6B. ^{1}HNMR spectrum of the Compound A4an.

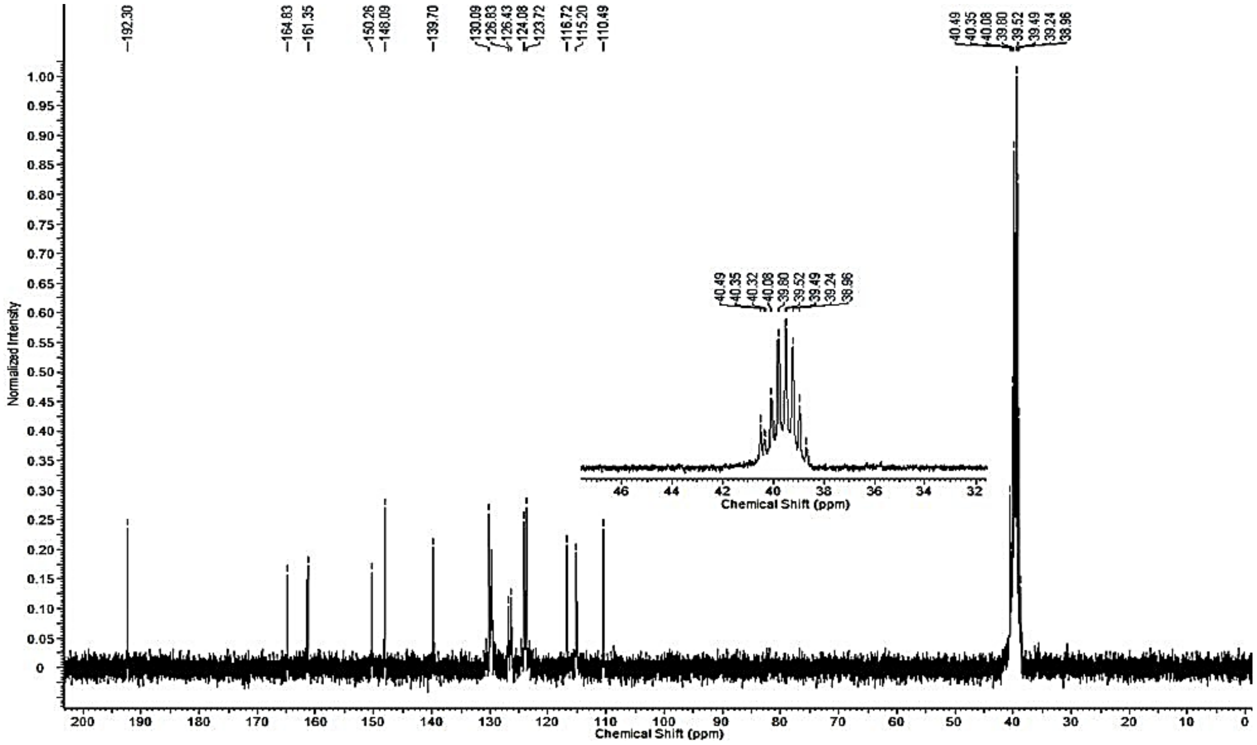

Supplementary Figure 6C. ^{13}CNMR spectrum of the Compound A4an.

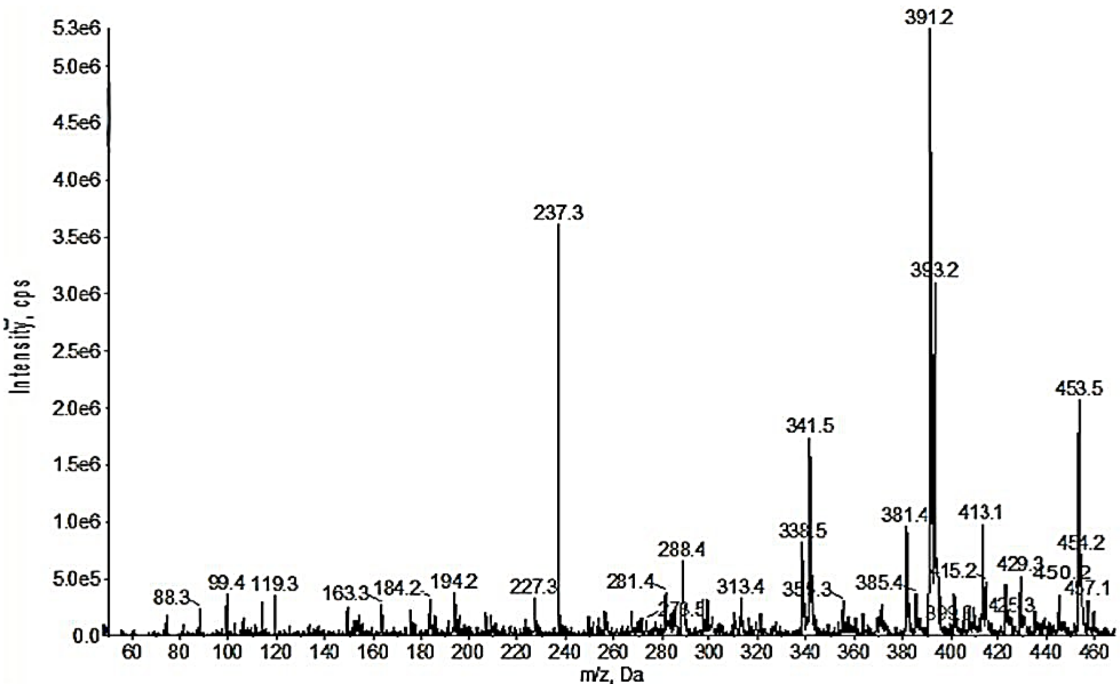

Supplementary Figure 6D. MS spectrum of the Compound A4an.

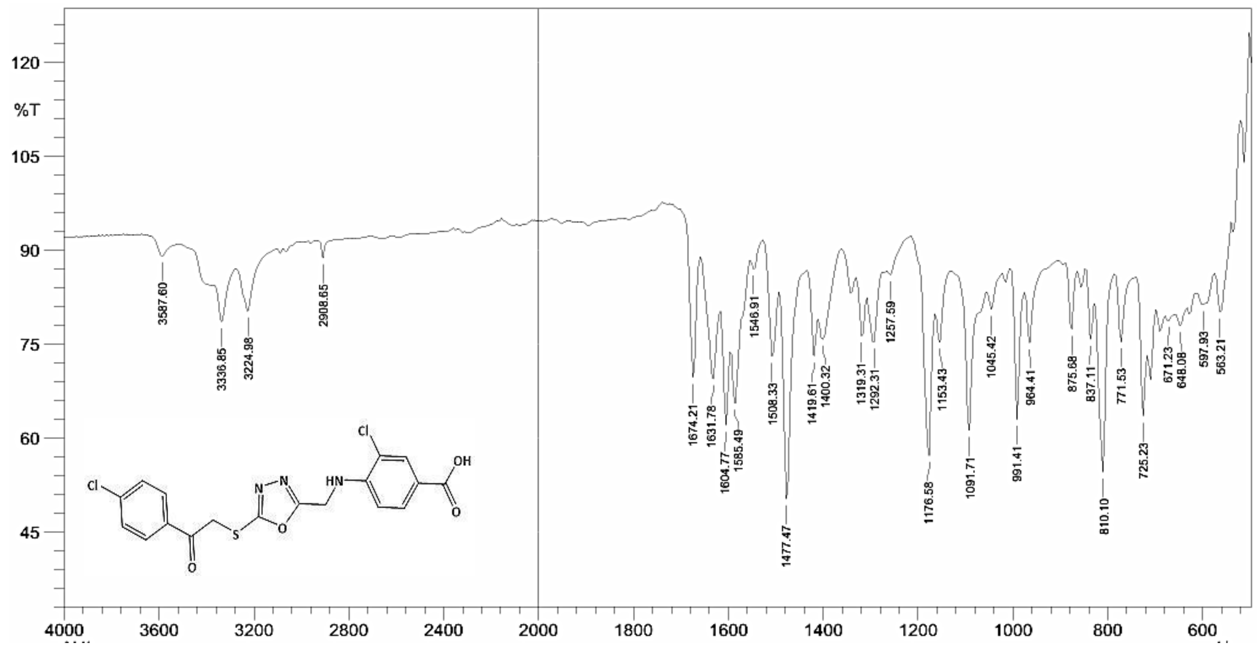

Supplementary Figure 7A. ATR-FTIR spectrum of the Compound A4bn.

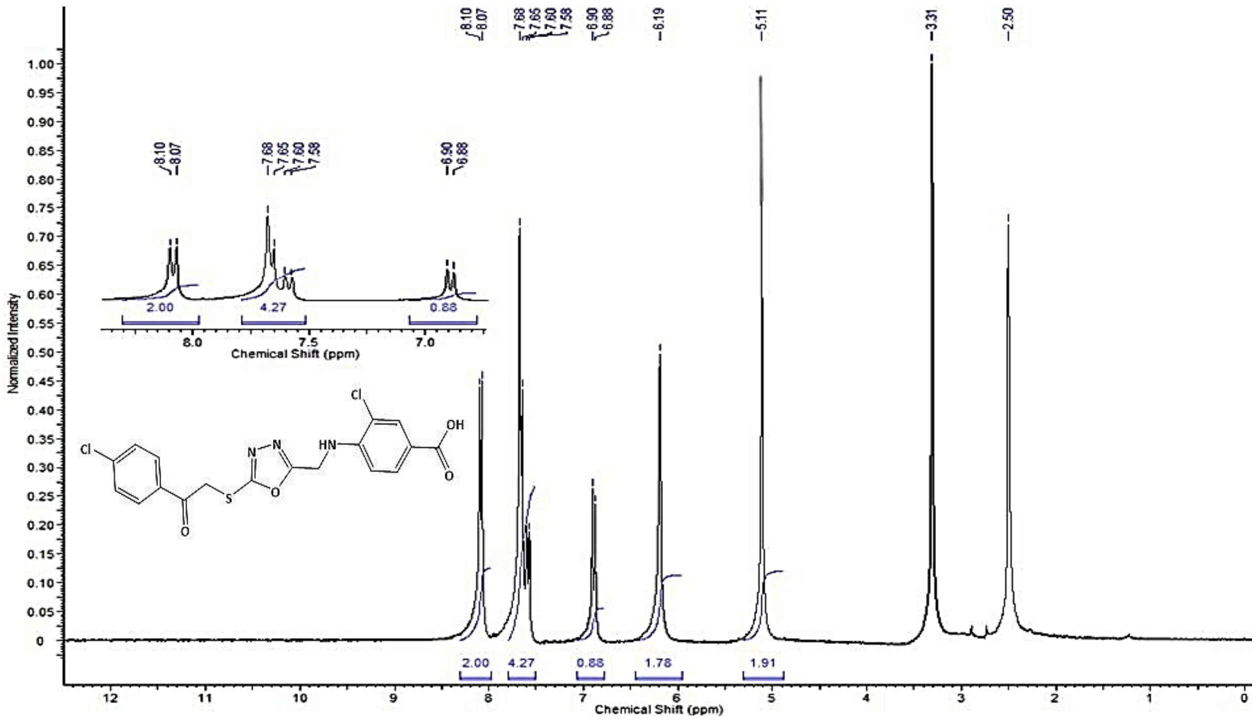

Supplementary Figure 7B. ^{1}HNMR spectrum of the Compound A4bn.

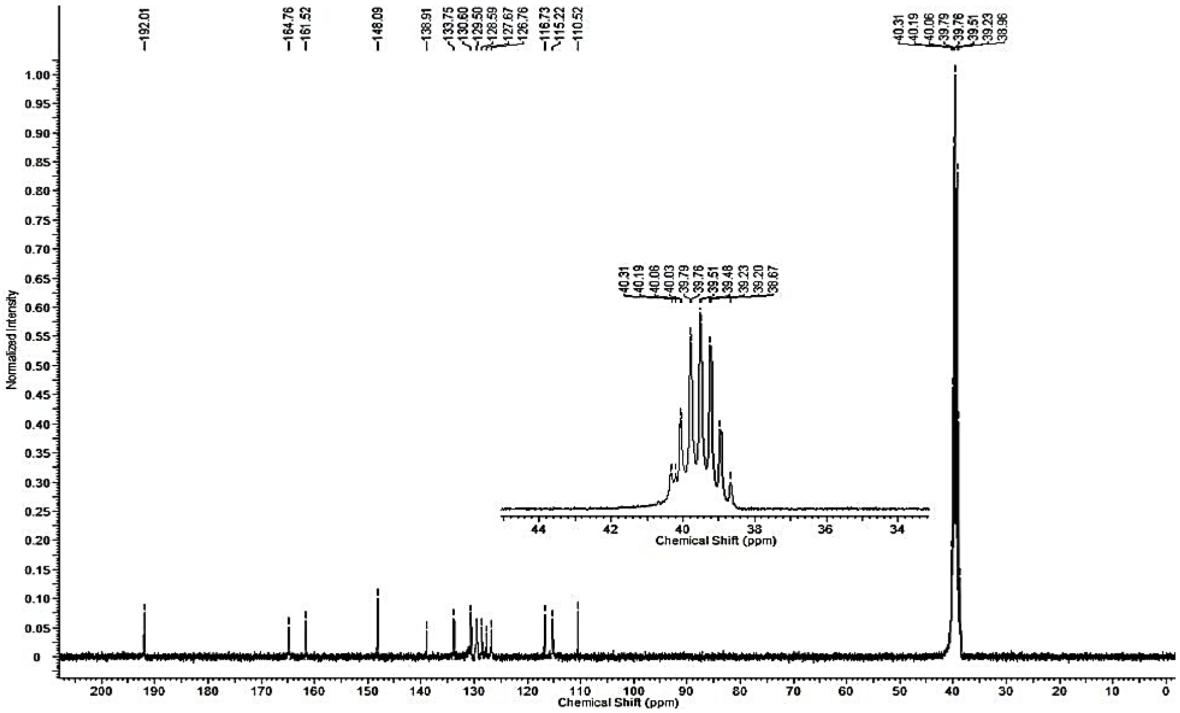

Supplementary Figure 7C. ^{13}CNMR spectrum of the Compound A4bn.

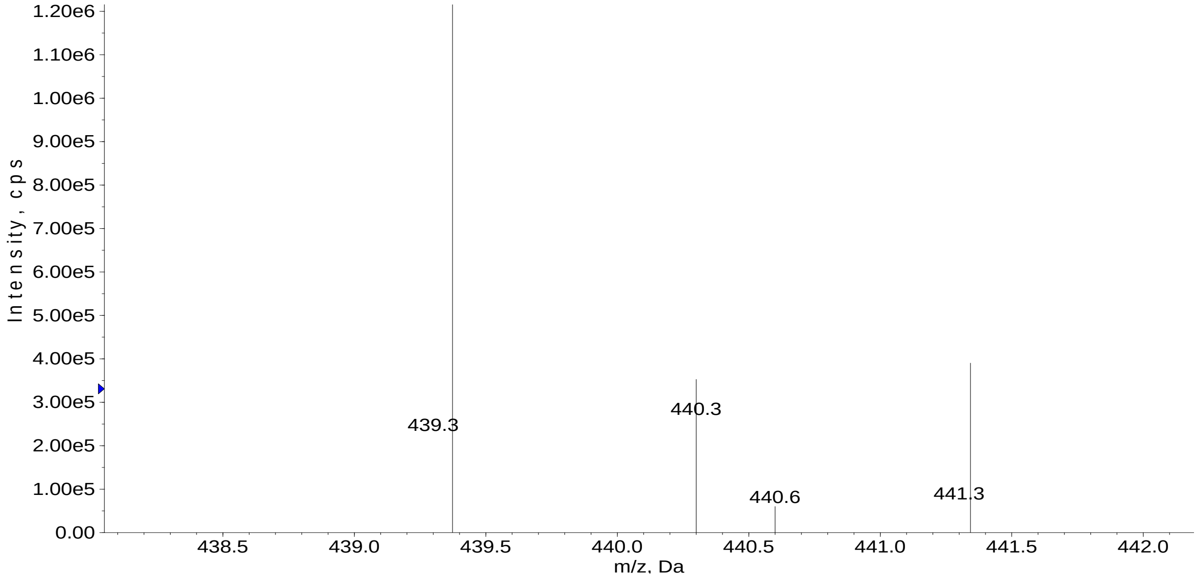

Supplementary Figure 7D. MS spectrum of the Compound A4bn.

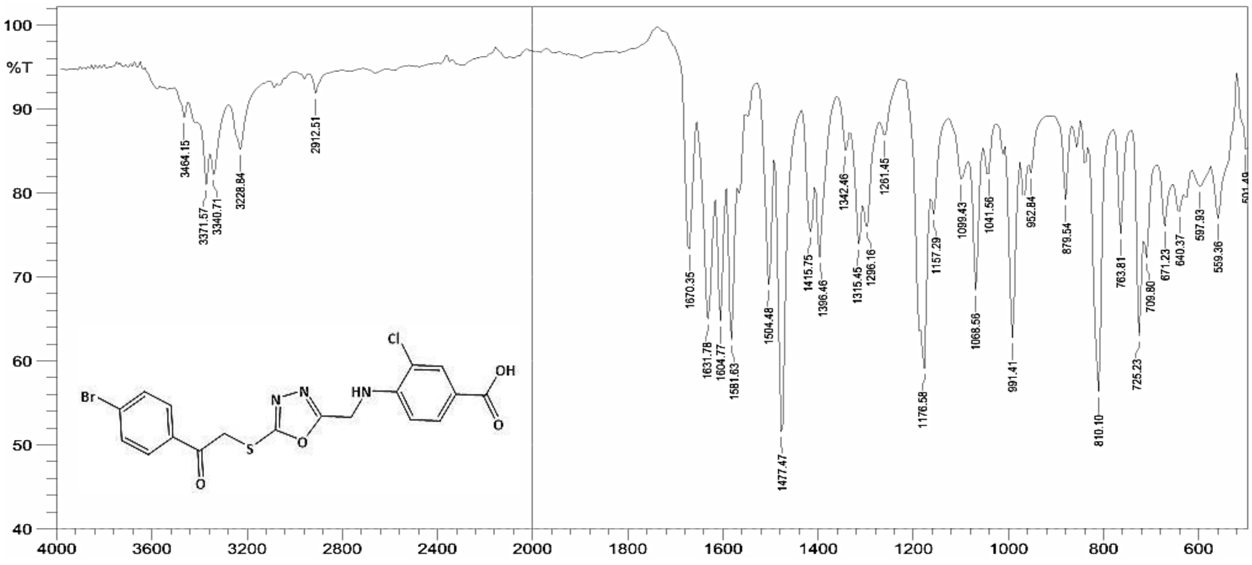

Supplementary Figure 8A. ATR-FTIR spectrum of the Compound A4cn.

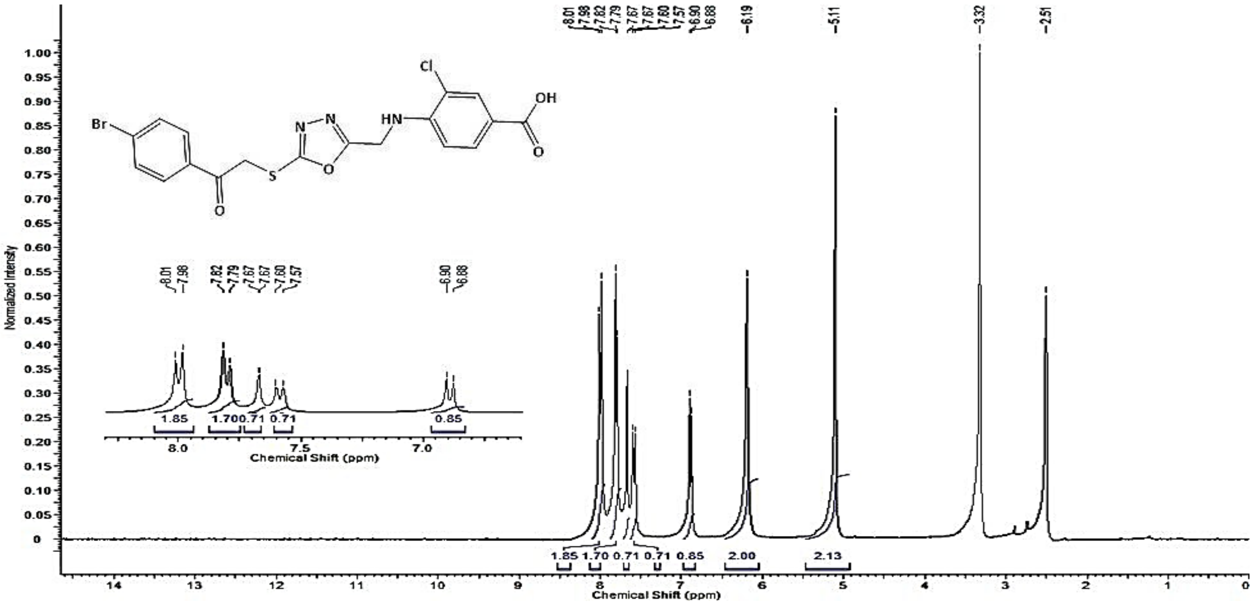

Supplementary Figure 8B. ^{1}HNMR spectrum of the Compound A4cn.

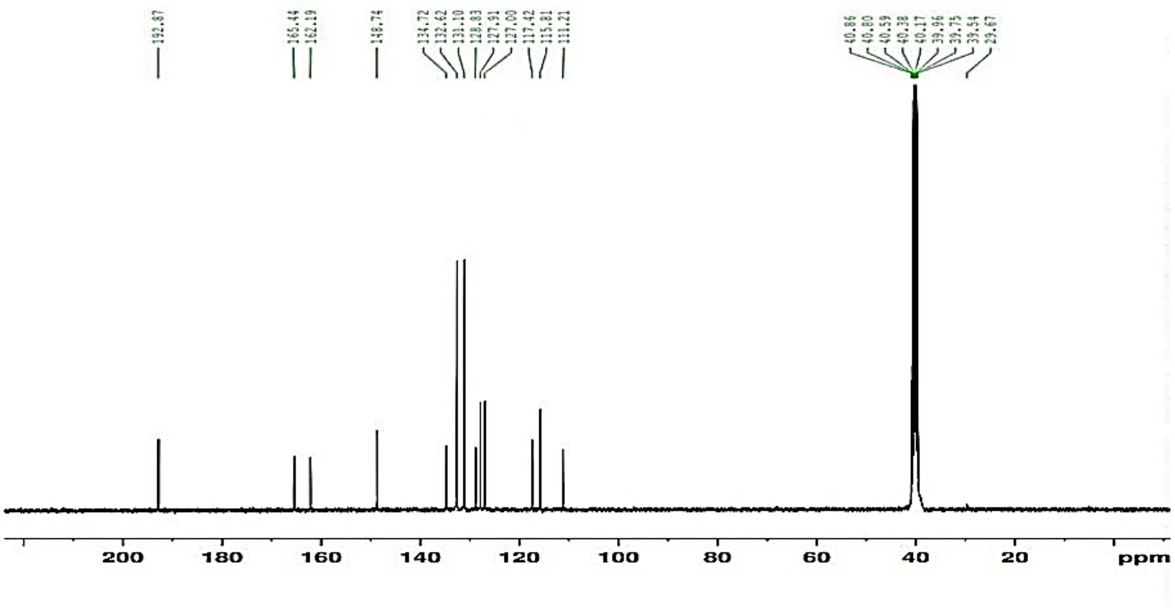

Supplementary Figure 8C. ^{13}CNMR spectrum of the Compound A4cn.

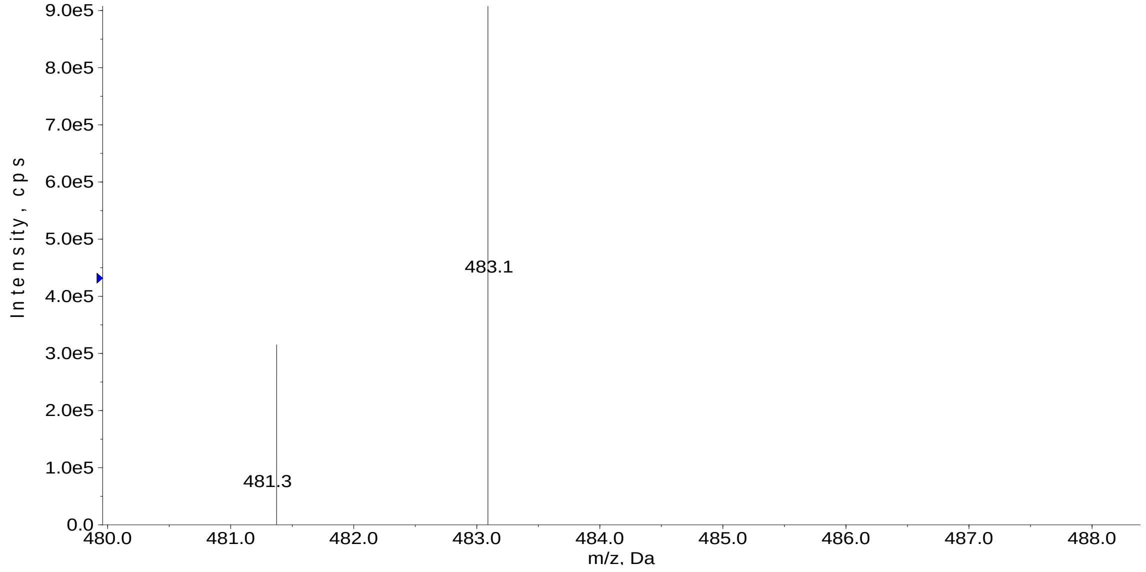

Supplementary Figure 8D. MS spectrum of the Compound A4cn.

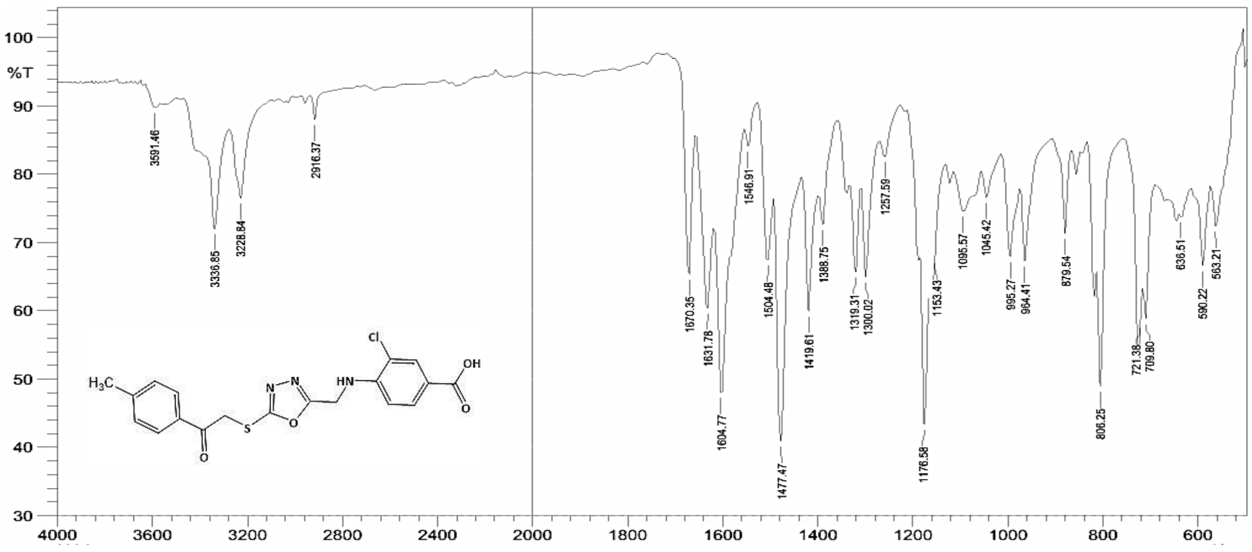

Supplementary Figure 9A. ATR-FTIR spectrum of the Compound A4dn.

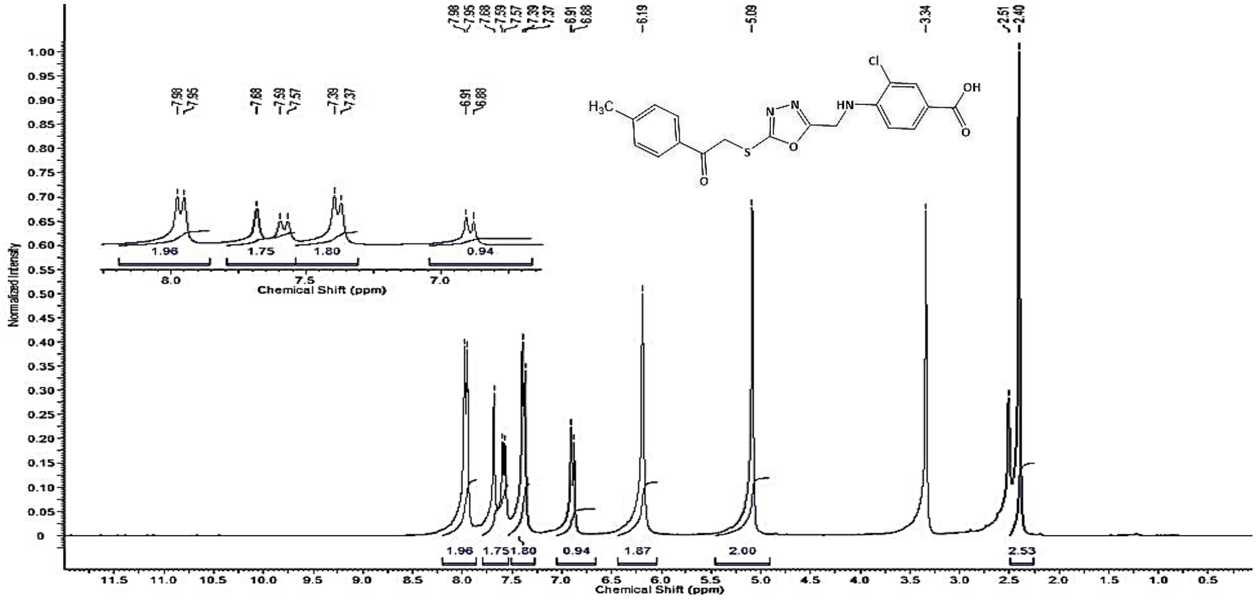

Supplementary Figure 9B. ^{1}HNMR spectrum of the Compound A4dn.

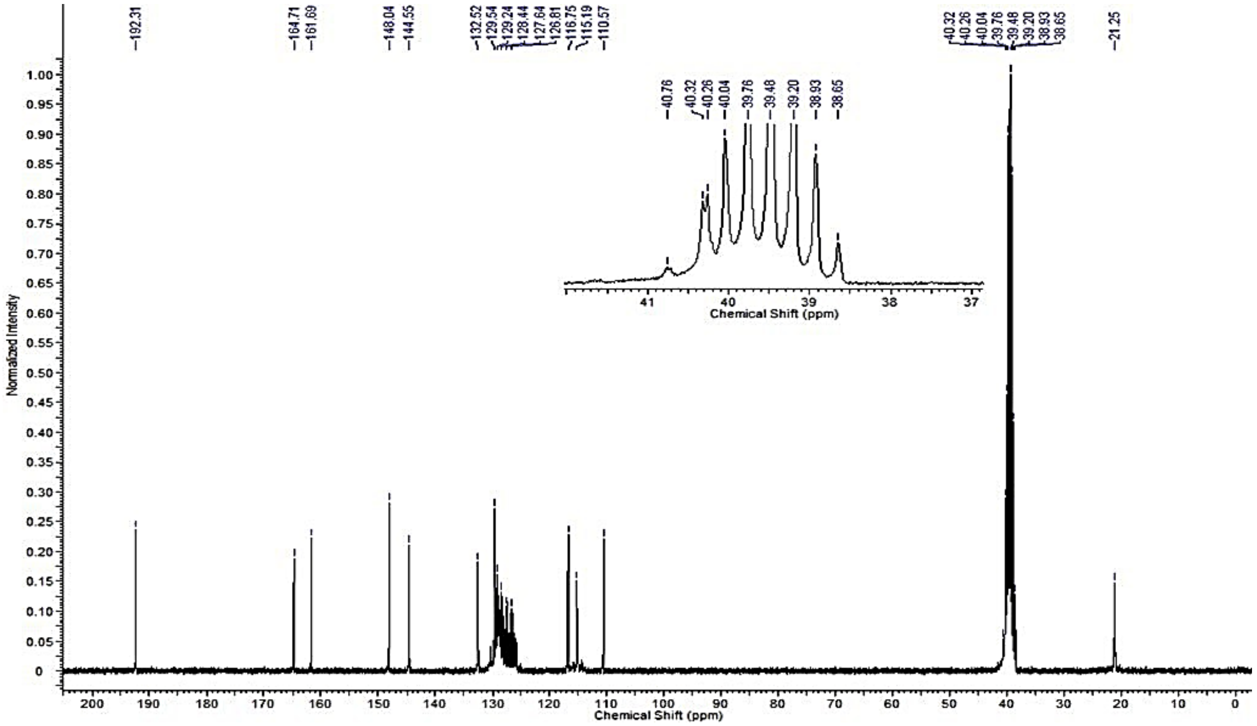

Supplementary Figure 9C. ^{13}CNMR spectrum of the Compound A4dn.

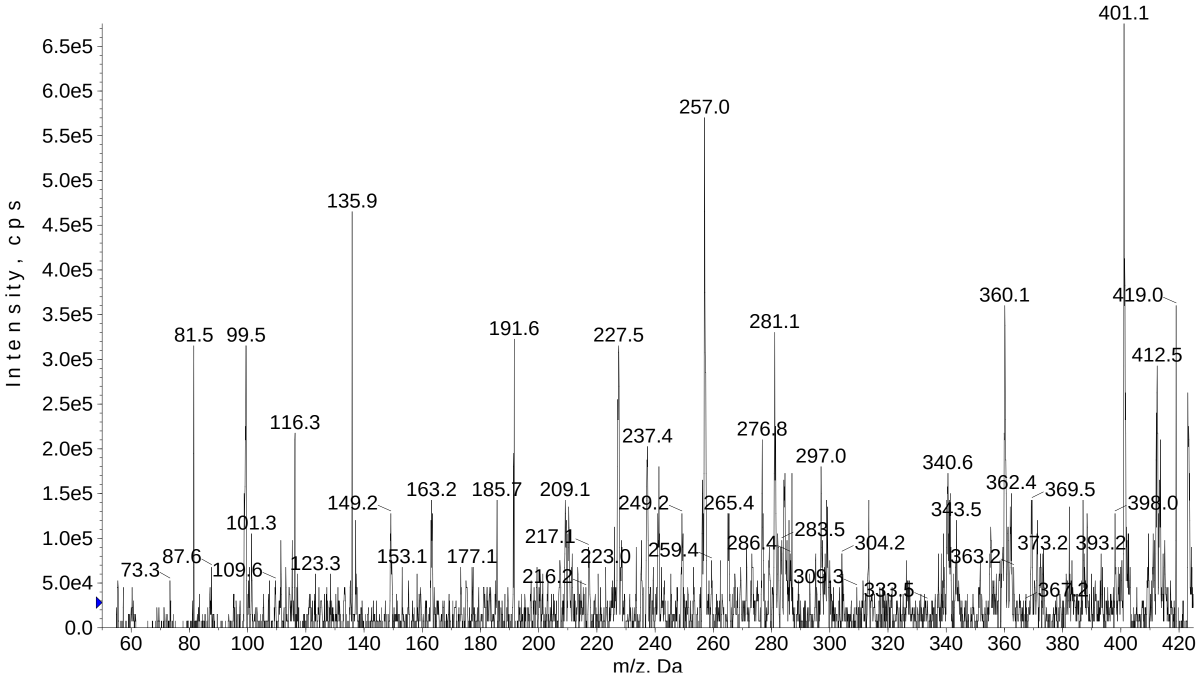

Supplementary Figure 9D. MS spectrum of the Compound A4dn.

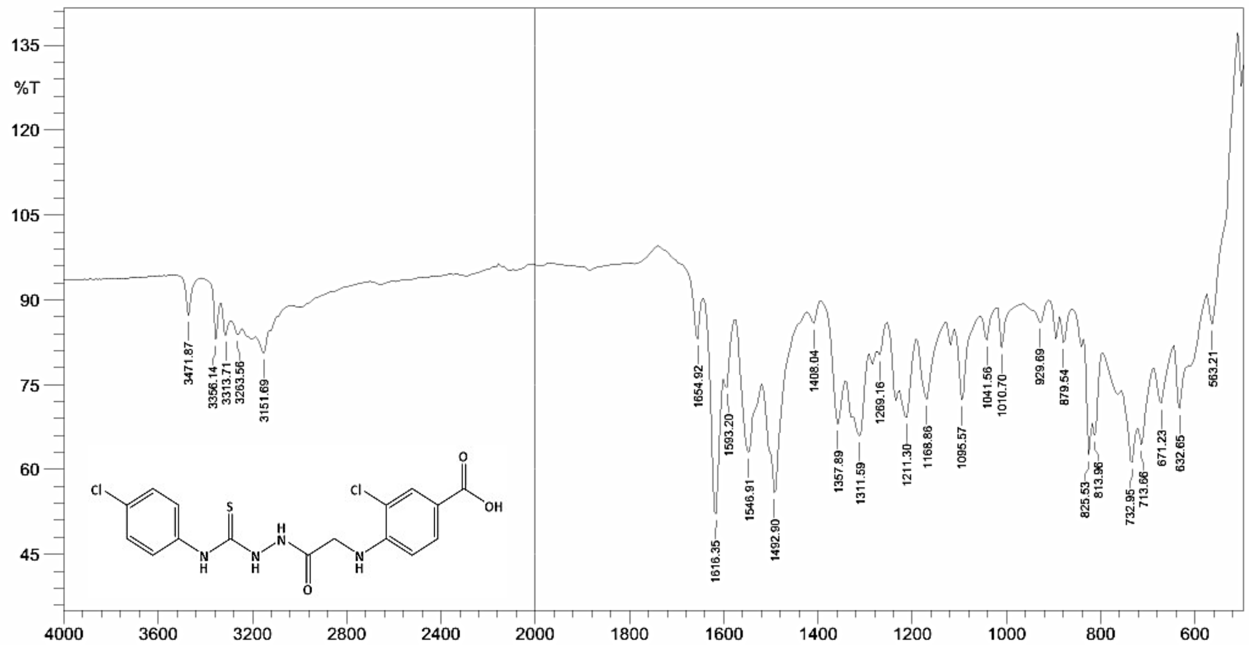

Supplementary Figure 10A. ATR-FTIR spectrum of the compound A5an.

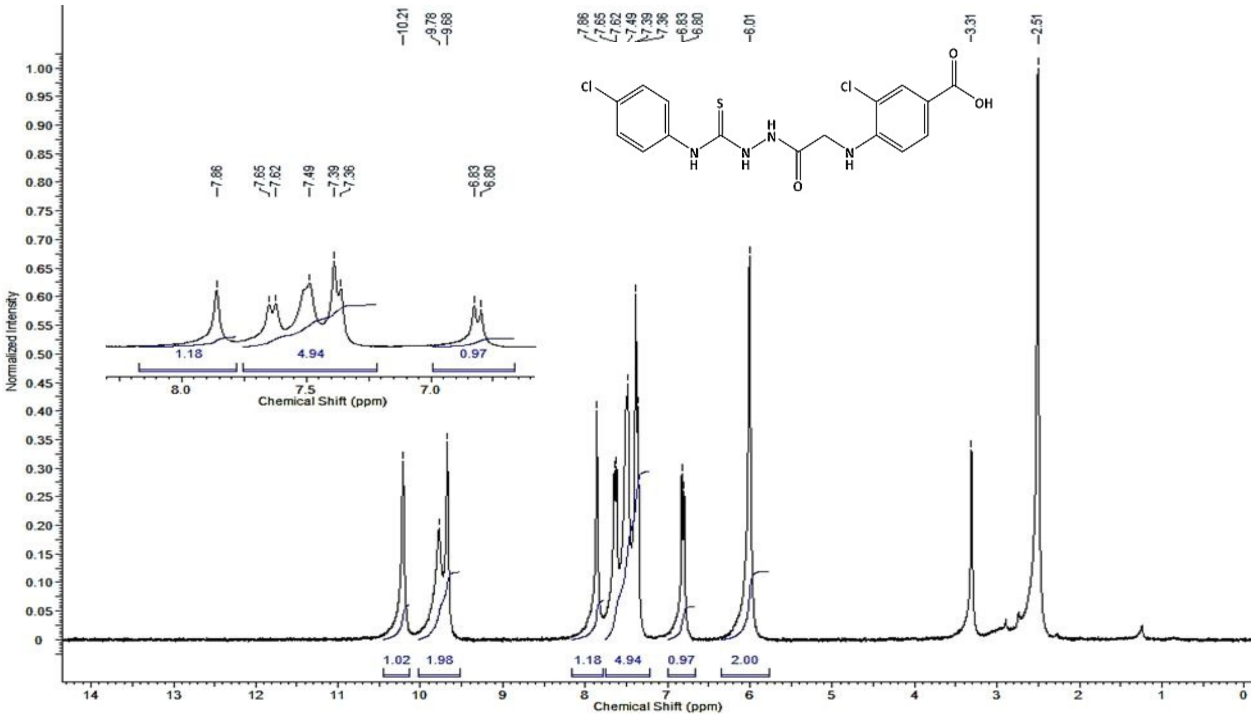

Supplementary Figure 10B. ^{1}HNMR spectrum of the Compound A5an.

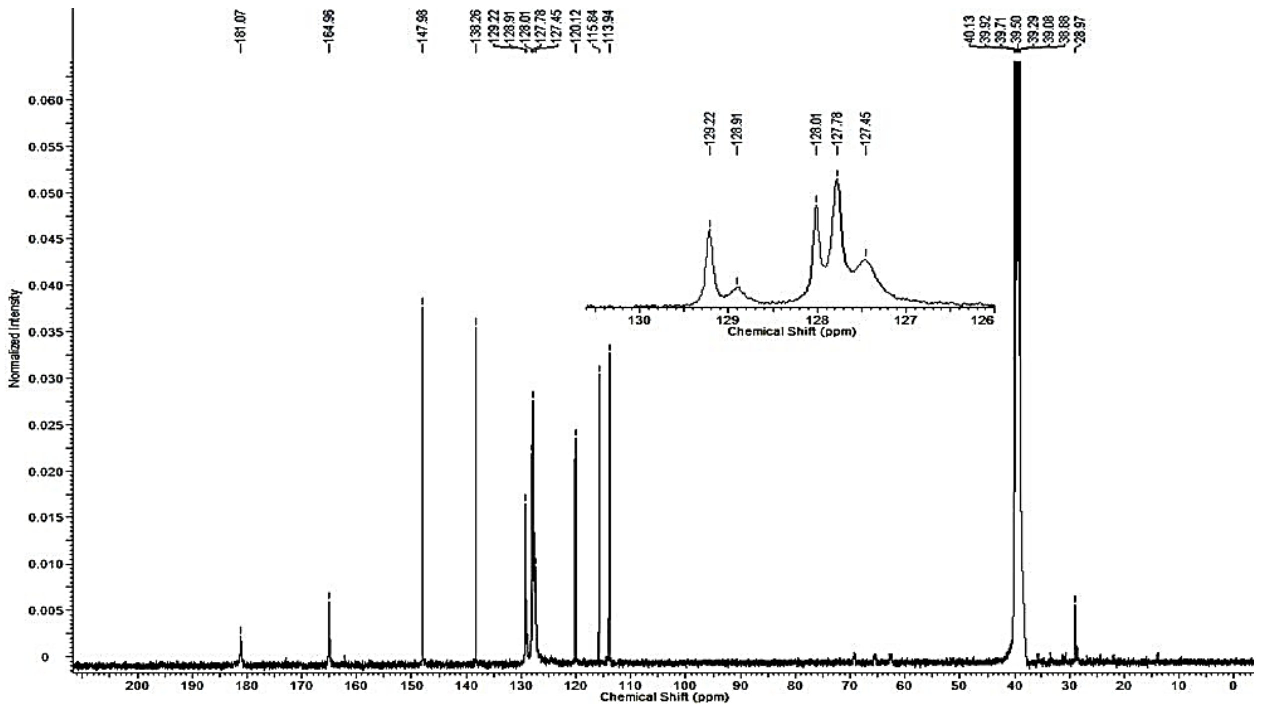

Supplementary Figure 10C. ^{13}CNMR spectrum of the Compound A5an.

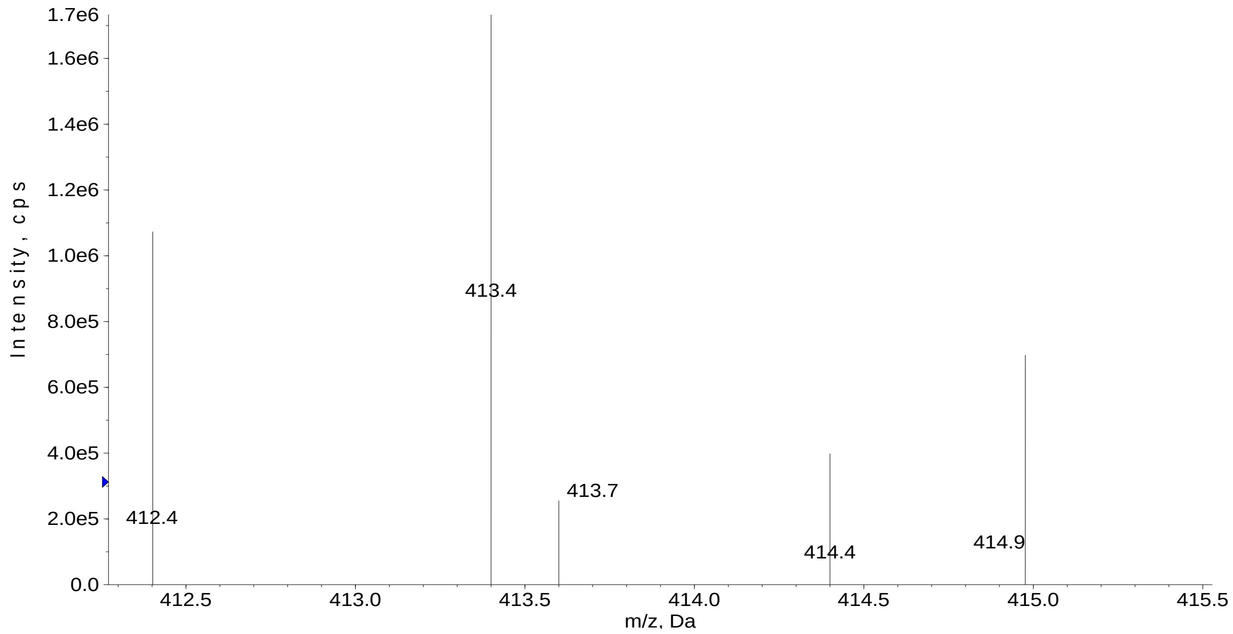

Supplementary Figure 10D. MS spectrum of the Compound A5an.

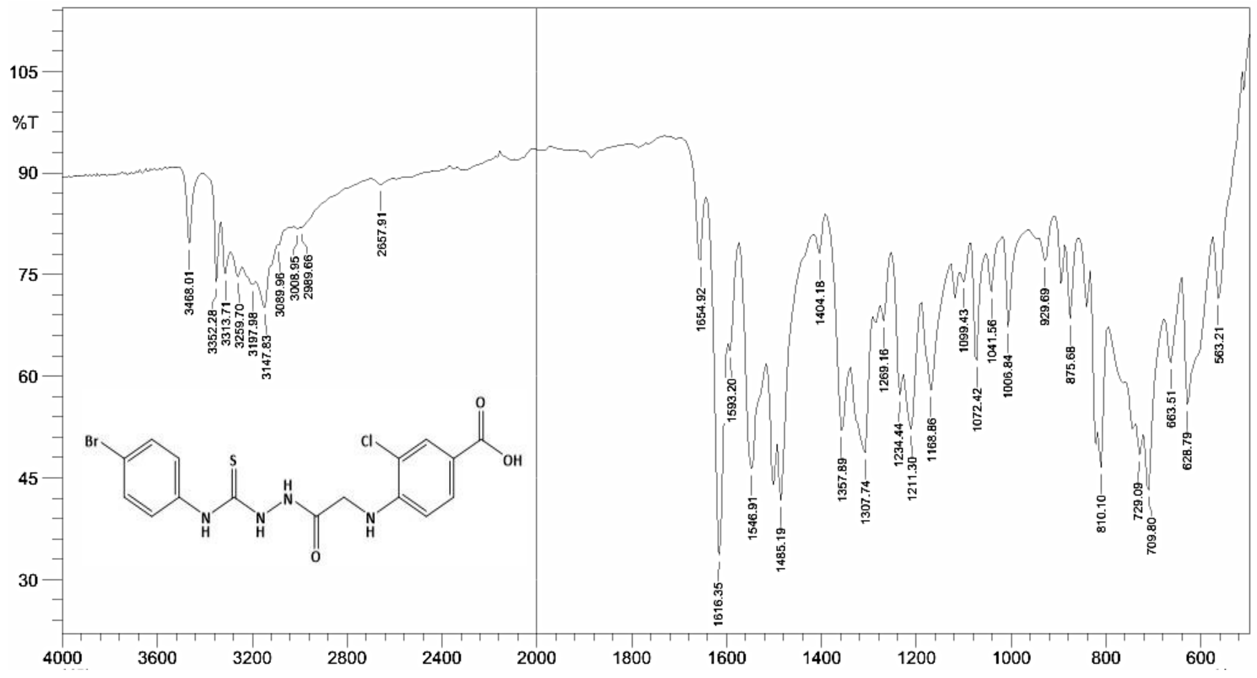

Supplementary Figure 11A. ATR-FTIR spectrum of the Compound A5bn.

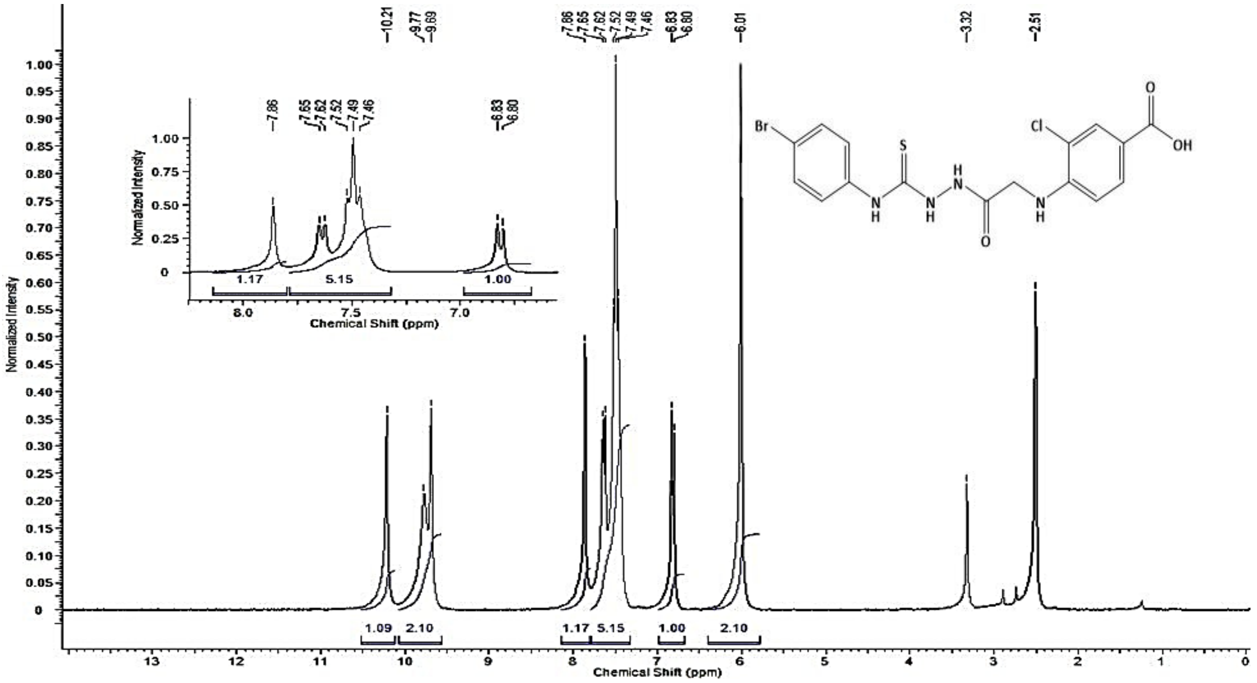

Supplementary Figure 11B. ^{1}HNMR spectrum of the Compound A5bn.

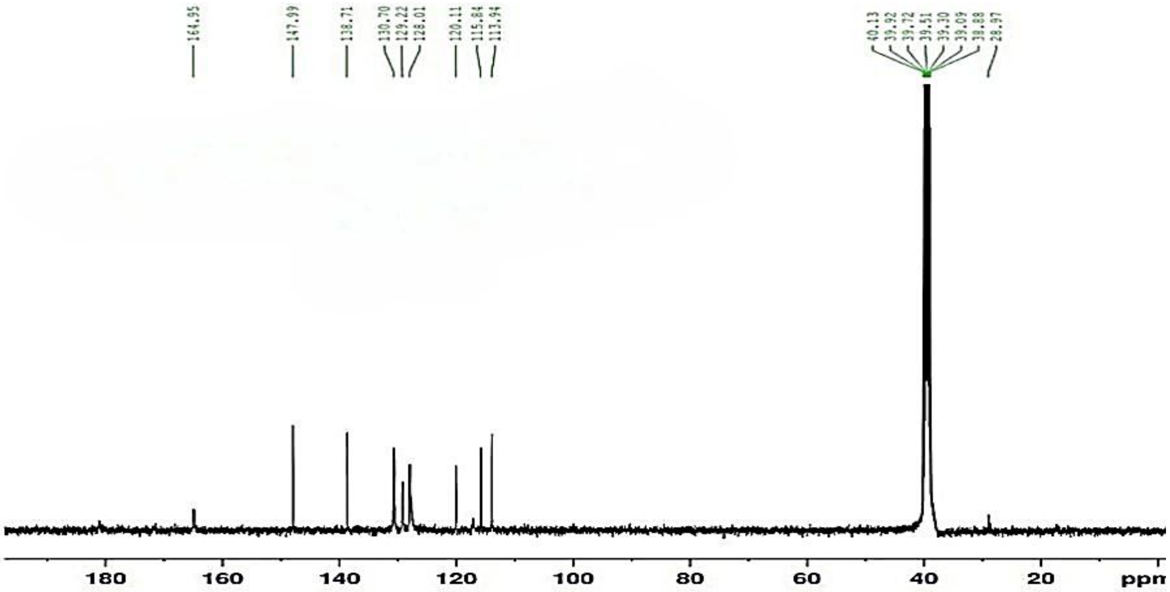

Supplementary Figure 11C. ^{13}CNMR spectrum of the Compound A5bn.

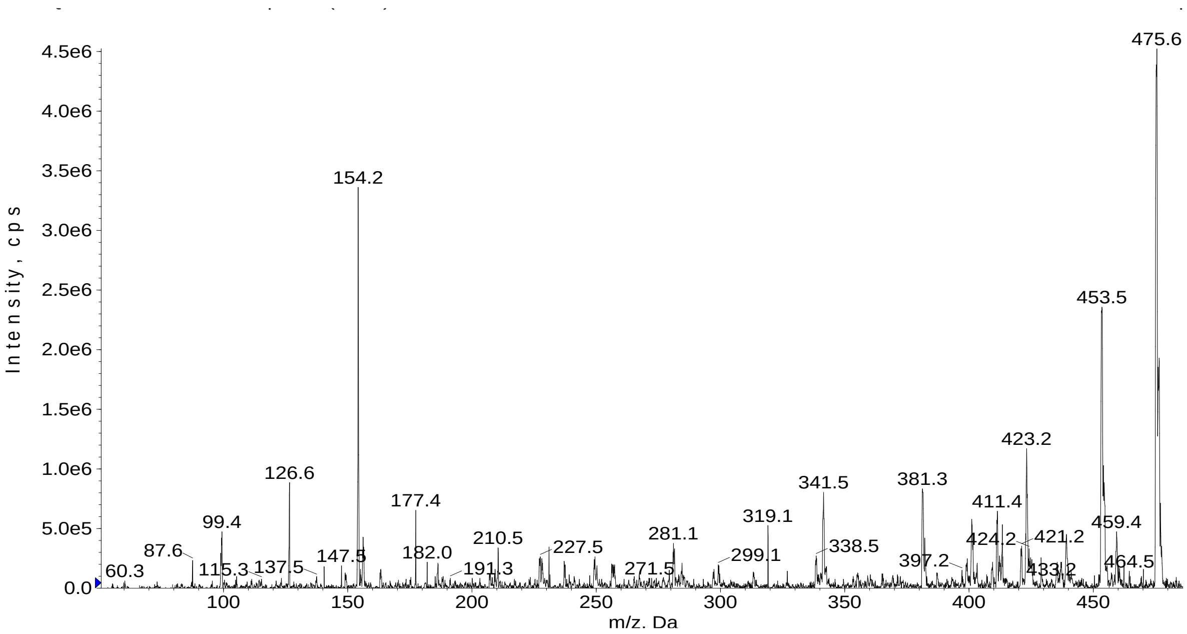

Supplementary Figure 11D. MS spectrum of the Compound A5bn.

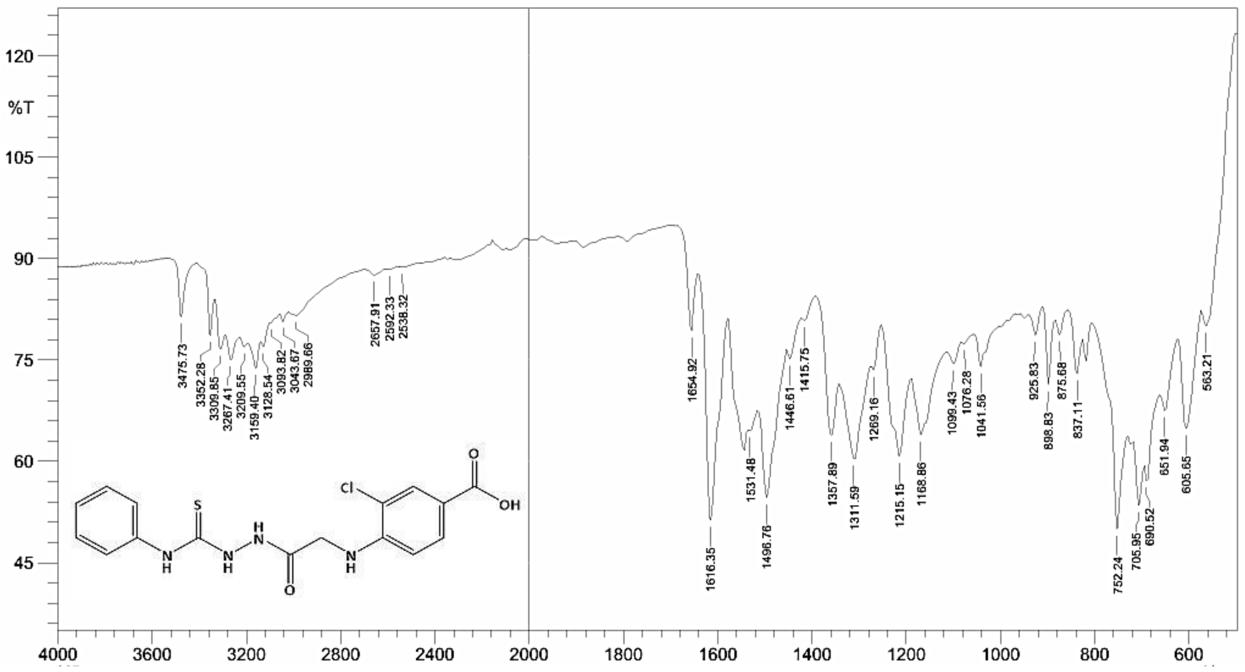

Supplementary Figure 12A. ATR-FTIR spectrum of the Compound A5cn.

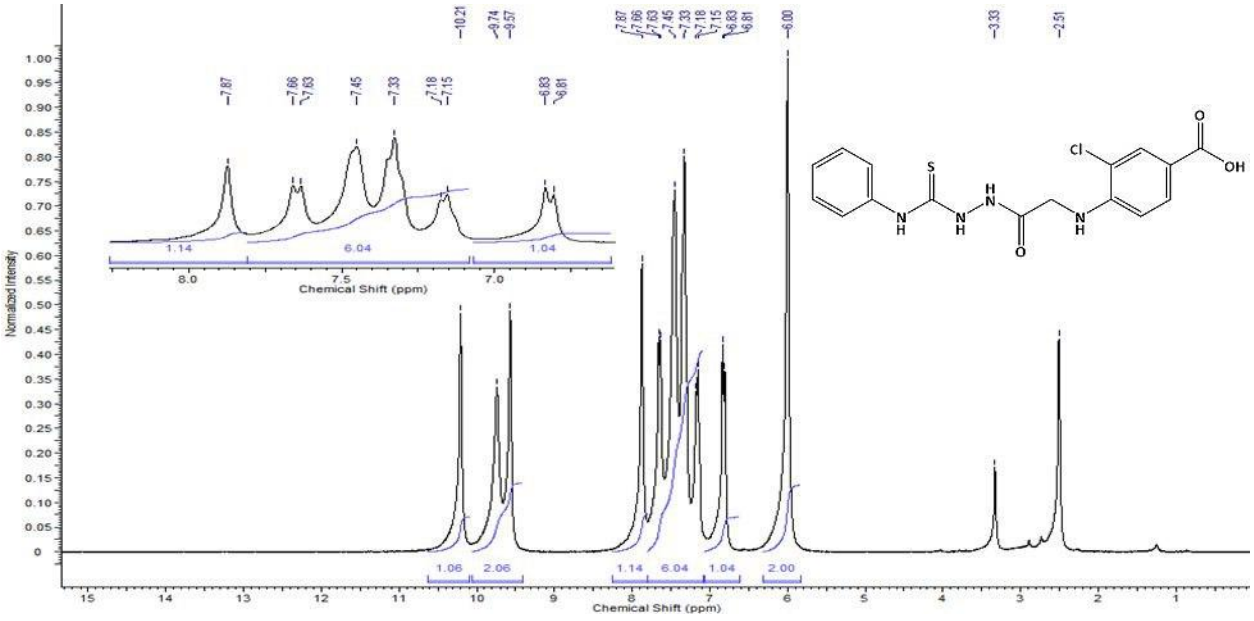

Supplementary Figure 12B. ^{1}HNMR spectrum of the Compound A5cn.

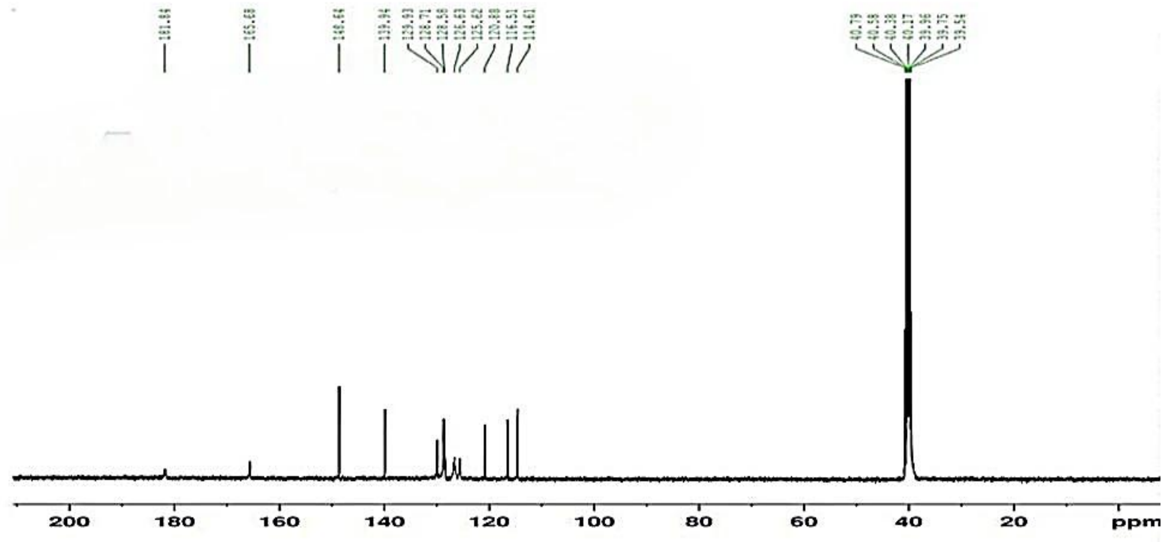

Supplementary Figure 12C. ^{13}CNMR spectrum of the Compound A5cn.

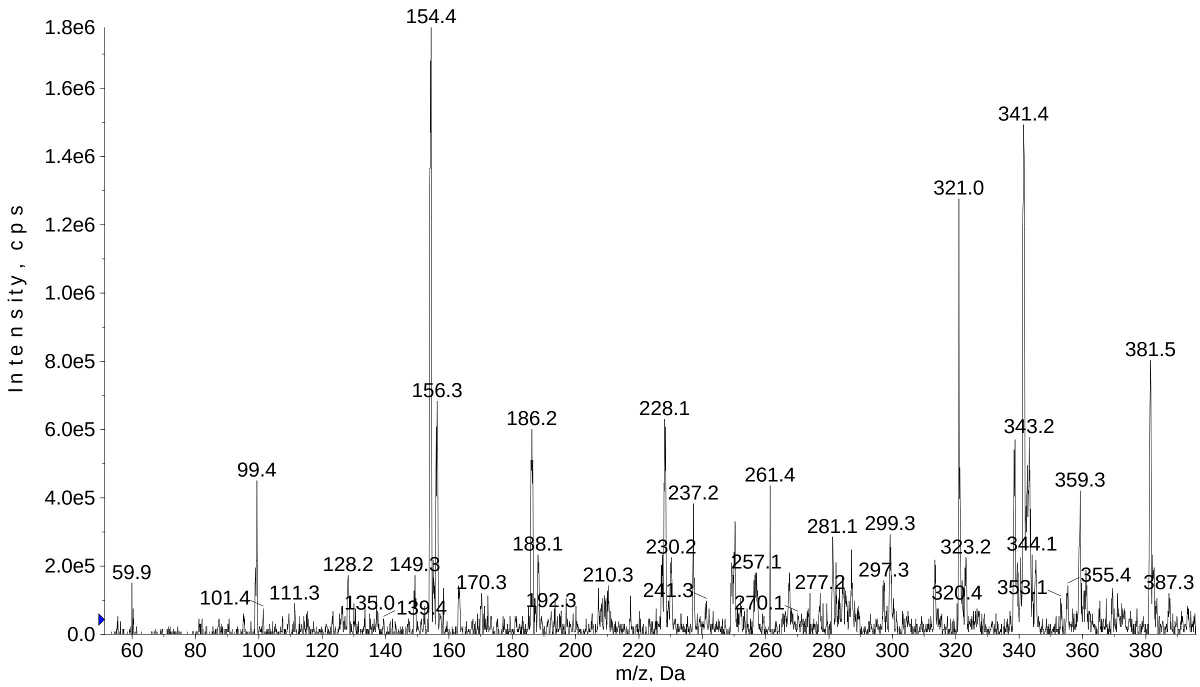

Supplementary Figure 12D. MS spectrum of the Compound A5cn.

A)

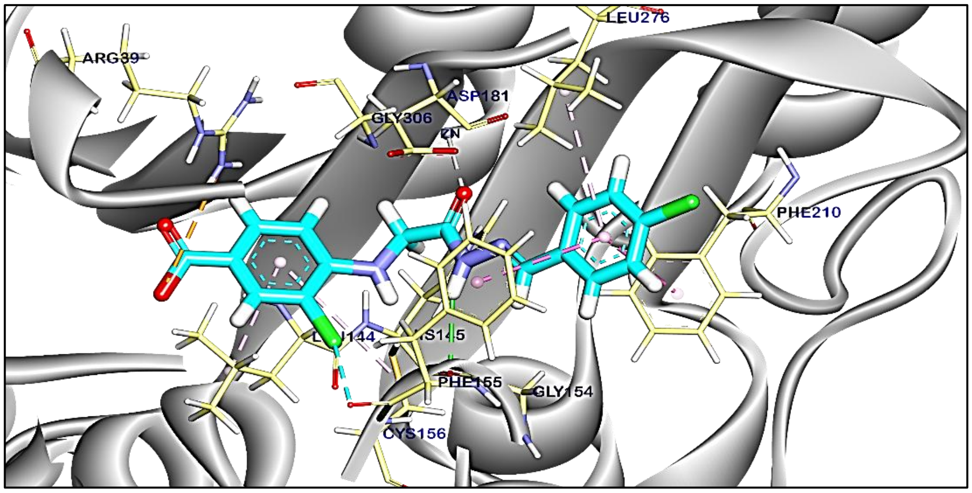
LEU276
ARG39
ASP181
GLY306
ZN
PHE210
PHE155
GLY154
CYS156

B)

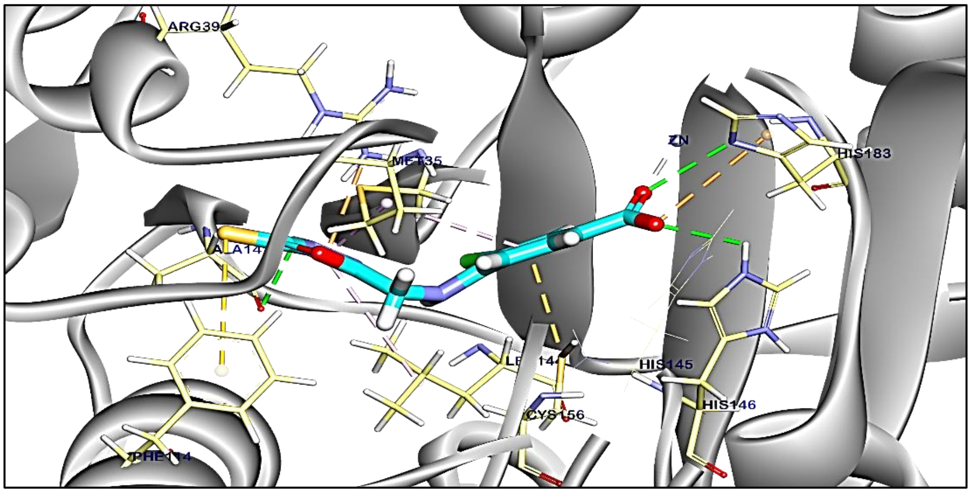
ARG39
ZN
HIS183
HIS145
HIS146
CYS156

C)

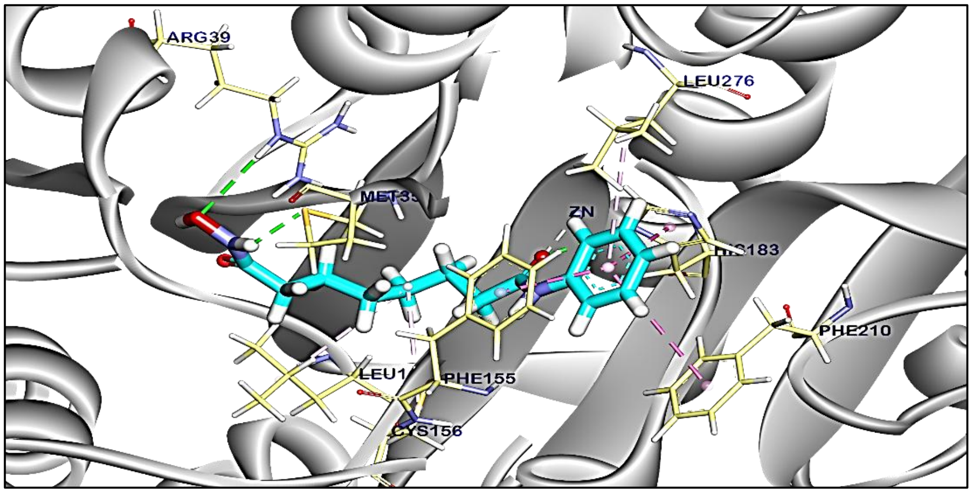

D)

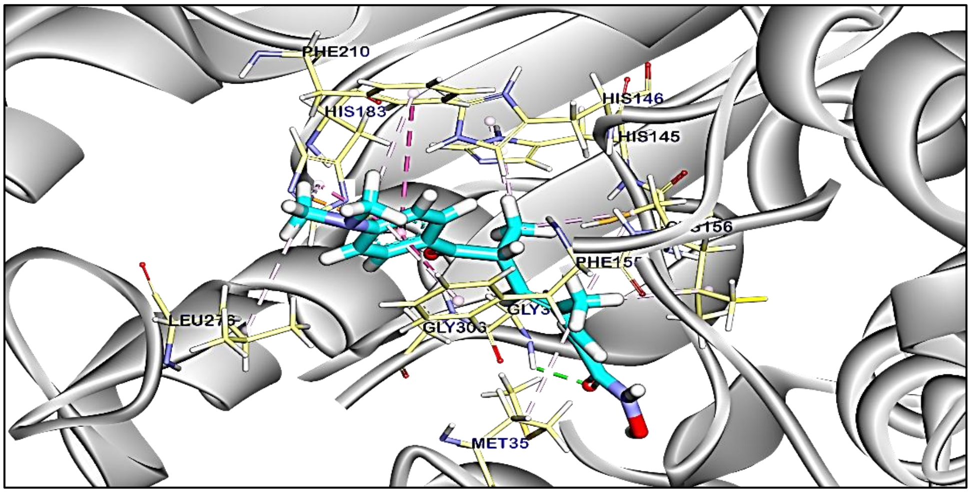

Supplementary Figure 13. 3D mapping of the proposed binding mode of A) Compound A3an, B) Compound A4, C) The reference ligand SAHA, D) The reference ligand trichostatin against HDAC2.

A)

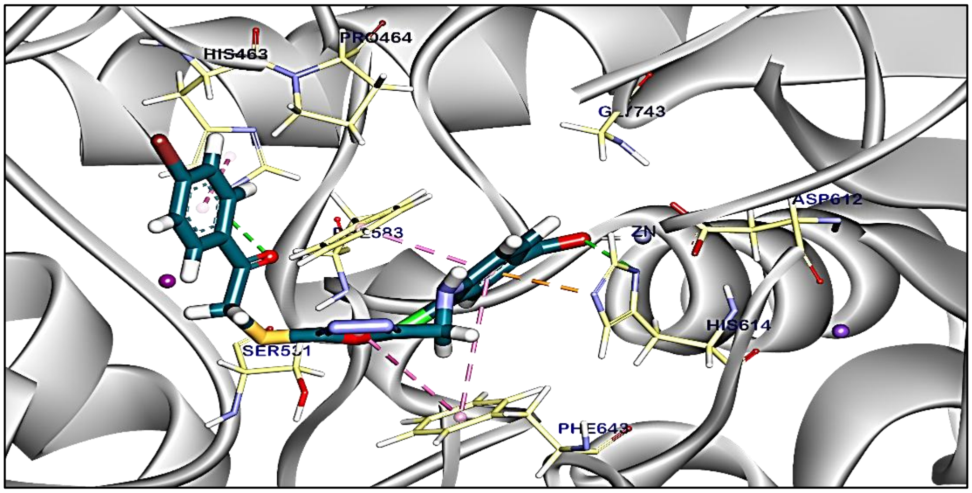
HIS463
PRO464
GLY743
ASP612
ZN
SER511
HIS614
PHE643

B)

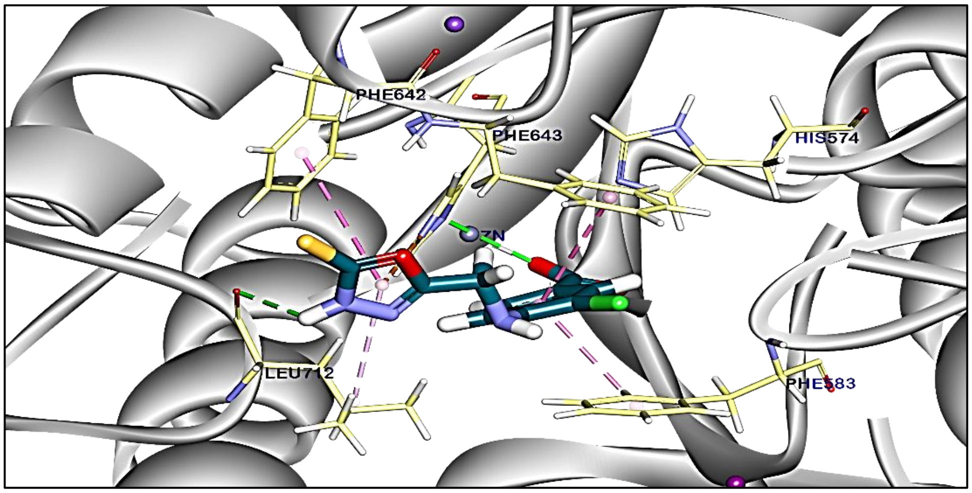
PHE642
PHE643
HIS574
ZN
LEU712
PHE583

C)

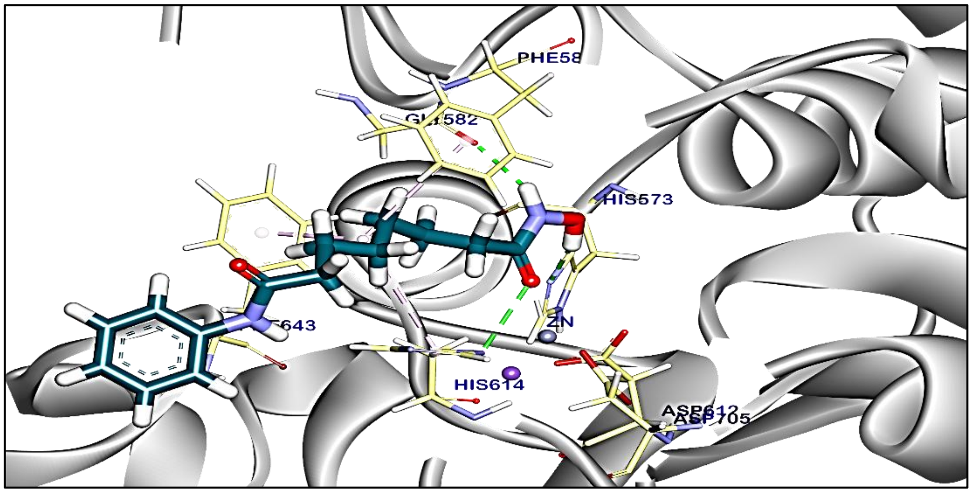

D)

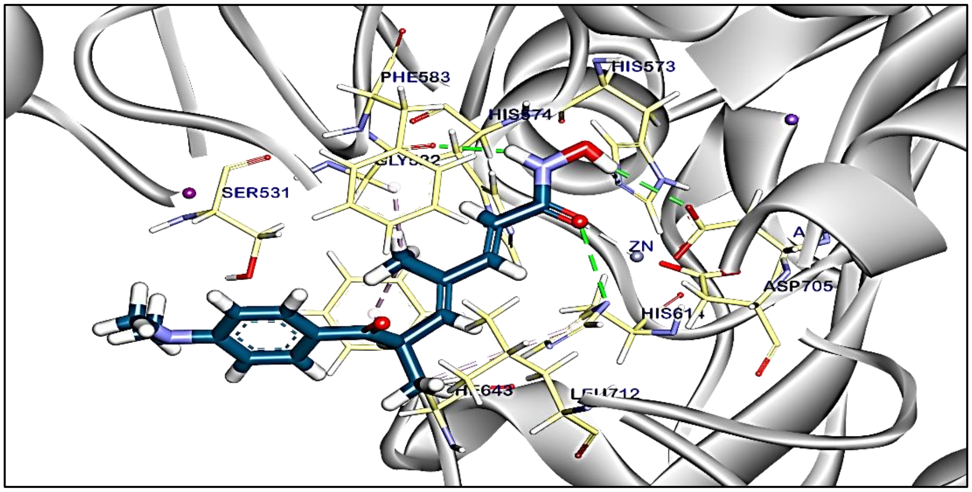

Supplementary Figure 14. 3D mapping of the proposed binding mode of A) Compound A4cn, B) Compound A4, C) The reference ligand SAHA, D) The reference ligand trichostatin against HDAC6.

A)

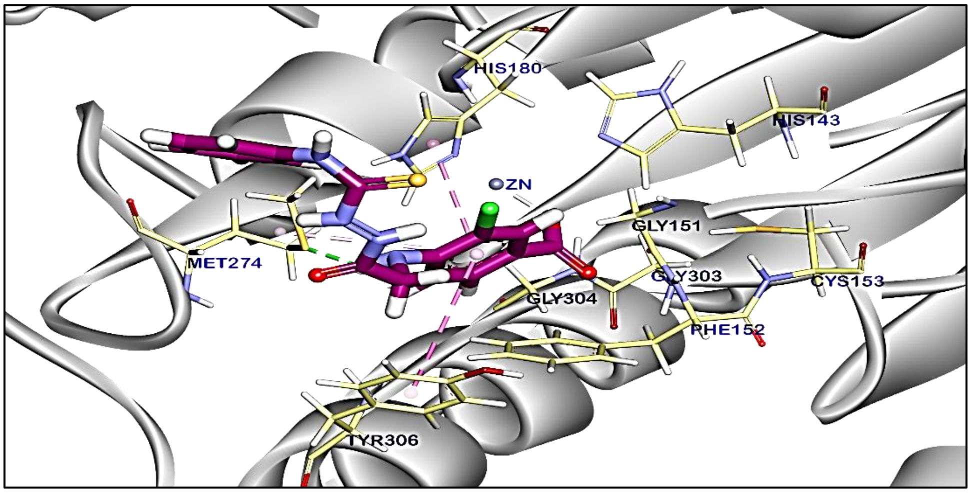
HIS180
HIS143
ZN
GLY151
MET274
GLY303
CYS153
GLY304
PHE152
TYR306

B)

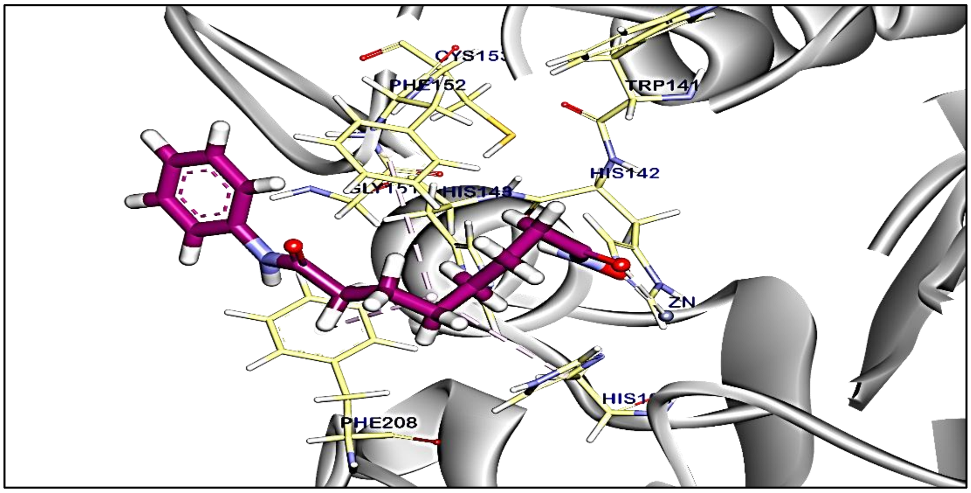
PHE152
TRP141
HIS142
ZN
PHE208

C)

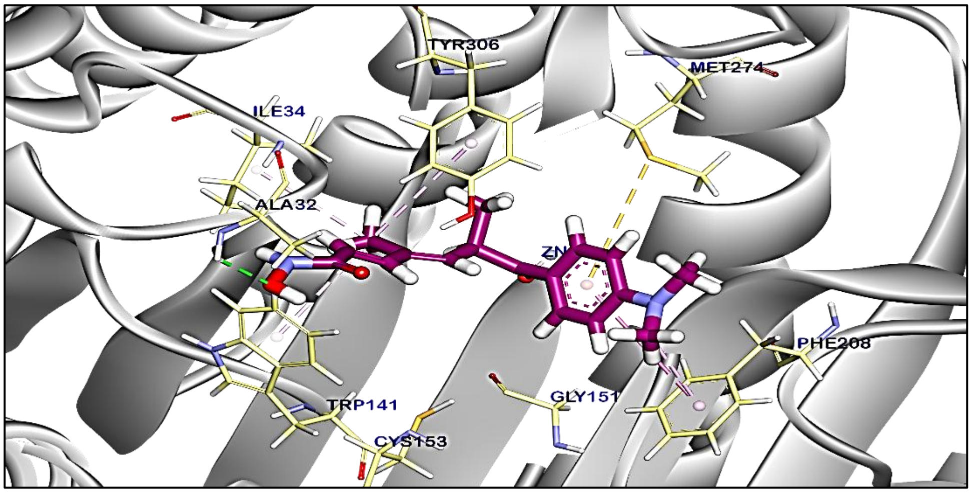

Supplementary Figure 15. 3D mapping of the proposed binding mode of A) Compound A5cn, B) The reference ligand SAHA, C) The reference ligand trichostatin against HDAC8.

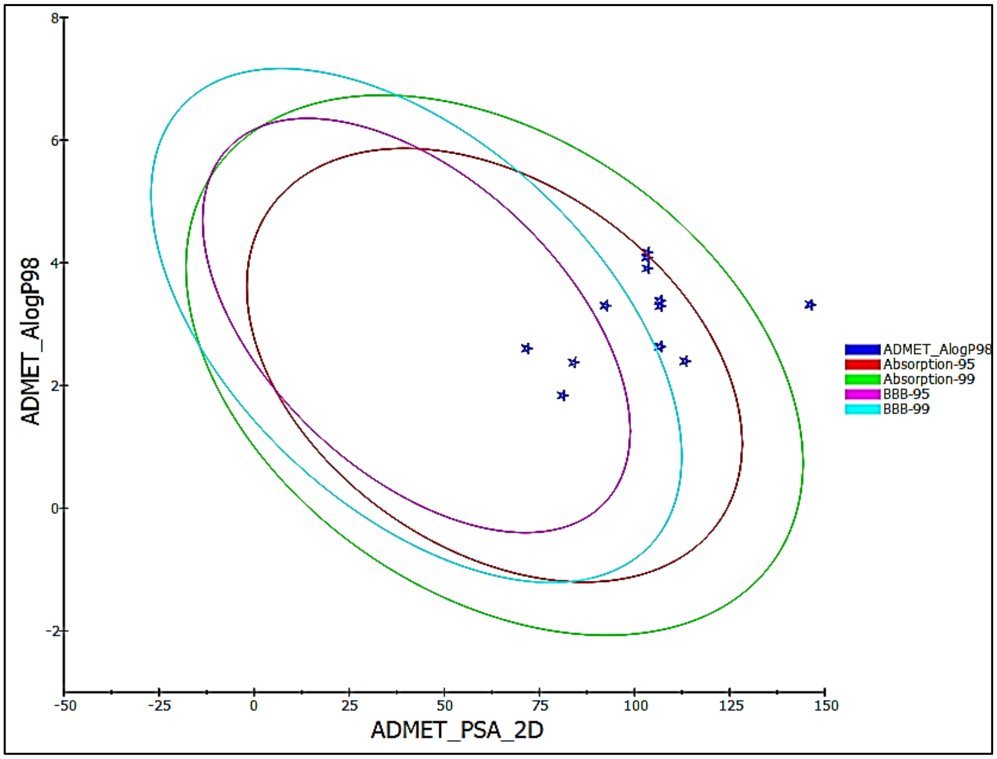

Supplementary Figure 16. Predicted ADMET descriptors for the synthesized compounds.

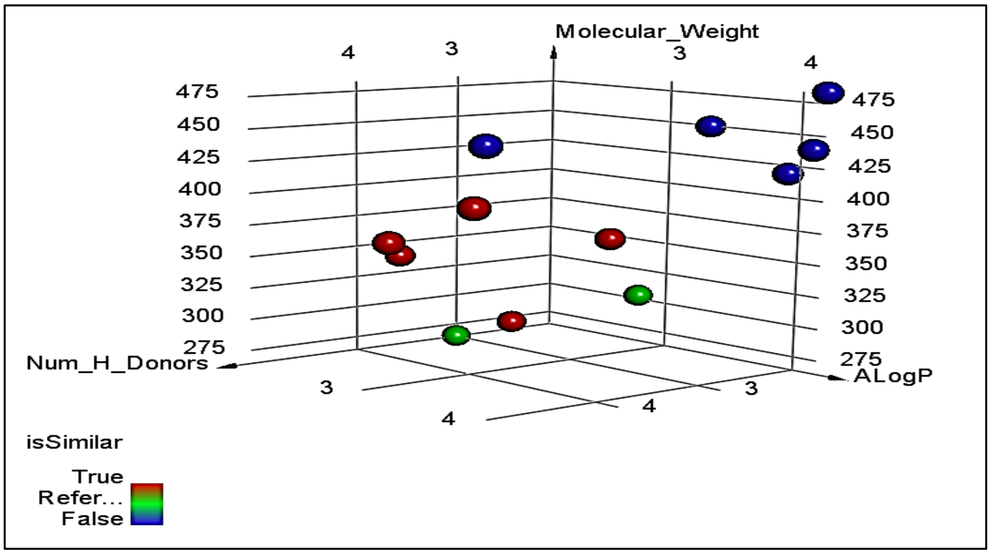

Supplementary Figure 17. Molecular similarity analysis between the tested compounds and the reference HDACi SAHA and trichostatin. Green ball = reference ligands (reference), red balls = similar ligands (A3an, A3bn, A5cn, A5an, and A4), blue balls (A5bn, A4dn, A4bn, A4cn, and A4an)= not similar ligands.

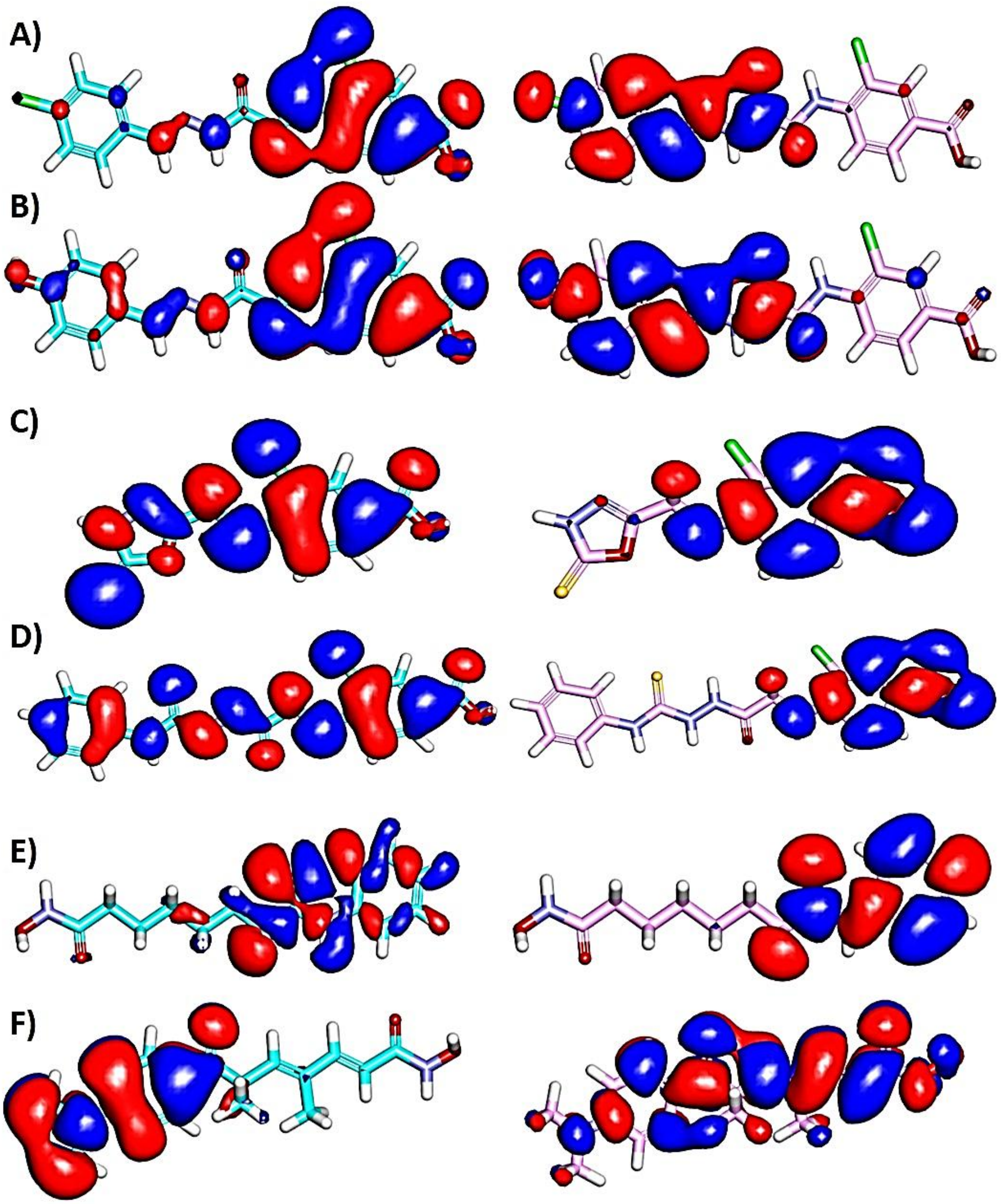

Supplementary Figure 18. HOMO and LUMO orbitals of selected compounds. A) Compound A3an, B) Compound A3bn, C) Compound A4, D) Compound A5cn, E) SAHA, F) Trichostatin.

A)

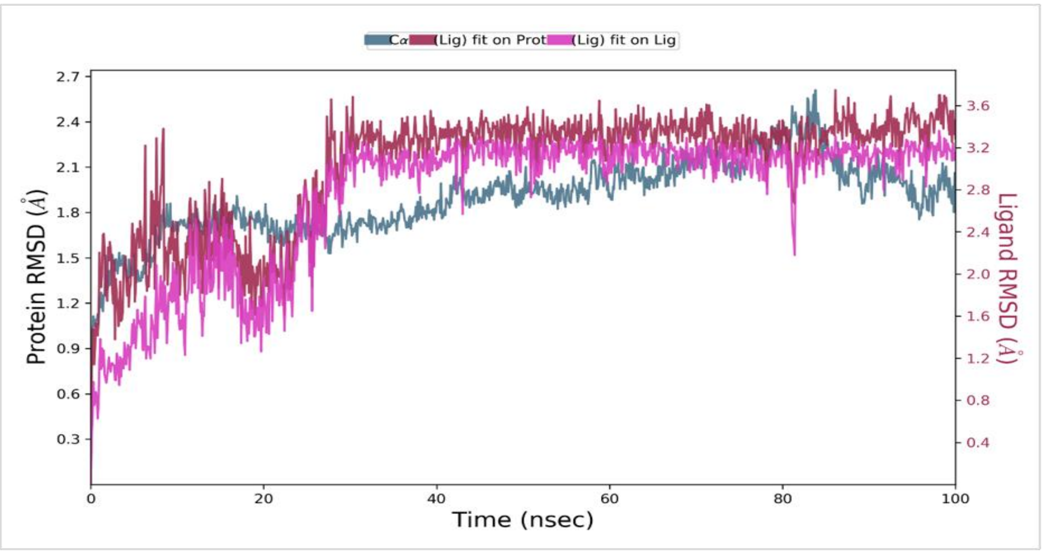

B)

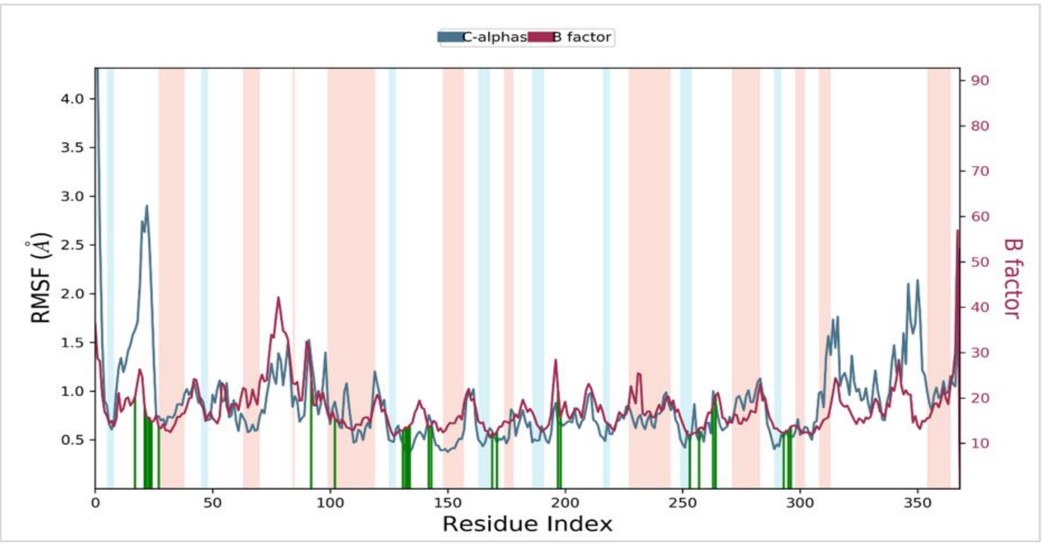

Supplementary Figure 19. RMSD and RMSF of compound A3bn against HDAC2 during the 100 ns simulation period.

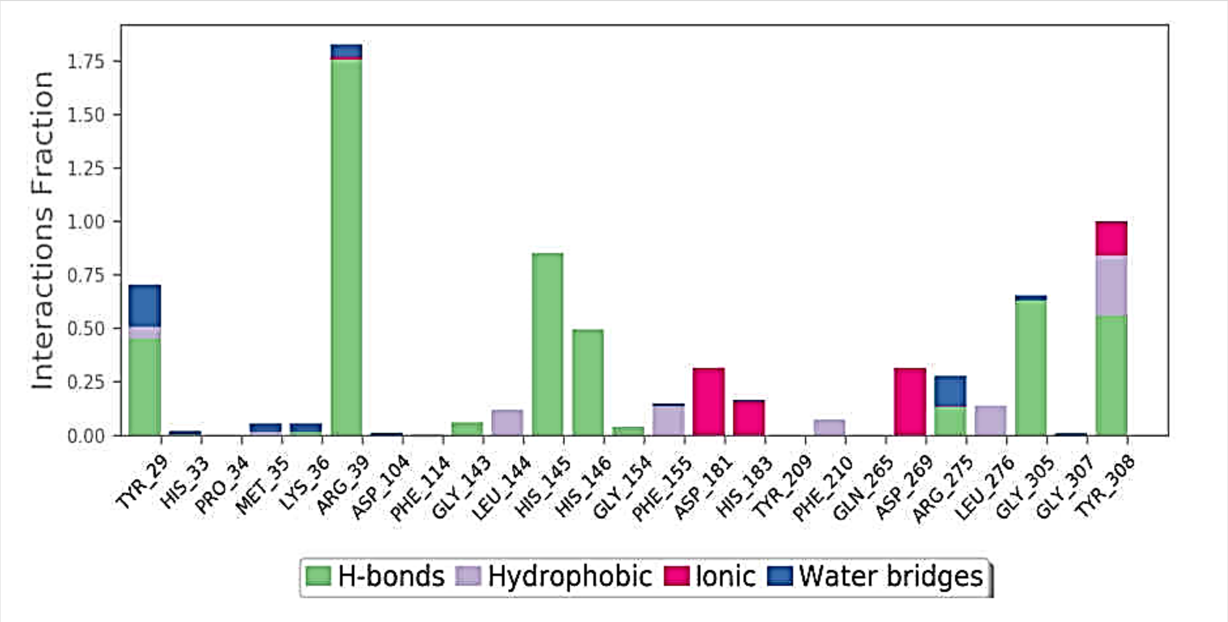

Supplementary Figure 20. Histogram analysis describing the binding interactions of compound A3bn with HDAC2 during the simulation period (100 ns).

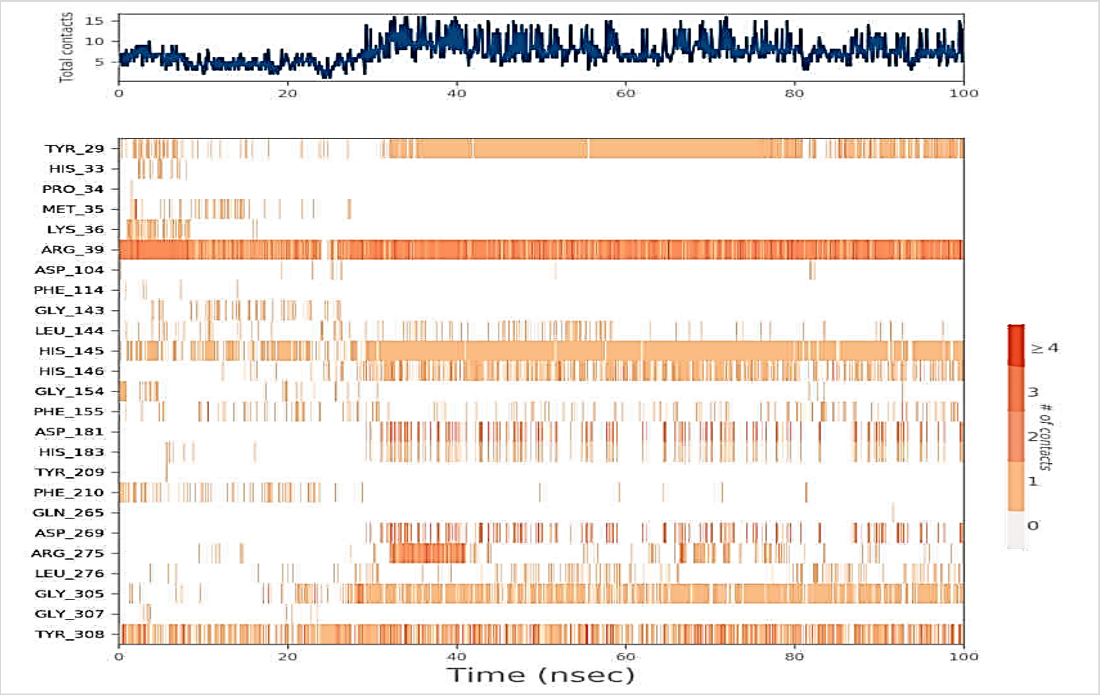

Supplementary Figure 21. Heat map describing the total number of interactions of compound A3bn with HDAC2 complex during the 100 ns simulation period.

Supplementary Table 1. Description, molecular formula, Mwt, mp, and R_f values of the synthesized compounds.

Compound	Molecular formula	Mwt	Description	Yield%	m.p. (°C)	R_f value
**A1**	$C_{11}H_{12}ClNO_4$	257.04	Off-white	75	100-103	0.50[a]
**A2**	$C_9H_{10}ClN_3O_3$	243.041	White to Orange	65	105-106	0.50[b]
**A4**	$C_{10}H_8ClN_3O_3S$	285.70	White to Orange	60	251-253	0.50[a]
**A3an**	$C_{16}H_{13}Cl_2N_3O_3$	366.19	White powder	67	166-168	0.55[d]
**A3bn**	$C_{16}H_{14}ClN_3O_4$	347.75	White powder	60	210-212	0.50[d]
**A4an**	$C_{18}H_{13}ClN_4O_6S$	448.83	yellow powder	80	199-201	0.61[c]
**A4bn**	$C_{18}H_{13}Cl_2N_3O_4S$	438.27	White powder	70	166-168	0.57[c]
**A4cn**	$C_{18}H_{13}BrClN_3O_4S$	482.73	White powder	80	145-148	0.55[c]
**A4dn**	$C_{19}H_{16}ClN_3O_4S$	417.86	White powder	75	176-178	0.50[c]
**A5an**	$C_{16}H_{14}Cl_2N_4O_3S$	413.27	White powder	70	218-220	0.60[a]
**A5bn**	$C_{16}H_{14}BrClN_4O_3S$	457.72	White powder	80	215-217	0.62[c]
**A5cn**	$C_{16}H_{15}ClN_4O_3S$	378.83	White powder	75	207-209	0.58[c]

[a] Mobile phase (n-Hexane / Ethyl acetate 8:2)
[b] Mobile phase (Ethanol / toluene 8:2)
[c] Mobile phase (n-Hexane / Ethyl acetate 6:4)
[d] Mobile phase (n-Hexane / Ethyl acetate 3:7)

Supplementary Table 2. Molecular docking analysis of the tested compounds against HDAC2.

**Compound**	**RMSD value (Å)**	**Docking (Affinity) score (kcal/mol)**
A3an	1.36	-9.41
A3bn	1.66	-9.55
A4	1.68	-9.02
SAHA	1.34	-7.04
Trichostatin	1.13	-8.54

Supplementary Table 3. Molecular docking analysis of the tested compounds against HDAC6.

**Compound**	**RMSD value (Å)**	**Docking (Affinity) score (kcal/mol)**
A3bn	1.73	-8.37
A4cn	1.19	-8.16
A4	0.91	-8.15
SAHA	1.42	-7.86
Trichostatin	1.44	-7.87

Supplementary Table 4. Molecular docking analysis of the tested compounds against HDAC8.

**Compound**	**RMSD value (Å)**	**Docking (Affinity) score (kcal/mol)**
A3bn	1.65	-8.79
A5cn	1.12	-9.32
SAHA	1.17	-8.63
Trichostatin	1.02	-8.23

Supplementary Table 5. Predicted ADMET for the synthesized compounds.

**Compound**	**BBB Level**[a]	**Solubility Level**[b]	**Absorption Level**[c]	**Hepatotoxicity**	**CYP2D6 Prediction**[d]	**PPB Prediction**[e]
A3an	3	2	0	True	False	True
A3bn	4	3	0	True	False	True
A4	3	3	0	True	False	True
A4an	4	2	2	True	False	True
A4bn	4	2	0	True	False	True
A4cn	4	2	1	True	False	True
A4dn	4	2	0	True	False	True
A5an	4	2	0	True	False	True
A5bn	4	2	0	True	False	True
A5cn	4	3	0	True	False	True
SAHA	3	4	0	False	False	False
Trichostatin	2	3	0	True	False	True

[a] BBB level, blood brain barrier level, 0 = very high, 1 = high, 2 = medium, 3 = low, 4 = very low.
[b] Solubility level, 1 = very low, 2 = low, 3 = good, 4 = optimal.
[c] Absorption level, 0 = good, 1 = moderate, 2 = poor, 3 = very poor.
[d] CYP2D6, cytochrome P2D6, True = inhibitor, False = non inhibitor.

Supplementary Table 6. The results of the molecular similarity analysis conducted between SAHA and trichostatin and the tested compounds.

Compound	log p	Mwt	HBA	HBD	Rotatable bonds	Rings	Aromatic rings	MFPSA	Minimum distance	Similar
A3an	3.302	366.199	5	3	6	2	2	0.265	0.960392	True
A3bn	2.396	347.753	6	4	6	2	2	0.333	1.27765	True
A5cn	3.228	378.833	5	5	8	2	2	0.377	1.43402	True
A5an	3.893	413.278	5	5	8	2	2	0.356	1.64429	True
A4	2.498	285.707	6	3	4	2	1	0.453	1.65618	True
Trichostatin	2.772	302.368	4	2	6	1	1	0.2	-	Reference
SAHA	2.005	264.32	3	3	8	1	1	0.276	-	Reference
A5bn	3.977	457.729	5	5	8	2	2	0.352	1.78035	False
A4dn	3.909	417.866	7	2	8	3	3	0.325	1.95231	False
A4bn	4.087	438.285	7	2	8	3	3	0.326	2.01601	False
A4cn	4.171	482.736	7	2	8	3	3	0.322	2.11295	False
A4an	3.317	448.837	9	2	9	3	3	0.422	2.43951	False

Supplementary Table 7. In silico toxicity properties of the newly synthesized compounds.

Compound	FDA Rodent Carcinogenicity (Male, mouse)	Carcinogenic Potency TD_{50} (Rat)[a]	Developmental Toxicity Potential	Rat Oral LD_{50}[b]	Rat Chronic LOAEL[b]	skin and ocular irritation
A3an	Non-Carcinogen	227.97	Non-Toxic	1.122	0.135	Non-irritant
A3bn	Non-Carcinogen	552.69	Non-Toxic	0.329	0.183	Non-irritant
A4	Non-Carcinogen	82.46	Non-Toxic	1.363	0.066	Non-irritant
A4an	Non-Carcinogen	24.42	Non-Toxic	1.183	0.030	Non-irritant
A4bn	Non-Carcinogen	44.27	Non-Toxic	0.475	0.038	Non-irritant
A4cn	Non-Carcinogen	45.41	Non-Toxic	0.778	0.044	Non-irritant
A4dn	Non-Carcinogen	39.58	Non-Toxic	1.562	0.049	Non-irritant
A5an	Non-Carcinogen	291.39	Non-Toxic	1.894	0.113	Non-irritant
A5bn	Non-Carcinogen	300.55	Non-Toxic	3.117	0.129	Non-irritant
A5cn	Non-Carcinogen	1,996.86	Non-Toxic	1.551	0.234	Non-irritant
SAHA	single-Carcinogen	6,846.20	Non-Toxic	8.981	1.527	Non-irritant
Trichostatin	Non-Carcinogen	660.35	Toxic	0.297	0.059	Irritant

[a] Unit: mg/kg body weight/day.
[b] Unit: g/kg body weight.
*Reference

Supplementary Table 8. DFT results of the synthesized compounds showed HOMO and LUMO.

**Compound**	**Total Energy (Kcal/mol)**	**Binding Energy (Kcal/mol)**	**HOMO Energy (Kcal/mol)**	**LUMO Energy (Kcal/mol)**	**Dipole Mag (debye)**	**Band Gap Energy (Kcal/mol)**
A3an	-1916.371	-7.423	-0.198	-0.107	3.33	0.090
A3bn	-4532.938	-7.650	-0.196	-0.101	4.81	0.095
A4	-1624.908	-5.071	-0.201	-0.102	1.27	0.099
A4an	-2208.257	-7.604	-0.209	-0.168	2.35	0.040
A4bn	-2463.352	-7.218	-0.201	-0.127	3.60	0.073
A4cn	-4075.278	-7.201	-0.200	-0.128	3.74	0.073
A4dn	-2044.158	-7.087	-0.199	-0.116	4.38	0.082
A5an	-2368.105	-7.814	-0.194	-0.097	2.02	0.098
A5bn	-4480.032	-7.799	-0.195	-0.096	2.07	0.097
A5cn	-4909.966	-7.866	-0.193	-0.095	2.47	0.098
SAHA	-873.136	-7.319	-0.197	-0.038	0.79	0.159
Trichostatin	-987.530	-8.373	-0.172	-0.075	4.42	0.097

Supplementary Table 91. MM-GBSA energies for compound A3bn with HDAC2 (kcal/mol).

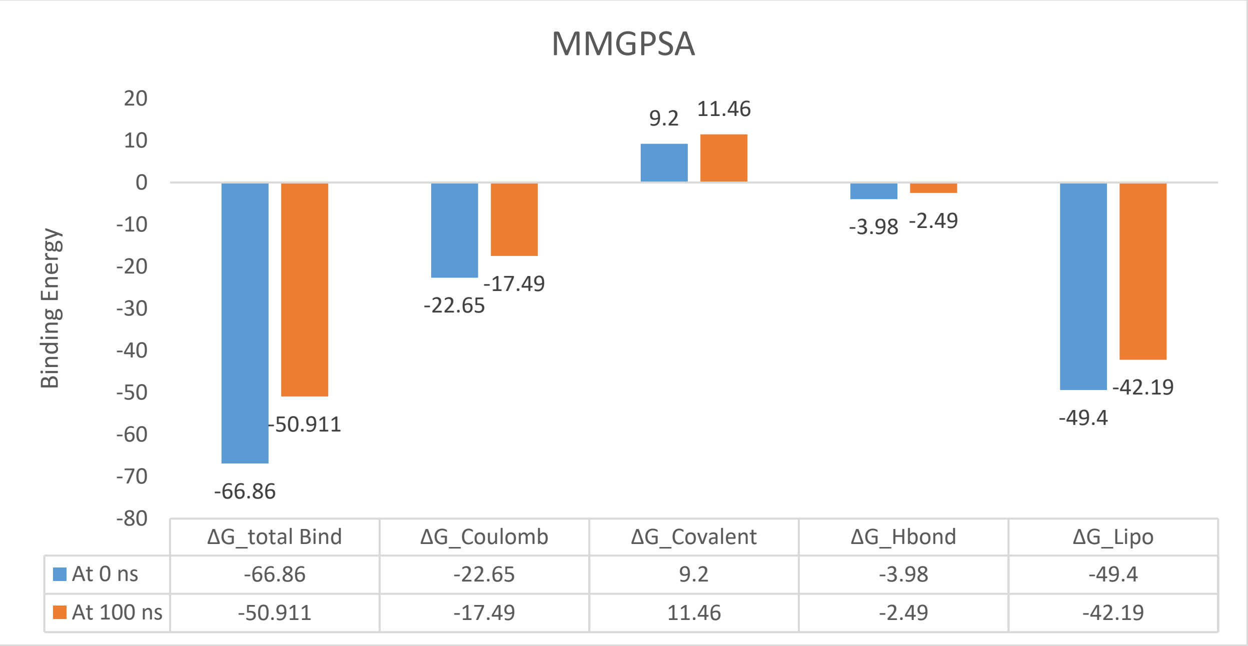

Supplement: Supplementary file 1 — Supporting File 1: cbdv70450‐sup‐0001‐SuppMat.pdf [file CBDV-22-e01492-s001.pdf]
